# Rational Design and Multicomponent Synthesis of Lipid–Peptoid Nanocomposites towards a Customized Drug Delivery System Assembly

**DOI:** 10.3390/molecules28155725

**Published:** 2023-07-28

**Authors:** Thaissa Pasquali F. Rosalba, Guilherme D. R. Matos, Carlos Eduardo M. Salvador, Carlos Kleber Z. Andrade

**Affiliations:** 1 Laboratório de Química Metodológica e Orgânica Sintética (LaQMOS), Instituto de Química, Universidade de Brasília, Campus Universitário Asa Norte, Brasilia 70904-970, Brazil; 2 Laboratório de Modelagem de Sistemas Complexos (LMSC), Instituto de Química, Universidade de Brasília, Campus Universitário Asa Norte, Brasilia 70904-970, Brazil

**Keywords:** Ugi reaction, targeted drug delivery, nanoparticles, lipid–peptoids

## Abstract

Nanotechnology has assumed a significant role over the last decade in the development of various technologies applied to health sciences. This becomes even more evident with its application in controlled drug delivery systems. In this context, peptoids are a promising class of compounds for application as nanocarriers in drug delivery systems. These compounds can be obtained efficiently and with highly functionalized structural diversity via the Ugi 4-component reaction (U-4CR). Herein, we report the design of the process control strategy for the future development of lipid–peptoid-based customized drug delivery system assemblies. Over 20 lipid–peptoid nanocomposites were synthesized via the U-4CR in good to excellent yields. These products were successfully submitted to the nanoparticle formation by the emulsification–evaporation process from lipophilic solution and analyzed via Dynamic Light Scattering (DLS). Several molecules generated nanoparticles with a size ≤200 nm, making them good candidates for drug delivery systems, such as in cancer treatment.

## 1. Introduction

Nanotechnology is an important tool with potential applications in biotechnology, pharmaceuticals, and medicine, and studies related to it have been growing intensely over the past decade [1]. Nanoparticles (NPs) are a wide class of natural or engineered materials with a size range between 10 and 1000 nm [2]. Reducing the particle size of materials to the nanometer scale allows for a significant increase in the total surface area as well as enhanced properties compared with the same larger size material [3]. The use of nanotechnology in medicine has developed a lot in recent years, especially in drug delivery systems. Despite the aforementioned size range for nanoparticles, the preferential size is less than 200 nm for nanomedical applications [3]. 

The production of drug delivery systems through nanocomposites is currently a promising strategy for the development of better methods to diagnose and treat several diseases [4,5,6,7]. These systems combine features such as high bioavailability, greater dosing accuracy, reduced toxicity, controlled release, high dissolution rate, and structural versatility for functionalization to selectively targeted delivery of drugs [7,8]. Despite the great advantages of nanocomposites in drug delivery systems, there are also some challenges and limitations. The large decrease in the size range makes the number of surface atoms increase (larger surface area), leading to problems such as interparticle friction and adhesion [9]. A major drawback of nanotechnology is its dependence on the conditions of the environment surrounding it, which can lead to aggregation or disintegration of the particles, changing their size and resulting in toxicity [10].

In line with this nature of nanocarriers, the design of a model drug delivery system with nanoparticles must include elements that allow the nanoformulation to be able to recognize and reach its therapeutic target, as well as perform the controlled release of pharmacological agents in specific locations [11]. One of the main strategies to obtain this type of nanoformulation is the functionalization of the surface of nanoparticles with the corresponding bioreceptors [12,13]. In this regard, it is possible to modify the nanocomposite with a wide variety of targeting ligands, thus improving properties of interest like bioavailability, biocompatibility, and targeting capacity for a better therapeutic application [14]. However, the main challenge to implementing this strategy is to find a nanocomposite that is flexible to structural changes like size and surface chemistry, as well as being non-cytotoxic and easy to prepare.

In this context, peptoids, oligomers of *N*-substituted alkyl glycines that mimic the primary natural structure of peptides, are a promising class of compounds for the application of targeting strategies for surface functionalization of nanocarriers [15]. Differently from peptides, peptoids have the side chain of the Cα bonded to the nitrogen atom, removing the polar N-H bond of the peptide while keeping the side chain in its structure. Due to this chemical modification, the lipophilicity of the molecule is increased, which may improve membrane permeability [16,17]. These compounds are unnatural mimetic structures of peptides and proteins that have multiple ideal characteristics for a nanocarrier [18], such as resistance to degradation by proteases [19], chemical and thermal stability [20], and high-yielding synthesis via the multicomponent Ugi reaction with enormous structural diversity, mimicking the primary natural structure of peptides [21].

In addition, these compounds are ideal for the recognition of target-sequence-specific molecules and for the control of self-assembling nanostructures, due to structural aspects related to the loss of the hydrogen bond donor in the backbone and to the non-chirality of the main chain, in comparison with the peptide structure [22,23]. Hence, the intermolecular interactions between the peptoid chains occur by superficial adjustment through interactions exclusively between the side chains linked to the nitrogen of the amide bond [24]. Thus, these compounds have structures similar to liposomal nanocomposites [25], which are widely applied in drug delivery systems [26].

One of the most relevant methods for the construction of a peptoid backbone is the Ugi four-component reaction (U-4CR) [27]. This multicomponent reaction (MCR) consists of the condensation of an amine, an aldehyde or ketone, an isocyanide, and a carboxylic acid to form α-acylaminoamides in a one-pot reaction [28]. Among the many advantages of this reaction, its versatility stands out, playing a significant role in modern drug discovery [27]. Although there are some examples of peptoid nanoparticles in the literature, very few use MCRs to obtain these compounds [29].

Herein, we describe a fundamental model proposal for the future development of lipid–peptoid-based customized drug delivery systems. This proposal was built based on the synthesis of a wide variety of peptoids with structural diversity via the Ugi reaction, followed by nanoformulations through the emulsification–evaporation process and molecular dynamics simulations.

## 2. Results and Discussion

It is well known that some of the best advantages of MCRs are their versatility, accelerating synthesis, screening, and property optimization of large compound libraries in just one step [27,30]. Another great benefit of MCRs is the use of optimized methodologies, such as microwave heating, making it possible to decrease the reaction time to a few minutes [31]. With that in mind, we decided to take advantage of these two powerful tools in modern organic synthesis and synthesize a diversity of lipid–peptoids via the U-4CR according to previously known conditions within our research group [32,33,34,35,36,37]. These conditions involved MeOH as a solvent and microwave heating at 80 °C for only 10 minutes.

**Scheme 1:**
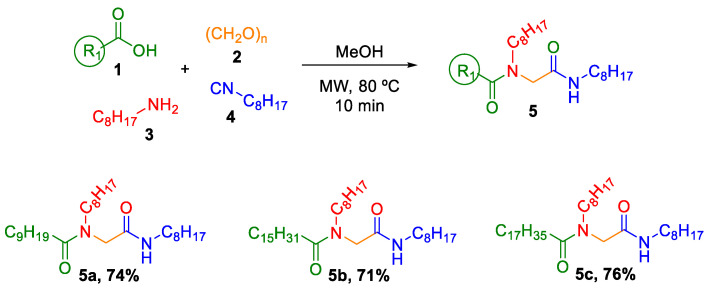
Peptoid synthesis via the Ugi reaction between *n*-octyl isocyanide, *n*-octyl amine, paraformaldehyde, and different fatty acids.

The synthesis of the lipid–peptoids was carried out via the U-4CR using a fatty acid **1**, a lipidic isocyanide **4**, a lipidic amine **3**, and paraformaldehyde **2** as the oxo component, avoiding the formation of stereocenters. At first, *n*-octyl isocyanide **4**, which was prepared according to the literature [38], *n*-octyl amine **3**, and decanoic acid were used to synthesize compound **5a** (Scheme 1), believing that the long chains with ten and eight carbons would be long enough to prepare a nanoparticle. 

Molecule **5a** was submitted to the nanoparticle formation by the emulsification–evaporation process from lipophilic solution, where the Ugi product was dissolved in acetone and the organic phase was dropped into an aqueous phase containing polysorbate 80 as the stabilizer [39]. After total evaporation of the organic solvent, the remaining emulsion was analyzed via Dynamic Light Scattering—DLS, which furnished the cumulative information about particle size through the hydrodynamic diameter (DH) and solution homogeneity via the polydispersity index (PDI), which represents the size distribution in the solution. The value for PDI ranges from 0.0 to 1.0.

**Figure 1:**
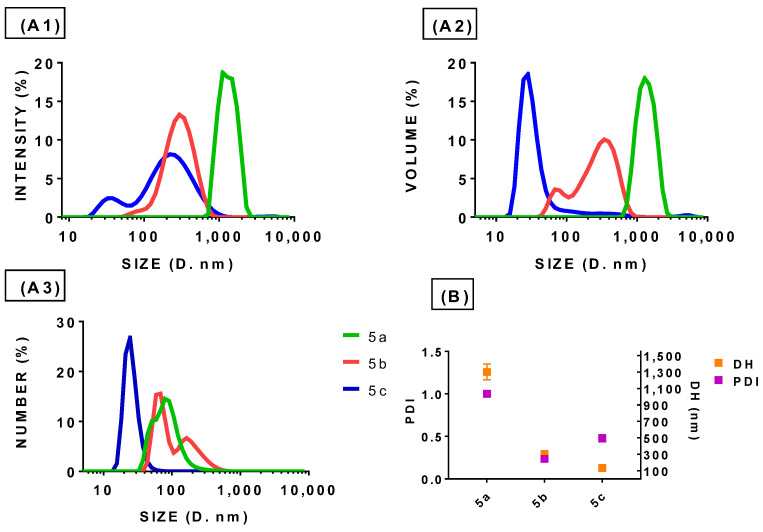
DLS size distributions by (**A1**) intensity, (**A2**) volume, and (**A3**) number for compounds **5a**–**c**. (**B**) PDI and DH for compounds **5a**–**c**.

The results showed large particle sizes of ∼1300 nm (DH) and PDI of 1.000 (Figure 1B), indicating that compound **5a** is not a good candidate to prepare nanoparticles. An efficient method of improving the results of nanoparticle formation is to increase the long chains in the molecule. Regarding lipid matrix nanoparticles (LNPs), fatty acids are among the most commonly used lipids [2]. Thus, carboxylic acid was chosen as the component to be screened and was replaced by palmitic acid. The new molecule **5b** was submitted to the same nanoparticle formation process and analyzed. As expected, now the nanoparticles were obtained with a size of ∼250 nm and PDI: 0.200, indicating a more uniform sample in terms of particle size (Figure 1B). Encouraged by these results, stearic acid was used in the U-4CR, believing that an even longer chain would further decrease the size of the nanoparticles. Indeed, to our delight, this was confirmed and molecule **5c** showed the best results so far, with a nanoparticle size of ∼130 nm and PDI: 0.470 (Figure 1B). Then, molecule **5c** was submitted to the same nanoparticle formation procedure but without the presence of a surfactant. In this experiment, no nanoparticles were formed, confirming that it is essential to have a stabilizer agent in the solution. 

**Figure 2:**
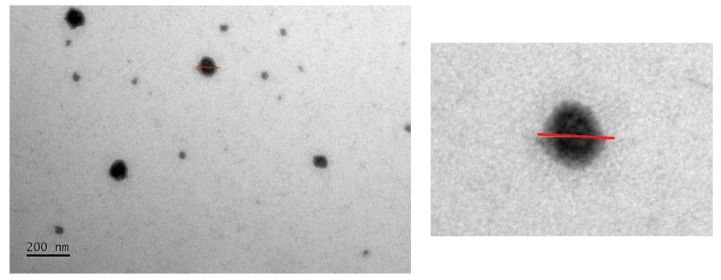
Transmission electron microscopy (TEM) images of 5c nanoparticles. Magnification of 15k. Particle size of 91.608 nm (red line).

Analyzing the graphics in Figure 1A, the results obtained for the three molecules can be compared. For **5a**, the intensity and the volume showed only particles with a size above 1000 nm. For **5b**, despite achieving a size in the range of ∼200 nm, the graphic indicates a broad particle size distribution. For **5c**, in terms of intensity, the graphic suggests aggregates with particles size below and above 100 nm. When it was converted to volume, larger aggregates were observed but in low concentration. The main single sharp peak indicates the existence of most of the uniform population below 100 nm. In TEM images, spherical-shaped particles were observed, with an average size of 91.608 nm (Figure 2). It can be observed that polysorbate 80 is the coating on the nanoparticles, endorsing that the stabilizer is important to the formation of nanoparticles. The regular size pattern observed in the images matches the ones observed in DLS measurements.

**Scheme 2:**
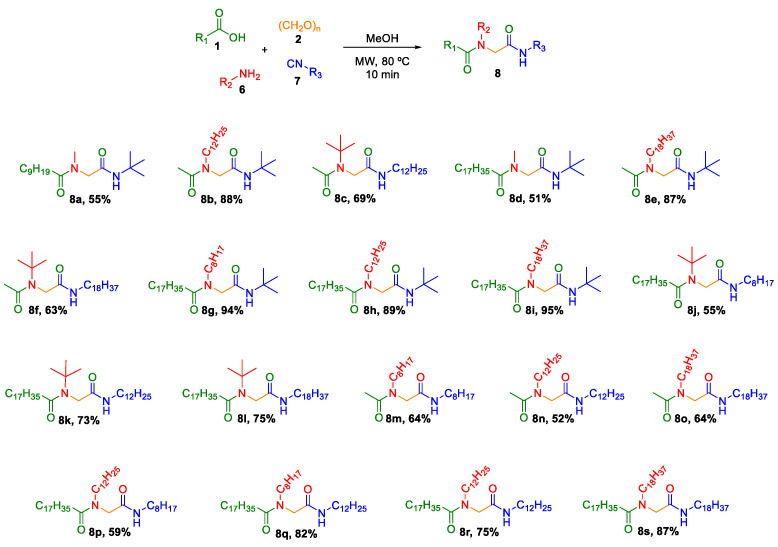
Scope of the lipid–peptoids via the Ugi reaction.

These initial results raised some questions about the influence of the structure of the Ugi adducts on nanoparticle formation. Is the long chain at the acid position essential to prepare the nanoparticles? Could the other chains be removed, simplifying the structure of the molecules? Since increasing the number of carbons in the carboxylic acid long chain led to better results, the reaction scope was next evaluated, performing a structural study of all the long chains in different positions in the structure of the Ugi products. Therefore, a library of lipid–peptoids was synthesized by varying the position (R_1_, R_2_, and R_3_), the size (C_8_, C_12_, and C_18_), and the number of long chains in the U-4CR (Scheme 2). In total, 19 different adducts were isolated in good to excellent yields (51–95%). At first, *n*-dodecyl isocyanide and *n*-octadecyl isocyanide were synthesized in high yields following the same methodology described for the *n*-octyl isocyanide. These isocyanides had already been described in the literature and were proved to be as reactive as other aliphatic isocyanides [40].

**Figure 3:**
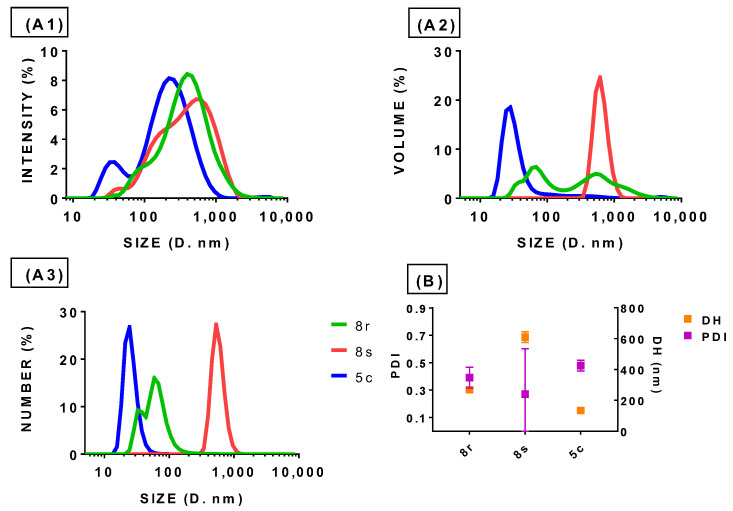
DLS size distributions by (**A1**) intensity, (**A2**) volume, and (**A3**) number for compounds **8r**, **8s**, and **5c**. (**B**) PDI and DH for compounds **8r**, **8s**, and **5c**.

Again, all the products were submitted to the nanoparticle formation by the same process used for the first three molecules and analyzed via DLS to compare their relative size and identify which features induce the formation of the nanoparticles. Surprisingly, raising all the long chains (**8r**-C_12_ and **8s**-C_18_) did not reduce the size compared with our initial best result of compound **5c**. On the contrary, in compound **8s**, where the carboxylic acid, the amine, and the isocyanide used in the reaction have a long C_18_ chain, the size of the nanoparticles was ∼600 nm (Figure 3B). Comparing the graphics for **8r**, **8s**, and **5c**, one can see that **5c** is still the best candidate for preparing nanoparticles since it is the one with a uniform volume of particles below 100 nm (Figure 3A2).

**Figure 4:**
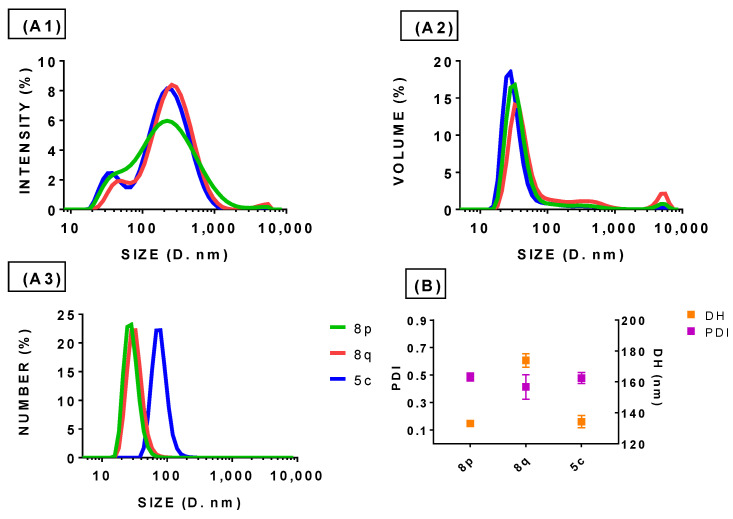
DLS size distributions by (**A1**) intensity, (**A2**) volume, and (**A3**) number for compounds **8p**, **8q**, and **5c**. (**B**) PDI and DH for compounds **8p**, **8q**, and **5c**.

Since the size of the nanoparticles for compound **8r**, which has two long C_12_ chains (amine and isocyanide), was ∼260 nm (a relatively good size, since the preferential size for nanomedical application is less than 200 nm), it was decided to keep the C_18_ carboxylic acid and replace *n*-dodecyl isocyanide for *n*-octyl isocyanide (**8p**), and *n*-dodecyl amine for *n*-octyl amine (**8q**), mixing the C_12_ and C_8_ long chains in the structures. Remarkably, both gave excellent results, where the sizes of the nanoparticles were ∼130 nm and ∼170 nm, respectively. As can be observed in all graphics in Figure 4A1–A3, the curves of the three molecules had a good overlap. 

**Figure 5:**
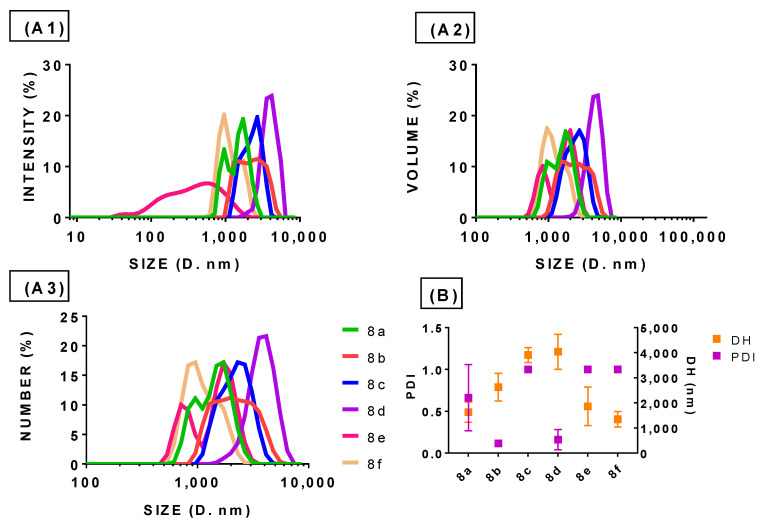
DLS size distributions by (**A1**) intensity, (**A2**) volume, and (**A3**) number for compounds **8a**–**f**. (**B**) PDI and DH for compounds **8a**–**f**.

Molecules with only one long chain in their structures, in which the positions between the acid, the amine, and the isocyanide were exchanged, did not furnish nanoparticles, as the particle sizes for all six molecules were above 1000 nm (Figure 5). For the C_12_ (C_10_ for carboxylic acid) long chains, the particle sizes were ∼2000 nm (**8a**), ∼2500 nm (**8b**), and ∼3500 nm (**8c**). For the C_18_ long chains, the particle sizes were ∼4100 nm (**8d**), ∼2000 nm (**8e**), and ∼1200 nm (**8f**). Regarding the observed results, it was postulated that only one long chain in the structure is not enough to prepare nanoparticles of these molecules. 

**Figure 6:**
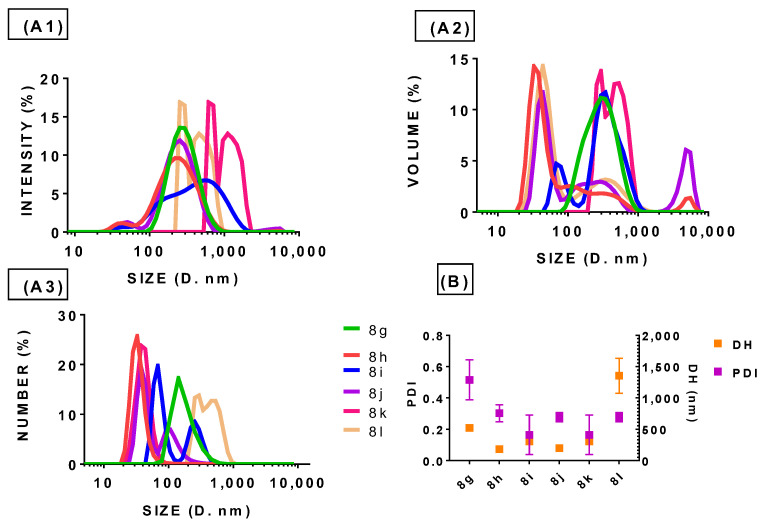
DLS size distributions by (**A1**) intensity, (**A2**) volume, and (**A3**) number for compounds **8g**–**l**. (**B**) PDI and DH for compounds **8g**–**l**.

On the other hand, when the molecules had two long chains in their structures, the results were improved and most of the nanoparticle sizes were below 300 nm (Figure 6B). Towards these structural analyses, the molecules were divided into three groups according to the position of the long chains: acid and amine, acid and isocyanide, and amine and isocyanide. In the first two groups, stearic acid was preserved and the long chains in the amine (**8g**, **8h**, and **8i**) and in the isocyanide (**8j**, **8k**, and **8l**) were varied between C_8_, C_12_, and C_18_ in each group. These six compounds showed good nanoparticle sizes (between ∼200 nm and ∼300 nm). 

**Figure 7:**
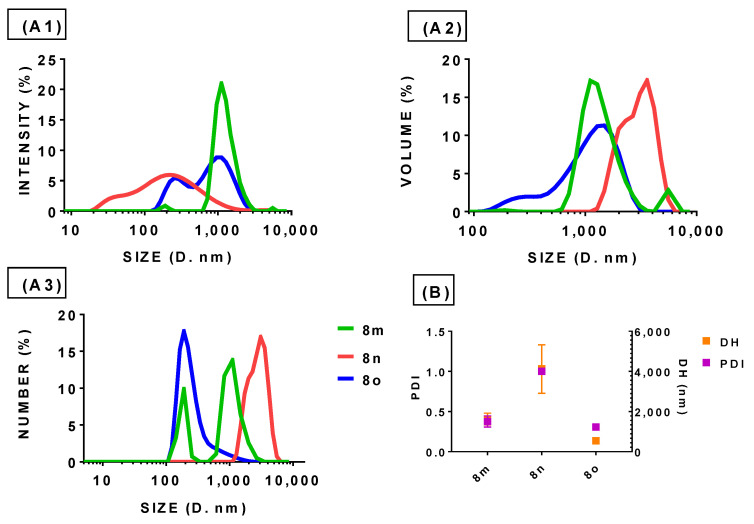
DLS size distributions by (**A1**) intensity, (**A2**) volume, and (**A3**) number for compounds **8m**–**o**. (**B**) PDI and DH for compounds **8m**–**o**.

In contrast, when the long chain from the acid position was removed, nanoparticles were no longer obtained. In the third group, stearic acid was replaced with acetic acid and the long chains in the amine and isocyanide were varied between C_8_, C_12_, and C_18_. For compound **8o**, the size of the nanoparticles was ∼500 nm. But for the other two compounds (**8m** and **8n**), the particle sizes were above 1000 nm (Figure 7B). These results once again reassure the idea that carboxylic acids with long chains are essential for the success of nanoparticle preparation. 

These results showed that two long chains in the structure of the Ugi adducts are enough to prepare nanoparticles. The position of the second long chain does not matter since it is possible to use a lipidic amine or a lipidic isocyanide in combination with the fatty acid. These are excellent results because they offer a new prospect for the combinatorial synthesis of functionalized lipid–peptoid nanocomposites as they allow for the fast variation in the components, facilitating the incorporation of molecules on the surface of the nanoparticles, making them good target-specific nanocarrier systems. Specific substrates that have an affinity to a biological target of interest can be used as different starting materials in the Ugi reaction. In this way, we can take advantage of one of the great features of MCRs: their versatility. 

In light of the diverse structural patterns exhibited by peptoid-based nanoparticles [29], simulations of a limited quantity of Ugi adducts in an aqueous environment were carried out with the objective of elucidating the assembly process of the nanoparticle. Our investigations involved performing 30-nanosecond molecular dynamics (MDs) simulations on a system comprising 50 units of adducts, namely, **5c** and **8r**, in water. The obtained results revealed a notable tendency of longer aliphatic chains to aggregate, as expected.

**Figure 8:**
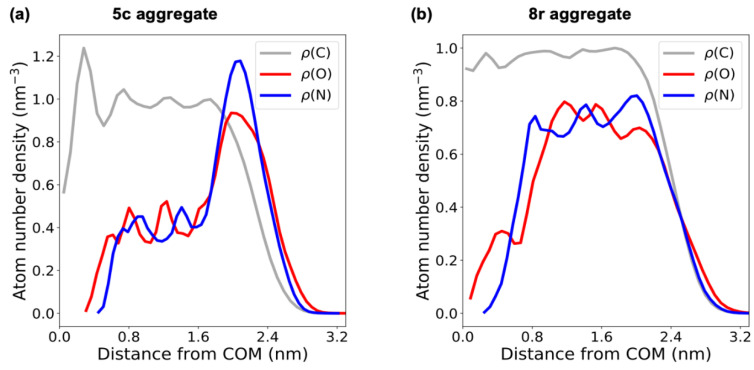
Atom number densities (ρ) calculated with respect to the center of mass (COM) of **5c** and **8r** aggregates for the 30 ns MD trajectories. The grey curves correspond to the number density of carbon atoms, which in **5c** and **8r** are mostly in aliphatic chains. The red and blue curves correspond to oxygen and nitrogen densities, respectively. Comparison between (**a**) and (**b**) shows that **5c** exposes more oxygen and nitrogen atoms than **8r**.

The aggregation phenomenon through van der Waals forces should not be regarded as a conclusive indication of nanoparticle formation. Instead, it offers insights into the spatial arrangement of the nanoparticle constituents and their interaction with the surrounding medium. As depicted in Figure 8, the analysis reveals distinctive characteristics for adduct **5c** (comprising C_8_, C_8_, and C_18_ chains) and adduct **8r** (C_12_, C_12_, and C_18_ chains) aggregates. Notably, adduct **5c** aggregate exhibits a concentrated distribution of nitrogen and oxygen atoms near its surface, with a peak density observed at approximately 2 nm. In contrast, adduct **8r** aggregate displays a more uniform density distribution extending over a broad range, spanning from 0.8 to 2.2 nm. These findings suggest that the relative size of the aliphatic chains may exert an influence on the potential formation of liposome-like nanoparticles. Specifically, the presence of a singular longer chain relative to the others could play a role in the development of such nanoparticles.

**Figure 9:**
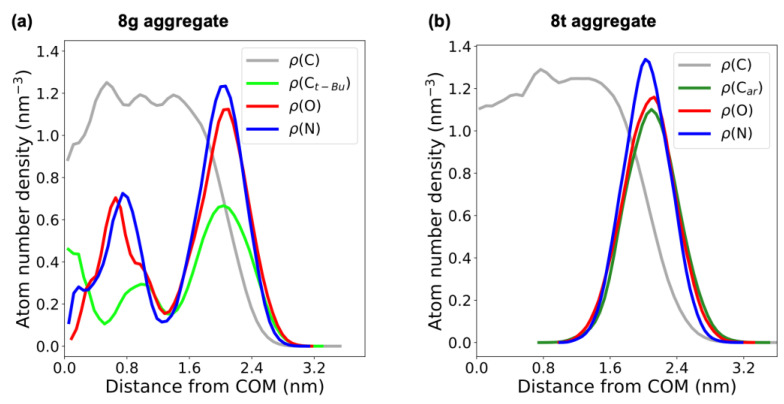
Atom number densities (ρ) calculated with respect to the center of mass (COM) of **8g** and **8t** aggregates for the 30 ns MD trajectories. Grey curves correspond to carbon atoms belonging to long aliphatic chains and red and blue curves correspond to oxygen and nitrogen atoms, respectively. Light green corresponds to *t*-butyl carbon atoms and dark green corresponds to aromatic carbon atoms.

Figure 9 suggests the possibility of substituting one of the chains with different functional groups, which may improve the density profile of the aggregates. Further molecular dynamics simulations were conducted on adducts **8g** and **8t**, wherein the isocyanide moieties contained *t*-butyl and phenyl groups, respectively. The results of these simulations reveal that the aggregates can position bulkier or less hydrophobic segments of their constituents on the surface, as illustrated in Figure 9. These outcomes suggest that the incorporation of charged or dipolar groups may serve as a viable strategy to render peptoid liposomes more akin to phospholipid liposomes in terms of their properties and characteristics.

**Figure 10:**
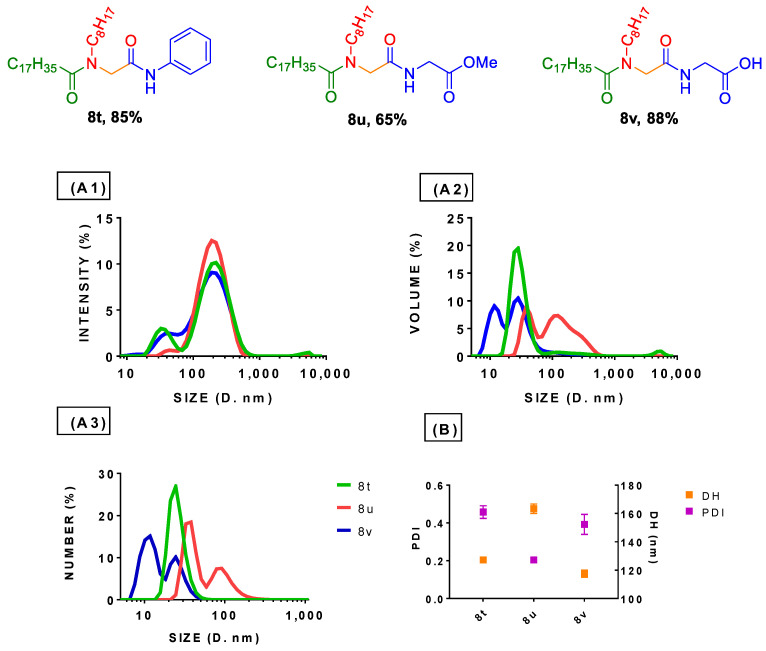
New molecules synthesized by placing a dipolar group in the isocyanide position and DLS size distributions by (**A1**) intensity, (**A2**) volume, and (**A3**) number for compounds **8t**–**v**. (**B**) PDI and DH for compounds **8t**–**v**.

Molecular dynamics simulations of “toy” systems (50 molecules in water) suggest that placing a dipolar group in the isocyanide would favor liposome formation since the long aliphatic chains interact with each other inside the nanoparticle bilayer and the dipolar group interacts with the water molecules outside the membrane. Figure 9a shows that *t*-butyl groups belonging to **8g** tend to concentrate along the surface of the aggregate (peak approximately at 2 nm). *t*-Butyl hydrophobicity may be the cause of the smaller oxygen and nitrogen density peaks under 1.6 nm, but it is possible that the MD simulation was not long enough to allow the system to properly relax and reach equilibrium. Figure 9b shows that aromatic carbon atoms tend to place themselves along the surface of the aggregate. Inspired by these simulations, three new molecules were synthesized using phenyl isocyanide (**8t**) and methyl isocyanoacetate (**8u**). Compound **8v** was obtained from the hydrolysis of the Ugi product **8u**. Confirming the assumption made by the analysis of the simulations, all three compounds showed excellent nanoparticle sizes (below 170 nm) (Figure 10). 

Figure 10A1 shows an overlap of the curves in the three molecules in the graphic for DLS size distribution by intensity, indicating that they have a similar profile. However, the graphic for volume shows that only molecule **8t** has a homogeneous size distribution since it appears as a single sharp peak with a size below 100 nm (Figure 10A2). For the other two molecules (**8u** and **8v**), the graphic indicates a broad particle size distribution. This is confirmed in Figure 10B by the PDI values. Despite the graphics for compound **8v** indicating that the nanoparticle size distribution is the smallest so far, the homogeneity still needs to be improved. 

**Scheme 3:**
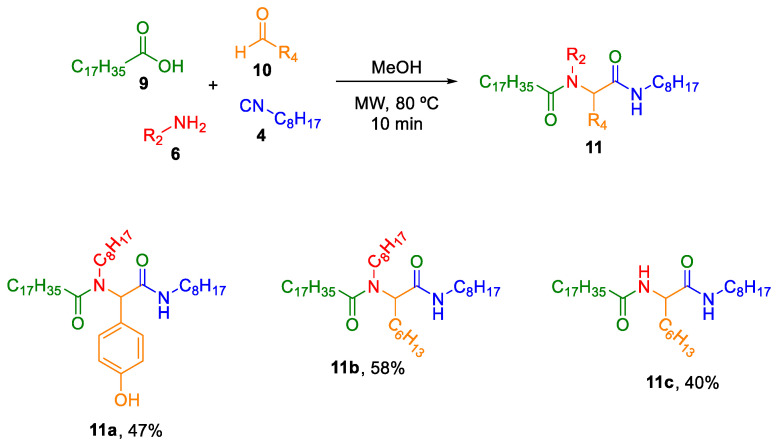
Ugi reaction between stearic acid, *n*-octyl isocyanide, *n*-octyl amine (or ammonia), and different aldehydes.

To complete the structural study, paraformaldehyde was replaced by aromatic and aliphatic aldehydes to analyze if a group different from hydrogen in this position would affect the formation of nanoparticles (Scheme 3). The synthesis of these three new compounds was accomplished by the same protocol and the Ugi products were obtained in somewhat lower yields (40–58%), which can be tentatively accounted as follows: **11a** showed problems in the purification step as it has the same R_f_ of *p*-OH-benzaldehyde, requiring a base extraction (NaOH) to remove the phenol. **11c**, **8a**, and **8d** were synthesized using an organic solution of the amines (they are gases at room temperature).

**Figure 11:**
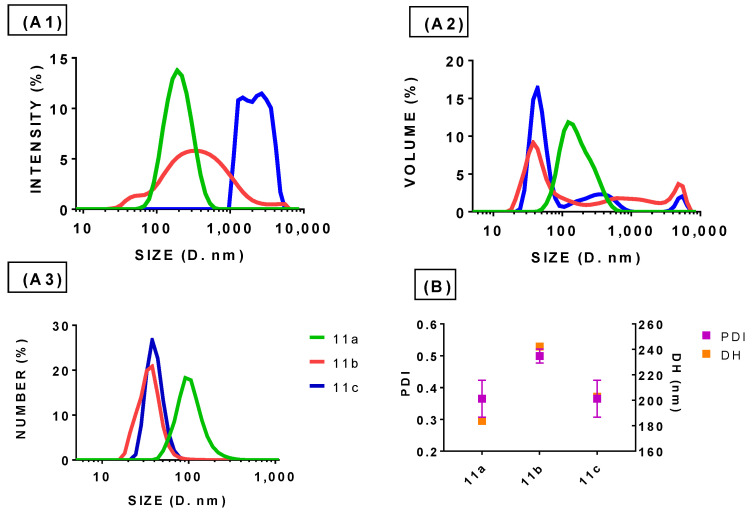
DLS size distributions by (**A1**) intensity, (**A2**) volume, and (**A3**) number for compounds **11a**–**c**. (**B**) PDI and DH for compounds **11a**–**c**.

All compounds showed good nanoparticle size (∼200 nm) as can be seen in Figure 11. These results confirm even more the magnitude of the diversity-oriented synthetic method provided by the U-4CR. Now, one more position in the structure of the lipid–peptoid nanocomposites can be functionalized, maintaining the nanoparticle sizes. 

Compounds **11a** and **11b** contain an aromatic and an aliphatic substituent, respectively, at the stereocenter. The three graphics for **11a** show a more homogeneous size distribution compared with **11b**, especially regarding the volume (Figure 11A1–A3). These results are in agreement with the theory that a dipolar group or less lipophilic group interacts with the water molecules outside the membrane in the nanoparticle bilayer, promoting the development of the nanoparticles, as indicated by the molecular dynamics simulations.

Formulations with nanoparticles prepared for DLS analysis using the different Ugi products showed a homogeneous white-bluish opalescent aspect and zeta potential values from −11.2 to +1.90 mV, except for compound **8v**, which showed a homogeneous clear aspect and zeta potential value of −20.50 mV. The zeta potential values were close to zero; this was probably due to the steric effect caused by polysorbate 80, which is the coating on the nanoparticles. Table 1 summarizes the differences in the composition and properties (average particle size and PDI) of the obtained peptoids along with the yields for each case. 

## 3. Materials and Methods

Reactions were performed on a Biotage^®^ Initiator+ (Uppsala, Sweden) microwave reactor using sealed vessels, temperature detection via an internal fiber optic probe, simultaneous cooling, and media stirring. Commercially available reagents and solvents were analytical grade or were purified via standard procedures prior to use. All reactions were monitored via thin-layer chromatography (TLC) and revealed by treatment with a 10% solution of phosphomolybdic acid in ethanol (PMA), followed by heating. Column chromatography was performed on silica gel (70–230 mesh) and the solvents used as eluents are described for each molecule. Nuclear magnetic resonance spectra (NMR) were recorded on a Bruker Avance 600 spectrometer (Billerica, MA, USA) (^1^H NMR (600 MHz), ^13^C NMR (151 MHz)) at 25 °C with TMS as an internal standard for CDCl_3_ as solvent. DMSO-d6 was also used as a solvent in some cases. High-resolution mass spectra (HRMS) were performed on a Triple Tof 5600 Sciex via flow injection analysis using an Eksigent UltraLC 100 Sciex chromatography (Billerica, MA, USA) set to a flow rate of 0.3 mL/min. A DuoSpray Ion Source—ESI (Billerica, MA, USA) was used, and the spectra were acquired in positive mode. The ^1^H and ^13^C NMR, and mass spectra for each structure, are available in the Supplementary Materials. Melting points were measured on a micro melting point apparatus and uncorrected. Dynamic Light Scattering (DLS) was performed using a Zetasizer Nano S90 (Malvern Instruments Ltd., Worcestershire, UK). Each size measurement consisted of three runs to yield an average and standard deviation at 25 °C. All DLS data were collected and analyzed using Zetasizer software 8.01.4906. The hydrodynamic diameter (z-average) and polydispersity index (PDI) determined via DLS were obtained via cumulant analysis and referred to as effective diameters. In addition, the lipid NPs were analyzed via transmission electron microscopy (TEM; JEM-1011, JEOL, Tokyo, Japan) for their diameter and morphology. For TEM measurement, a 5 µL drop of diluted nanoparticle suspensions (water) was deposited onto carbon-coated copper grids and negatively stained with 5 µL of phosphotungstic acid (wt 2%) for 2 min. Finally, the droplet was dried for 24 h.

### 3.1. Molecular Dynamics Simulation Details

Molecules **5c**, **8g**, **8r**, and **8t** were drawn using UCSF Chimera [41]. Each one was separately replicated 50 times in simulation cubes of 40 Å side using Packmol [42]. AM1-BCC charges [43,44] and GAFF2 force field [45,46] parameters were assigned to each molecule using AmberTools22 Antechamber [47,48]. AmberTools TLEAP was used to solvate the system with TIP3P water molecules [49] and to convert the resulting files to the GROMACS format [50] using ParmEd [51]. In each system, energy minimization was run with a steepest descent algorithm with an energy tolerance of 10 kJ/mol and step sizes of 0.01 kJ/mol. 

Molecular dynamics simulations were run using GROMACS 2023.1 in four stages: a 100 ps NVT simulation with a Langevin integrator set to generate configurations at 298 K with a step size of 2 fs; a 2 ns NPT simulation at 298 K with a Berendsen barostat to bring the density and the pressure of the simulation box to near-equilibrium conditions at 1 bar; a 2 ns NPT simulation at 298 K with a Parrinello–Rahman barostat to ensure that sampling is adequate in the isothermal–isobaric ensemble; and a 30 ns NPT simulation at 1 bar and 298 K using the same integrator and barostat of the preceding stage. Bond lengths were kept fixed (‘constraint = all_bonds’) because we were interested in how the peptoids would ultimately organize themselves in water. Van der Waals interactions were neglected beyond a cutoff of 12 Å with a switch at 10 Å, and electrostatic interactions were calculated using the Particle Mesh Ewald (PME) method of order 4 with a real-space cutoff of 12 Å and grid spacing of 1.6 Å. Periodic boundary conditions were applied.

### 3.2. Synthetic Procedures

#### 3.2.1. General Procedure for the Ugi Reactions


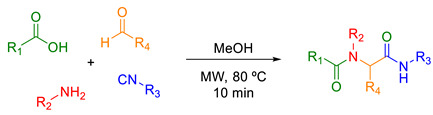


A Biotage microwave reaction vial of 0.5–2.0 mL containing a mixture of carboxylic acid (0.50 mmol), aldehyde (0.50 mmol), amine (0.50 mmol), and isocyanide (0.50 mmol) in methanol (1.5 mL) was introduced in the cavity of a microwave reactor (Biotage^®^ Initiator^+^, Uppsala, Sweden) and irradiated at 80 °C for 10 min under magnetic stirring. The reaction mixture was concentrated under vacuum and purified via column chromatography. The detailed procedures are described for each molecule.

*N-Octyl-N-(2-(octylamino)-2-oxoethyl)decanamide* (**5a**) was obtained from capric acid (0.50 mmol, 0.086 g), paraformaldehyde (0.50 mmol, 0.015 g), *n*-octyl amine (0.50 mmol, 0.081 g), and *n*-octyl isocyanide (0.50 mmol, 0.089 mL), following the general procedure for Ugi reactions, in 74% yield (0.166 g) as a white solid after silica gel column chromatography (100% hexano → 20% ethyl acetate/hexane). R_f_ = 0.25 (20% ethyl acetate/hexane); m.p. 59–60 °C. ^1^H NMR (600 MHz, CDCl_3_) δ 6.76 (s, 1H), 3.97 (s, 2H), 3.41–3.32 (m, 2H), 3.24–3.13 (m, 2H), 2.45–2.30 (m, 2H), 1.75–1.65 (m, 2H), 1.62–1.53 (m, 2H), 1.50–1.44 (m, 2H), 1.29 (s, 32H), 0.90 (t, *J* = 7.0, 9H). ^13^C NMR (151 MHz, CDCl_3_) δ 174.4, 169.9, 51.5, 50.0, 39.4, 32.9, 31.9, 31.8, 31.7, 29.5, 29.5, 29.4, 29.3, 29.25, 29.23, 29.21, 28.8, 26.9, 26.7, 25.4, 22.7, 22.65, 22.61, 14.10, 14.08, 14.07. HRMS (ESI-TOF) *m*/*z* calculated for C_28_H_56_N_2_O_2_ + H^+^: 453.4420 [M + H]^+^; found 453.4412.
*N-Octyl-N-(2-(octylamino)-2-oxoethyl)palmitamide* (**5b**) was obtained from palmitic acid (0.50 mmol, 0.128 mg), paraformaldehyde (0.50 mmol, 0.015 g), *n*-octyl amine (0.50 mmol, 0.081 g), and *n*-octyl isocyanide (0.50 mmol, 0.089 mL), following the general procedure for Ugi reactions, in 84% yield (0.223 g) as a white solid after silica gel column chromatography (100% hexane → 30% ethyl acetate/hexane). R_f_ = 0.37 (20% ethyl acetate/hexane); m.p. 57–59 °C. ^1^H NMR (600 MHz, CDCl_3_) δ 4.02–3.93 (m, 2H), 3.40–3.33 (m, 2H), 3.23–3.16 (m, 2H), 2.41–2.33 (m, 2H), 1.69–1.56 (m, 4H), 1.52–1.45 (m, 2H), 1.37–1.23 (m, 46H), 0.90 (t, *J* = 7.0, 9H). ^13^C NMR (151 MHz, CDCl_3_) δ 173.9, 171.5, 51.9, 51.4, 50.0, 48.6, 39.5, 33.4, 32.9, 31.9, 31.8, 31.7, 29.70, 29.68, 29.66, 29.55, 29.46, 29.36, 29.25, 29.24, 29.21, 28.7, 27.5, 26.9, 26.7, 25.4, 25.1, 22.69, 22.65, 22.61, 14.12, 14.08, 14.07, 11.9. HRMS (ESI-TOF) *m*/*z* calculated for C_34_H_68_N_2_O_2_ + H^+^: 537.5359 [M + H]^+^; found 537.5354.
*N-Octyl-N-(2-(octylamino)-2-oxoethyl)stearamide* (**5c**) was obtained from stearic acid (0.50 mmol, 0.142 g), paraformaldehyde (0.50 mmol, 0.015 g), *n*-octyl amine (0.50 mmol, 0.081 g), and *n*-octyl isocyanide (0.50 mmol, 0.089 mL), following the general procedure for Ugi reactions, in 76% yield (0.214 g) as a white solid after silica gel column chromatography (100% hexane → 30% ethyl acetate/hexane). R_f_ = 0.35 (20% ethyl acetate/hexane); m.p. 54–56 °C. ^1^H NMR (600 MHz, CDCl_3_) δ 4.03–3.95 (m, 2H), 3.40–3.32 (m, 2H), 3.28–3.18 (m, 2H), 2.40–2.33 (m, 2H), 1.70–1.56 (m, 4H), 1.35–1.23 (m, 48H), 0.93–0.86 (m, 9H). ^13^C NMR (151 MHz, CDCl_3_) δ 174.5, 169.8, 51.4, 50.1, 39.6, 32.9, 31.9, 31.8, 31.7, 29.71, 29.68, 29.66, 29.55, 29.46, 29.41, 29.37, 29.24, 29.21, 28.8, 26.9, 26.7, 25.4, 22.69, 22.65, 22.62, 14.12, 14.08. HRMS (ESI-TOF) *m*/*z* calculated for C_36_H_72_N_2_O_2_ + H^+^: 565.5672 [M + H]^+^; found: 565.5668.
*N-(2-(tert-Butylamino)-2-oxoethyl)-N-methyldecanamide* (**8a**) was obtained from capric acid (0.50 mmol, 0.086 g), paraformaldehyde (0.50 mmol, 0.015 g), methyl amine 2M in THF (0.50 mmol, 0.250 mL), and *t*-butyl isocyanide (0.50 mmol, 0.056 mL), following the general procedure for Ugi reactions, in 55% yield (0.082 g) as a colorless oil after silica gel column chromatography (20% ethyl acetate/hexane → 50% ethyl acetate/hexane). R_f_ = 0.60 (50% ethyl acetate). ^1^H NMR (600 MHz, CDCl_3_) δ 6.28 (s, 1H), 3.94–3.88 (m, 2H), 3.11 (s, 3H), 2.42–2.32 (m, 2H), 1.68–1.60 (m, 2H), 1.34–1.23 (m, 21H), 0.88 (t, *J* = 7.0 Hz, 3H). ^13^C NMR (151 MHz, CDCl_3_) δ 174.4, 168.5, 54.5, 53.4, 51.3, 36.9, 33.2, 31.8, 29.5, 29.42, 29.38, 29.26, 28.75, 28.68, 25.1, 22.6, 14.1. HRMS (ESI-TOF) *m*/*z* calculated for C_17_H_34_N_2_O_2_ + H^+^: 299.2699 [M + H]^+^; found: 299.2691.
*N-(tert-Butyl)-2-(N-dodecylacetamido)acetamide* (**8b**) was obtained from acetic acid (0.50 mmol, 0.030 mL), paraformaldehyde (0.50 mmol, 0.015 g), *n*-dodecyl amine (0.50 mmol, 0.092 g), and *t*-butyl isocyanide (0.50 mmol, 0.056 mL), following the general procedure for Ugi reactions, in 88% yield (0.150 g) as a colorless oil after silica gel column chromatography (20% ethyl acetate/hexane → 50% ethyl acetate/hexane). R_f_ = 0.35 (30% ethyl acetate/hexane). ^1^H NMR (600 MHz, CDCl_3_) δ 6.54 (s, 1H), 3.90–3.85 (m, 2H), 3.40–3.28 (m, 2H), 2.16 (s, 3H), 1.64–1.57 (m, 2H), 1.32 (s, 9H), 1.28–1.20 (m, 18H), 0.89 (t, *J* = 7.0, 3H). ^13^C NMR (151 MHz, CDCl_3_) δ 171.4, 168.9, 52.2, 51.1, 50.8, 31.9, 29.55, 29.54, 29.48, 29.3, 29.2, 28.7, 28.62, 28.60, 26.7, 22.6, 21.1, 14.0.
*N-(tert-Butyl)-N-(2-(dodecylamino)-2-oxoethyl)acetamide* (**8c**) was obtained from acetic acid (0.50 mmol, 0.030 g), paraformaldehyde (0.50 mmol, 0.015 g), *t*-butyl amine (0.50 mmol, 0.052 g), and *n*-dodecyl isocyanide (0.50 mmol, 0.098 g), following the general procedure for Ugi reactions, in 69% yield (0.079 g) as a white solid after silica gel column chromatography (20% ethyl acetate/hexane → 60% ethyl acetate/hexane). R_f_ = 0.45 (60% ethyl acetate/hexane). m.p. 81–83 °C. ^1^H NMR (600 MHz, CDCl_3_) δ 6.14 (s, 1H), 3.96 (s, 2H), 3.30 (q, *J* = 7.0 Hz, 2H), 2.09 (s, 3H), 1.57–1.50 (m, 2H), 1.45 (s, 9H), 1.32–1.22 (m, 18H), 0.86 (t, *J* = 7.0 Hz, 3H). ^13^C NMR (151 MHz, CDCl_3_) δ 172.5, 169.6, 57.9, 50.3, 39.5, 31.9, 29.61, 29.56, 29.54, 29.50, 29.3, 29.2, 28.8, 26.9, 25.3, 22.7, 14.1.
*N-(2-(tert-Butylamino)-2-oxoethyl)-N-methylstearamide* (**8d**) was obtained from stearic acid (0.50 mmol, 0.142 g), paraformaldehyde (0.50 mmol, 0.015 g), methyl amine 2M in THF (0.50 mmol, 0.250 mL), and *t*-butyl isocyanide (0.50 mmol, 0.056 mL), following the general procedure for Ugi reactions, in 51% yield (0.103 g) as a white solid after silica gel column chromatography (100% hexane → 40% ethyl acetate/hexane). R_f_ = 0.20 (30% ethyl acetate/hexane). m.p. 73–74 °C. ^1^H NMR (600 MHz, CDCl_3_) δ 6.20 (s, 1H), 3.91 (s, 2H), 3.11 (s, 3H), 2.42–2.35 (m, 2H), 1.68–1.59 (m, 2H), 1.33 (s, 9H), 1.30–1.23 (m, 28H), 0.89 (t, *J* = 7.0 Hz, 3H). ^13^C NMR (151 MHz, CDCl_3_) δ 174.2, 168.5, 53.5, 36.9, 33.2, 31.9, 29.69, 29.67, 29.65, 29.62, 29.5, 29.43, 29.40, 29.36, 28.7, 25.1, 22.7, 14.1. HRMS (ESI-TOF) *m*/*z* calculated for C_25_H_50_N_2_O_2_ + H^+^: 411.3951 [M + H]^+^; found 411.3940.
*N-(tert-Butyl)-2-(N-octadecylacetamido)acetamide* (**8e**) was obtained from acetic acid (0.50 mmol, 0.030 mL), paraformaldehyde (0.50 mmol, 0.015 g), octadecyl amine (0.50 mmol, 0.134 g), and *t*-butyl isocyanide (0.50 mmol, 0.056 mL), following the general procedure for Ugi reactions, in 87% yield (0.185 g) as a white solid after silica gel column chromatography (30% ethyl acetate/hexane → 50% ethyl acetate/hexane). R_f_ = 0.40 (50% ethyl acetate). m.p. 66–68 °C. ^1^H NMR (600 MHz, CDCl_3_) δ 6.53 (s, 1H), 3.93–3.86 (m, 2H), 3.41–3.28 (t, 2H), 2.15 (s, 3H), 1.62–1.52 (q, 2H), 1.32 (s, 9H), 1.29–1.0 (m, 30H), 0.88 (t, *J* = 6.9 Hz, 3H). ^13^C NMR (151 MHz, CDCl_3_) δ 171.8, 169.0, 52.1, 51.3, 51.0, 31.9, 29.70, 29.68, 29.66, 29.64, 29.61, 29.5, 29.4, 29.30, 28.6, 26.8, 22.7, 21.2, 14.1.
*N-(tert-Butyl)-N-(2-(octadecylamino)-2-oxoethyl)acetamide* (**8f**) was obtained from acetic acid (0.50 mmol, 0.030 mL), paraformaldehyde (0.50 mmol, 0.015 g), *t*-butyl amine (0.50 mmol, 0.052 g), and octadecyl isocyanide (0.50 mmol, 0.084 g), following the general procedure for Ugi reactions, in 63% yield (0.080 g) as a white solid after silica gel column chromatography (30% ethyl acetate/hexane → 60% ethyl acetate/hexane). R_f_ = 0.20 (50% ethyl acetate/hexane). m.p. 84–85 °C. ^1^H NMR (600 MHz, CDCl_3_) δ 6.11 (s, 1H), 3.96 (s, 2H), 3.30 (q, *J* = 7.0 Hz, 2H), 2.09 (s, 3H), 1.57–1.46 (m, 2H), 1.45 (s, 9H), 1.32–1.22 (m, 30H), 0.88 (t, *J* = 7.0 Hz, 3H). ^13^C NMR (151 MHz, CDCl_3_) δ 172.5, 169.6, 57.9, 50.3, 39.5, 31.9, 29.71, 29.69, 29.67, 29.63, 29.59, 29.56, 29.52, 29.3, 29.2, 28.8, 26.9, 25.3, 22.7, 14.1. HRMS (ESI-TOF) *m*/*z* calculated for C_26_H_52_N_2_O_2_ + Na^+^: 447.3926 [M + H]^+^; found 447.3905.
*N-(2-(tert-Butylamino)-2-oxoethyl)-N-octylstearamide* (**8g**) was obtained from stearic acid (0.50 mmol, 0.142 g), paraformaldehyde (0.50 mmol, 0.015 g), *n*-octyl amine (0.50 mmol, 0.082 g), and *t*-butyl isocyanide (0.50 mmol, 0.056 mL), following the general procedure for Ugi reactions, in 90% yield (0.228 g) as a white solid after silica gel column chromatography (100% hexane → 20% ethyl acetate/hexane). R_f_ = 0.42 (20% ethyl acetate/hexane). m.p. 56–58 °C. ^1^H NMR (600 MHz, CDCl_3_) δ 6.60 (s, 1H), 3.94–3.84 (m, 2H), 3.42–3.31 (m, 2H), 2.41–2.33 (m, 2H), 1.73–1.53 (m, 4H), 1.33 (s, 9H), 1.30–1.20 (m, 28H), 0.89 (t, *J* = 7.0 Hz, 6H). ^13^C NMR (151 MHz, CDCl_3_) δ 174.3, 169.3, 52.5, 51.1, 50.1, 32.9, 31.9, 31.8, 29.71, 29.68, 29.67, 29.64, 29.54, 29.45, 29.43, 29.37, 29.26, 29.21, 28.8, 28.6, 26.8, 25.5, 22.7, 22.6, 14.12, 14.07. HRMS (ESI-TOF) *m*/*z* calculated for C_32_H_64_N_2_O_2_ + H^+^: 509.5046 [M + H]^+^; found 509.5043.
*N-(2-(tert-Butylamino)-2-oxoethyl)-N-dodecylstearamide* (**8h**) was obtained from stearic acid (0.50 mmol, 0.142 g), paraformaldehyde (0.50 mmol, 0.015 g), *n*-dodecyl amine (0.50 mmol, 0.092 g), and *t*-butyl isocyanide (0.50 mmol, 0.056 mL), following the general procedure for Ugi reactions, in 89% yield (0.151 g) as a white solid after silica gel column chromatography (100% hexane → 20% ethyl acetate/hexane). R_f_ = 0.40 (20% ethyl acetate/hexane). m.p. 55–57 °C. ^1^H NMR (600 MHz, CDCl_3_) δ 6.55 (s, 1H), 3.87 (s, 2H), 3.32–2.26 (m, 2H), 2.40–2.30 (m, 2H), 1.69–1.54 (m, 4H), 1.36–1.22 (m, 55H), 0.88 (t, *J* = 7.0 Hz, 6H). ^13^C NMR (151 MHz, CDCl_3_) δ 174.2, 169.2, 52.5, 51.0, 50.0, 32.9, 31.93, 31.9, 29.71, 29.69, 29.67, 29.64, 29.63, 29.61, 29.56, 29.55, 29.45, 29.43, 29.37, 29.34, 29.30, 28.8, 28.7, 28.6, 26.8, 25.5, 22.7, 14.1. HRMS (ESI-TOF) *m*/*z* calculated for C_36_H_72_N_2_O_2_ + H^+^: 565.5672 [M + H]^+^; found 565.5658.
*N-(2-(tert-Butylamino)-2-oxoethyl)-N-octadecylstearamide* (**8i**) was obtained from stearic acid (0.50 mmol, 0.142 g), paraformaldehyde (0.50 mmol, 0.015 g), octadecyl amine (0.50 mmol, 0.134 g), and *t*-butyl isocyanide (0.50 mmol, 0.056 mL), following the general procedure for Ugi reactions, in 95% yield (0.308 g) as a white solid after silica gel column chromatography (100% hexane → 20% ethyl acetate/hexane). R_f_ = 0.53 (20% ethyl acetate/hexane). m.p. 90–92 °C. ^1^H NMR (600 MHz, CDCl_3_) δ 4.01–3.87 (m, 2H), 3.42–3.32 (m, 2H), 2.41–2.31 (m, 2H), 1.70–1.57 (m, 4H), 1.33 (s, 9H), 1.32–1.22 (m, 58H), 0.90 (t, *J* = 7.0 Hz, 6H). ^13^C NMR (151 MHz, CDCl_3_) δ 174.3, 169.1, 52.3, 51.6, 50.2, 32.8, 31.9, 29.71, 29.69, 29.67, 29.64, 29.57, 29.56, 29.54, 29.45, 29.42, 29.37, 29.30, 28.8, 28.6, 26.8, 25.5, 22.7, 14.1. HRMS (ESI-TOF) *m*/*z* calculated for C_42_H_84_N_2_O_2_ + H^+^: 649.6611 [M + H]^+^; found 649.6592.
*N-(tert-Butyl)-N-(2-(octylamino)-2-oxoethyl)stearamide* (**8j**) was obtained from stearic acid (0.50 mmol, 0.142 g), paraformaldehyde (0.50 mmol, 0.015 g), *t*-buyl amine (0.50 mmol, 0.052 mL), and *n*-octyl isocyanide (0.50 mmol, 0.089 mL), following the general procedure for Ugi reactions, in 55% yield (0.138 g) as a white solid after silica gel column chromatography (100% hexane → 20% ethyl acetate/hexane). R_f_ = 0.33 (20% ethyl acetate/hexane). m.p. 50–52 °C. ^1^H NMR (600 MHz, CDCl_3_) δ 6.13 (s, 1H), 3.96 (s, 2H), 3.32 (q, *J* = 7.0 Hz, 2H), 2.23 (t, *J* = 7.0 Hz, 2H), 1.67–1.56 (m, 2H), 1.53–1.49 (m, 2H), 1.46 (s, 9H), 1.33–1.23 (m, 38H), 0.89 (t, *J* = 7.0 Hz, 6H). ^13^C NMR (151 MHz, CDCl_3_) δ 175.0, 169.9, 57.9, 49.4, 39.5, 36.3, 31.9, 31.7, 29.71, 29.68, 29.66, 29.65, 29.57, 29.54, 29.52, 29.4, 29.2, 28.8, 26.9, 25.4, 22.7, 22.6, 14.12, 14.07. HRMS (ESI-TOF) *m*/*z* calculated for C_32_H_64_N_2_O_2_ + H^+^: 509.5046 [M + H]^+^; found 509.5026.
*N-(tert-Butyl)-N-(2-(dodecylamino)-2-oxoethyl)stearamide* (**8k**) was obtained from stearic acid (0.50 mmol, 0.142 g), paraformaldehyde (0.50 mmol, 0.015 g), *t*-buyl amine (0.50 mmol, 0.052 mL), and *n*-dodecyl isocyanide (0.50 mmol, 0.058 g), following the general procedure for Ugi reactions, in 73% yield (0.127 g) as a white solid after silica gel column chromatography (100% hexane → 20% ethyl acetate/hexane). R_f_ = 0.32 (20% ethyl acetate/hexane). m.p. 60–62 °C. ^1^H NMR (600 MHz, CDCl_3_) δ 6.11 (s, 1H), 3.96 (s, 2H), 3.32 (q, *J* = 7.0 Hz, 2H), 2.23 (t, *J* = 7.0 Hz, 2H), 1.68–1.57 (m, 2H), 1.55–1.48 (m, 2H), 1.46 (s, 9H), 1.34–1.24 (m, 46H), 0.90 (t, *J* = 7.0 Hz, 6H). ^13^C NMR (151 MHz, CDCl_3_) δ 174.9, 169.9, 57.8, 49.3, 39.5, 36.3, 31.92, 31.9, 29.69, 29.68, 29.67, 29.65, 29.64, 29.63, 29.61, 29.57, 29.56, 29.53, 29.50, 29.36, 29.35, 29.33, 29.24, 28.8, 26.9, 25.4, 22.7, 14.1. HRMS (ESI-TOF) *m*/*z* calculated for C_36_H_72_N_2_O_2_ + Na^+^: 587.5491 [M + Na]^+^; found 587.5472.
*N-(tert-Butyl)-N-(2-(octadecylamino)-2-oxoethyl)stearamide* (**8l**) was obtained from stearic acid (0.50 mmol, 0.142 g), paraformaldehyde (0.50 mmol, 0.015 g), *t*-buyl amine (0.50 mmol, 0.052 mL), and octadecyl isocyanide (0.50 mmol, 0.084 g), following the general procedure for Ugi reactions, in 75% yield (0.146 g) as a white solid after silica gel column chromatography (100% hexane → 20% ethyl acetate/hexane). R_f_ = 0.62 (20% ethyl acetate/hexane). m.p. 85–87 °C. ^1^H NMR (600 MHz, CDCl_3_) δ 6.11 (s, 1H), 3.97 (s, 2H), 3.32 (q, *J* = 7.0 Hz, 2H), 2.23 (t, *J* = 7.0 Hz, 2H), 1.66–1.59 (m, 2H), 1.55–1.48 (m, 2H), 1.47 (s, 9H), 1.37–1.24 (m, 58H), 0.90 (t, *J* = 7.0 Hz, 6H). ^13^C NMR (151 MHz, CDCl_3_) δ 174.9, 169.9, 57.8, 49.4, 39.5, 36.3, 31.9, 29.68, 29.63, 29.59, 29.55, 29.52, 29.49, 29.37, 29.33, 29.23, 28.9, 26.9, 25.4, 22.6, 14.0. HRMS (ESI-TOF) *m*/*z* calculated for C_42_H_84_N_2_O_2_ + Na^+^: 671.6430 [M + Na]^+^; found 671.6422.
*N-Octyl-2-(N-octylacetamido)acetamide* (**8m**) was obtained from acetic acid (0.50 mmol, 0.030 mL), paraformaldehyde (0.50 mmol, 0.015 g), *n*-octyl amine (0.50 mmol, 0.082 mL), and *n*-octyl isocyanide (0.50 mmol, 0.089 mL), following the general procedure for Ugi reactions, in 64% yield (0.108 g) as a white solid after silica gel column chromatography (40% ethyl acetate/hexane → 70% ethyl acetate/hexane). R_f_ = 0.35 (50% ethyl acetate/hexane). m.p. 60–62 °C. ^1^H NMR (600 MHz, CDCl_3_) δ 6.70 (s, 1H), 4.02–3.91 (m, 2H), 3.40–3.31 (m, 2H), 3.24–3.14 (m, 2H), 2.16 (s, 3H), 1.66–1.57 (m, 2H), 1.51–1.42 (m, 2H), 1.33–1.21 (m, 20H), 0.89 (t, *J* = 7.0 6H). ^13^C NMR (151 MHz, CDCl_3_) δ 171.7, 169.6, 51.2, 50.8, 39.5, 31.8, 31.7, 29.4, 29.25, 29.21, 29.20, 28.6, 26.9, 26.7, 22.64, 22.60, 21.2, 14.08, 14.06. HRMS (ESI-TOF) *m*/*z* calculated for C_20_H_40_N_2_O_2_ + Na^+^: 363.2987 [M + Na]^+^; found 363.2979.
*N-Dodecyl-2-(N-dodecylacetamido)acetamide* (**8n**) was obtained from acetic acid (0.50 mmol, 0.030 mL), paraformaldehyde (0.50 mmol, 0.015 g), *n*-dodecyl amine (0.50 mmol, 0.055 g), and *n*-dodecyl isocyanide (0.50 mmol, 0.058 mL), following the general procedure for Ugi reactions, in 52% yield (0.070 g) as a white solid after silica gel column chromatography (30% ethyl acetate/hexane → 60% ethyl acetate/hexane). R_f_ = 0.28 (60% ethyl acetate/hexane). m.p. 77–78 °C. ^1^H NMR (600 MHz, CDCl_3_) δ 6.72 (s, 1H), 3.96 (s, 2H), 3.38–3.32 (m, 2H), 3.23–3.14 (m, 2H), 2.16 (s, 3H), 1.62–1.54 (m, 2H), 1.49–1.41 (m, 2H), 1.29–1.23 (m, 37H), 0.88 (t, *J* = 7.0 Hz, 6H). ^13^C NMR (151 MHz, CDCl_3_) δ 171.7, 169.6, 51.2, 50.9, 39.5, 31.9, 29.66, 29.64, 29.61, 29.60, 29.56, 29.55, 29.42, 29.35, 29.33, 29.30, 29.27, 28.6, 26.9, 26.7, 22.7, 21.2, 14.1. HRMS (ESI-TOF) *m*/*z* calculated for C_28_H_56_N_2_O_2_ + H^+^: 453.4420 [M + H]^+^; found 453.4407.
*N-Octadecyl-2-(N-octadecylacetamido)acetamide* (**8o**) was obtained from acetic acid (0.50 mmol, 0.030 mL), paraformaldehyde (0.50 mmol, 0.015 g), octadecyl amine (0.50 mmol, 0.134 g), and octadecyl isocyanide (0.50 mmol, 0.084 g), following the general procedure for Ugi reactions, in 64% yield (0.197 g) as a white solid, which was used without further purification. The product precipitated and was filtered under vacuum. m.p. 89–91 °C. ^1^H NMR (600 MHz, CDCl_3_) δ 6.61 (s, 1H), 3.95 (s, 2H), 3.42–3.32 (m, 2H), 3.24–3.17 (m, 2H), 2.17 (s, 3H), 1.67–1.57 (m, 2H), 1.49–1.42 (m, 2H), 1.32–1.23 (m, 60H), 0.90 (t, *J* = 7.0 Hz, 6H). ^13^C NMR (151 MHz, CDCl_3_) δ 171.5, 169.6, 51.3, 50.8, 39.4, 31.9, 29.70, 29.66, 29.62, 29.60, 29.56, 29.55, 29.52, 29.45, 29.36, 29.30, 29.27, 28.6, 26.9, 26.7, 22.7, 21.2, 14.1. HRMS (ESI-TOF) *m*/*z* calculated for C_40_H_80_N_2_O_2_ + H^+^: 621.6298 [M + H]^+^; found 621.6284.
*N-Dodecyl-N-(2-(octylamino)-2-oxoethyl)stearamide* (**8p**) was obtained from stearic acid (0.50 mmol, 0.142 g), paraformaldehyde (0.50 mmol, 0.015 g), dodecyl amine (0.50 mmol, 0.082 mL), and *n*-octyl isocyanide (0.50 mmol, 0.089 mL), following the general procedure for Ugi reactions, in 59% yield (0.181 g) as a white solid after silica gel column chromatography (100% hexane → 30% ethyl acetate/hexane). R_f_ = 0.42 (20% ethyl acetate/hexane) m.p. 66–68 °C ^1^H NMR (600 MHz, CDCl_3_) δ 6.69 (s, 1H), 3.94 (s, 2H), 3.39–3.29 (m, 2H), 3.22–3.14 (m, 2H), 2.39–2.31 (m, 2H), 1.72–1.40 (m, 6H), 1.35–1.22 (m, 56H), 0.94–0.82 (m, 9H). ^13^C NMR (151 MHz, CDCl_3_) δ 174.3, 169.9, 51.5, 50.0, 39.4, 32.9, 31.94, 31.92, 31.8, 29.71, 29.69, 29.67, 29.63, 29.56, 29.48, 29.46, 29.37, 29.35, 29.30, 29.25, 28.7, 26.9, 26.7, 25.4, 22.69, 22.65, 14.12, 14.09. HRMS (ESI-TOF) *m*/*z* calculated for C_40_H_80_N_2_O_2_ + H^+^: 621.6298 [M + H]^+^; found 621.6291.
*N-(2-(Dodecylamino)-2-oxoethyl)-N-octylstearamide* (**8q**) was obtained from stearic acid (0.50 mmol, 0.142 g), paraformaldehyde (0.50 mmol, 0.015 g), *n*-octyl amine (0.50 mmol, 0.082 mL), and dodecyl isocyanide (0.50 mmol, 0.098 g), following the general procedure for Ugi reactions, in 82% yield (0.254 g) as a white solid after silica gel column chromatography (100% hexane → 20% ethyl acetate/hexane). R_f_ = 0.24 (10% ethyl acetate/hexane) m.p. 64–66 °C. ^1^H NMR (600 MHz, CDCl_3_) δ 4.03–3.95 (m, 2H), 3.37–3.32 (m, 2H), 3.22–3.16 (m, 2H), 2.40–2.31 (m, 2H), 1.68–1.51 (m, 4H), 1.33–1.22 (m, 56H), 0.88 (t, *J* = 7.0, 9H). ^13^C NMR (151 MHz, CDCl_3_) δ 174.7, 170.0, 51.3, 50.1, 39.5, 32.9, 31.93, 31.7, 29.71, 29.69, 29.68, 29.67, 29.65, 29.63, 29.59, 29.55, 29.47, 29.37, 29.29, 29.25, 29.21, 28.7, 26.9, 26.7, 25.4, 22.7, 22.6, 14.12, 14.07. HRMS (ESI-TOF) *m*/*z* calculated for C_40_H_80_N_2_O_2_ + H^+^: 621.6298 [M + H]^+^; found 621.6283.
*N-Dodecyl-N-(2-(dodecylamino)-2-oxoethyl)stearamide* (**8r**) was obtained from stearic acid (0.50 mmol, 0.142 g), paraformaldehyde (0.50 mmol, 0.015 g), dodecyl amine (0.50 mmol, 0.092 g), and dodecyl isocyanide (0.50 mmol, 0.098 g), following the general procedure for Ugi reactions, in 75% yield (0.253 g) as a white solid after silica gel column chromatography (100% hexane → 30% ethyl acetate/hexane). R_f_ = 0.51 (30% ethyl acetate/hexane). m.p. 66–68 °C. ^1^H NMR (600 MHz, CDCl_3_) δ 6.93 (s, 1H), 3.99 (s, 2H), 3.39–3.30 (m, 2H), 3.24–3.14 (m, 2H), 2.39–2.31 (m, 2H), 1.68–1.44 (m, 6H), 1.32–1.21 (m, 64H), 0.88 (t, *J* = 7.0 Hz, 9H). ^13^C NMR (151 MHz, CDCl_3_) δ 174.6, 170.0, 51.4, 50.1, 39.5, 32.9, 31.93, 31.92, 29.72, 29.69, 29.67, 29.64, 29.63, 29.59, 29.57, 29.47, 29.39, 29.37, 29.36, 29.30, 28.8, 26.9, 26.7, 25.4, 22.7, 14.1. HRMS (ESI-TOF) *m*/*z* calculated for C_44_H_88_N_2_O_2_ + H^+^: 677.6924 [M + H]^+^; found 677.6916.
*N-Octadecyl-N-(2-(octadecylamino)-2-oxoethyl)stearamide* (**8s**) was obtained from stearic acid (0.50 mmol, 0.142 g), paraformaldehyde (0.50 mmol, 0.015 g), octadecyl amine (0.50 mmol, 0.134 g), and octadecyl isocyanide (0.50 mmol, 0.140 g), following the general procedure for Ugi reactions, in 87% yield (0.367 g) as a white solid, which was used without further purification. The product precipitated and was filtered under vacuum. m.p. 83–85 °C. ^1^H NMR (600 MHz, CDCl_3_) δ 6.69 (s, 1H), 3.96 (s, 1H), 3.51 (s, 1H), 3.41–3.32 (m, 2H), 3.28–3.16 (m, 2H), 2.41–2.30 (m, 2H), 1.70–1.55 (m, 4H), 1.51–1.41 (m, 2H), 1.40–1.19 (m, 88H), 0.90 (t, *J* = 7.0 Hz, 9H). ^13^C NMR (151 MHz, CDCl_3_) δ 174.3, 169.9, 51.5, 50.0, 39.4, 32.9, 31.9, 29.71, 29.69, 29.68, 29.66, 29.64, 29.61, 29.60, 29.57, 29.55, 29.46, 29.36, 29.30, 29.28, 29.1, 28.7, 26.9, 26.7, 25.4, 24.8, 22.7, 14.1.
*N-Octyl-N-(2-oxo-2-(phenylamino)ethyl)stearamide* (**8t**) was obtained from stearic acid (0.50 mmol, 0.142 g), paraformaldehyde (0.50 mmol, 0.015 g), *n*-octyl amine (0.50 mmol, 0.082 mL), and pheyl isocyanide (0.50 mmol, 0.051 g), following the general procedure for Ugi reactions, in 85% yield (0.224 g) as a pale yellow oil after silica gel column chromatography (100% hexane → 20% ethyl acetate/hexane). R_f_ = 0.57 (20% ethyl acetate/hexane). ^1^H NMR (600 MHz, CDCl_3_) δ 8.98 (s, 1H), 7.51 (d, *J* = 7.0 Hz, 2H), 7.31 (t, *J* = 7.0 Hz, 2H), 7.10 (t, *J* = 7.0 Hz, 1H), 4.11 (s, 2H), 3.46–3.37 (m, 2H), 2.42–2.34 (m, 2H), 1.73–1.58 (m, 4H), 1.35–1.23 (m, 38H), 0.90 (t, *J* = 7.0, 6H). ^13^C NMR (151 MHz, CDCl_3_) δ 175.0, 168.1, 138.0, 128.9, 124.1, 119.8, 61.9, 53.0, 50.3, 32.9, 31.9, 31.7, 29.70, 29.68, 29.65, 29.62, 29.52, 29.44, 29.42, 29.35, 29.22, 29.16, 28.7, 26.7, 25.4, 22.7, 22.6, 14.1, 14.0. HRMS (ESI-TOF) *m*/*z* calculated for C_34_H_60_N_2_O_2_ + Na^+^: 551.4552 [M + Na]^+^; found 551.4545.
*Methyl N-octyl-N-stearoylglycylglycinate* (**8u**) was obtained from stearic acid (0.50 mmol, 0.142 g), paraformaldehyde (0.50 mmol, 0.015 g), *n*-octyl amine (0.50 mmol, 0.082 mL), and methyl isocyanoacetate (0.50 mmol, 0.045 mL), following the general procedure for Ugi reactions, in 65% yield (0.171 g) as a white solid after silica gel column chromatography (20% ethyl acetate/hexane → 50% ethyl acetate/hexane). R_f_ = 0.40 (50% ethyl acetate/hexane). m.p. 54–56 °C. ^1^H NMR (600 MHz, CDCl_3_) δ 7.08 (s, 1H), 4.04–3.99 (m, 4H), 3.75 (s, 3H), 3.40–3.35 (m, 2H), 2.44–2.35 (m, 2H), 1.71–1.55 (m, 4H), 1.33–1.23 (m, 36H), 0.90 (t, *J* = 7.0, 6H). ^13^C NMR (151 MHz, CDCl_3_) δ 174.6, 170.02, 169.98, 52.2, 50.8, 49.9, 41.0, 32.9, 31.9, 31.7, 29.69, 29.67, 29.64, 29.63, 29.51, 29.43, 29.34, 29.22, 29.17, 28.7, 26.7, 25.3, 22.7, 22.6, 14.1, 14.0. HRMS (ESI-TOF) *m*/*z* calculated for C_31_H_60_N_2_O_4_ + Na^+^: 547.4451 [M + Na]^+^; found 547.4435.
*N-Octyl-N-stearoylglycylglycine* (**8v**): A Biotage microwave reaction vial of 0.5–2.0 mL containing a solution of peptoid **8u** (0.10 mmol, 0.052 g) in THF/H_2_O (1:1, 0.6 mL) and LiOH (0.50 mmol, 0.012 g) was introduced in the cavity of a microwave reactor (Biotage® Initiator^+^) and irradiated at 60 °C for 10 min under magnetic stirring. The reaction mixture was then acidified with a 2 M solution of NaHSO_4_ to pH 2 and extracted twice with ethyl acetate (3 × 10 mL). The organic phase was dried over sodium sulfate, filtered, and then concentrated to yield acid 8v (0.045 g) as a white solid in 88%, which was used without further purification. m.p. 72–74 °C ^1^H NMR (600 MHz, CDCl_3_) δ 7.12 (s, 1H), 4.12–3.97 (m, 4H), 3.40 (s, 2H), 2.45–2.35 (m, 2H), 1.69–1.57 (m, 4H), 1.36–1.25 (m, 38H), 0.90 (t, *J* = 7.0, 6H). ^13^C NMR (151 MHz, CDCl_3_) δ 175.2, 172.0, 169.7, 50.6, 50.0, 41.3, 32.9, 31.9, 31.7, 29.72, 29.70, 29.67, 29.55, 29.40, 29.37, 29.24, 29.20, 28.6, 26.7, 25.3, 22.7, 22.6, 14.12, 14.08.
*N-(1-(4-Hydroxyphenyl)-2-(octylamino)-2-oxoethyl)-N-octylstearamide* (**11a**) was obtained from stearic acid (0.50 mmol, 0.142 g), *p*-OH-benzaldehyde (0.50 mmol, 0.061 g), *n*-octyl amine (0.50 mmol, 0.082 mL), and *n*-octyl isocyanide (0.50 mmol, 0.089 mL), following the general procedure for Ugi reactions, in 47% yield (0.153 g) as a pale yellow oil. It was necessary to carry out two purifications because of the remaining *p*-OH-benzaldehyde. After the silica gel column chromatography (100% hexane → 30% ethyl acetate/hexane), the isolated oil was dissolved in dichloromethane and extracted twice with 1 M NaOH (2 × 10 mL). The organic phase was dried over sodium sulfate, filtered, and then concentrated. R_f_ = 0.55 (20% ethyl acetate/hexane). ^1^H NMR (600 MHz, CDCl_3_) δ 7.20 (d, *J* = 8.0 Hz, 2H), 6.80 (d, *J* = 8.0 Hz, 2H), 5.71 (s, 1H), 3.35–3.19 (m, 4H), 2.44–2.33 (m, 2H), 1.73–1.62 (m, 2H), 1.50–1.35 (m, 4H), 1.36–1.12 (m, 48H), 0.91–0.86 (m, 9H). ^13^C NMR (151 MHz, CDCl_3_) δ 174.6, 170.6, 156.9, 130.6, 126.3, 115.8, 62.7, 47.4, 39.8, 33.6, 31.9, 31.8, 31.7, 29.73, 29.71, 29.69, 29.67, 29.58, 29.52, 29.47, 29.37, 29.33, 29.21, 29.13, 28.95, 26.90, 26.87, 25.5, 22.70, 22.64, 22.61, 14.12, 14.09, 14.08. HRMS (ESI-TOF) *m*/*z* calculated for C_42_H_76_N_2_O_2_ + Na^+^: 679.5754 [M + H]^+^; found 679.5725.
*N-Octyl-N-(1-(octylamino)-1-oxooctan-2-yl)stearamide* (**11b**) was obtained from stearic acid (0.50 mmol, 0.142 g), *n*-heptaldehyde (0.50 mmol, 0.070 mL), *n*-octyl amine (0.50 mmol, 0.082 mL), and *n*-octyl isocyanide (0.50 mmol, 0.089 mL), following the general procedure for Ugi reactions, in 58% yield (0.086 g) as a pale yellow oil after silica gel column chromatography (100% hexane → 20% ethyl acetate/hexane). R_f_ = 0.50 (10% ethyl acetate/hexane). ^1^H NMR (600 MHz, CDCl_3_) δ 6.69 (s, 1H), 4.73 (s, 1H), 3.34–3.08 (m, 4H), 2.40–2.29 (m, 2H), 1.78–1.42 (m, 8H), 1.35–1.20 (m, 56H), 0.94–0.83 (m, 12H). ^13^C NMR (151 MHz, CDCl_3_) δ 174.8, 171.6, 58.0, 45.2, 39.2, 33.6, 31.9, 31.82, 31.76, 31.67, 30.1, 29.71, 29.69, 29.66, 29.56, 29.49, 29.37, 29.24, 29.14, 29.10, 28.1, 27.1, 26.9, 26.2, 25.6, 22.69, 22.65, 22.63, 22.56, 14.12, 14.08, 14.07, 14.04. HRMS (ESI-TOF) *m*/*z* calculated for C_42_H_84_N_2_O_2_ + H^+^: 649.6611 [M + H]^+^; found 649.6607.
*N-(1-(Octylamino)-1-oxooctan-2-yl)stearamide* (**11c**) was obtained from stearic acid (0.50 mmol, 0.142 g), *n*-heptaldehyde (0.50 mmol, 0.070 mL), ammonia solution 7 M in methanol (0.50 mmol, 0.070 mL), and *n*-octyl isocyanide (0.50 mmol, 0.089 mL), following the general procedure for Ugi reactions, in 40% yield (0.107 g) as a colorless oil after silica gel column chromatography (100% hexane → 30% ethyl acetate/hexane). R_f_ = 0.44 (20% ethyl acetate/hexane). ^1^H NMR (600 MHz, CDCl3) δ 4.50 (s, 1H), 3.37–3.17 (m, 2H), 2.32–2.20 (m, 2H), 1.72–1.41 (m, 8H), 1.33–1.24 (m, 44H), 0.95–0.85 (m, 9H). ^13^C NMR (151 MHz, CDCl3) δ 177.4, 172.1, 53.3, 39.8, 36.6, 33.83, 32.4, 31.94, 31.8, 31.6, 29.7, 29.69, 29.67, 29.65, 29.62, 29.5, 29.4, 29.3, 29.29, 29.26, 29.24, 29.22, 29.1, 29.03, 26.9, 25.7, 25.6, 24.8, 22.7, 22.6, 22.5, 14.1, 14.09, 14.04. HRMS (ESI-TOF) *m*/*z* calculated for C_34_H_68_N_2_O_2_ + H^+^: 537.5359 [M + H]^+^; found 537.5352.

#### 3.2.2. General Procedure for Nanoparticle Preparation

The corresponding Ugi product (0.050 g) was dissolved in acetone (13 mL) at 40 °C. This organic phase was added dropwise into 26 mL of a phosphate-buffered saline (PBS, pH = 7.4) phase containing polysorbate 80 (0.038 g) under magnetic stirring at room temperature. The mixture was stirred for 16 h to guarantee the complete evaporation of acetone. Formulations of nanoparticles showed a homogeneous white-bluish opalescent aspect, except for **8v** (colorless solution). The formulations were analyzed via DLS in the same phosphate-buffered medium. 

## 4. Conclusions

In summary, a diversity of lipid–peptoids were synthesized via varying the position (R_1_, R_2_, and R_3_), the size (C_8_, C_12_, and C_18_), and the number of long chains in the Ugi reaction. In total, 28 lipid–peptoids were obtained in good to excellent yields. All products were submitted to nanoparticle formation via the emulsification–evaporation process from lipophilic solution and analyzed via DLS. Several molecules led to the development of nanoparticles with a size ≤ 200 nm, which make them good candidates for drug delivery systems since in nanomedical application the preferential size is less than 200 nm. Compound **5c** showed the best nanoparticle size distribution (below 100 nm) in combination with a homogeneous population, and TEM images showed the presence of spherical-shaped nanoparticles with an average size of 91.608 nm. Regarding the structure of the lipid–peptoids, the use of carboxylic acids with long chains is essential for nanoparticle preparation. However, only one long chain in the structure is not sufficient to prepare nanoparticles, and the best results obtained so far were from molecules with two and three long chains (with the combination of carboxylic acid and amine or carboxylic acid and isocyanide). Further studies are being carried out to investigate the use of these NPs as drug carriers in cancer treatment.

## Figures and Tables

**Table 1:** Summary of the synthesized compounds and their average particle size measured via DLS and yields.

Entry	Peptoids	Average Particle Size (nm)	PDI	Yield (%)
1	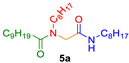	1300	1.000	74
2	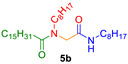	250	0.300	71
3	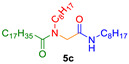	130	0.470	76
4	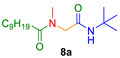	2000	0.700	55
5	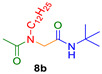	2500	0.170	88
6	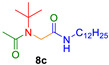	3500	1.000	69
7	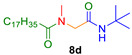	4100	0.180	51
8	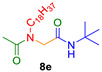	2000	1.000	87
9	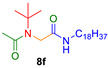	1200	1.000	63
10	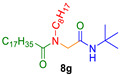	250	0.200	94
11	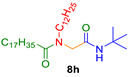	200	0.300	89
12	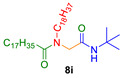	250	0.150	95
13	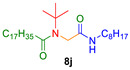	200	0.300	55
14	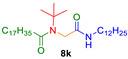	220	0.150	73
15	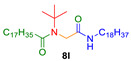	250	0.380	75
16	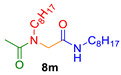	1700	0.350	64
17	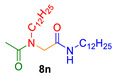	4000	1.000	52
18	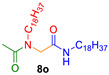	500	0.350	64
19	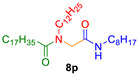	130	0.480	59
20	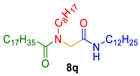	170	0.420	82
21	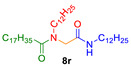	260	0.400	75
22	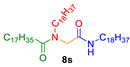	600	0.270	87
23	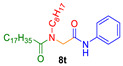	120	0.450	85
24	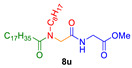	160	0.200	65
25	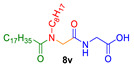	120	0.390	88
26	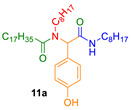	180	0.380	47
27	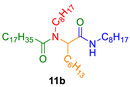	240	0.520	58
28	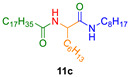	200	0.380	40

## Data Availability

Not applicable.

## Supplementary Materials

The following supporting information can be downloaded at: https://www.mdpi.com/article/10.3390/molecules28155725/s1. The supplementary material contains ^1^H, ^13^C NMR, and mass spectra for compounds **5a**–**c**, **8a**–**v**, and **11a**–**c**.
