# Supplementary material for: Rational Design and Multicomponent Synthesis of Lipid–Peptoid Nanocomposites towards a Customized Drug Delivery System Assembly
Source: Molecules. 2023 Jul 28;28(15):5725. doi: 10.3390/molecules28155725 (PMC10421149; doi:10.3390/molecules28155725)
Supplement: Supplementary file 1 [file molecules-28-05725-s001.zip › molecules-2518115-supplementary.pdf]

SUPPLEMENTARY INFORMATION

Rational design and multicomponent synthesis of lipid peptoid nanocomposites towards a customized drug delivery system assembly

Table of Contents

1. ^{1}H, ^{13}C NMR and mass spectra for compound **5a** ....pages S3-S5
2. ^{1}H, ^{13}C NMR and mass spectra for compound **5b** ....pages S6-S8
3. ^{1}H, ^{13}C NMR and mass spectra for compound **5c** ....pages S9-S11
4. ^{1}H, ^{13}C NMR and mass spectra for compound **8a** ....pages S12-S14
5. ^{1}H and ^{13}C NMR spectra for compound **8b** …………..................................pages S15-S16
6. ^{1}H and ^{13}C NMR spectra for compound **8c** …………..................................pages S17-S18
7. ^{1}H, ^{13}C NMR and mass spectra for compound **8d** ....pages S19-S21
8. ^{1}H and ^{13}C NMR spectra for compound **8e** ....pages S22-S23
9. ^{1}H, ^{13}C NMR and mass spectra for compound **8f** ....pages S24-S26
10. ^{1}H, ^{13}C NMR and mass spectra for compound **8g** ....pages S27-S29
11. ^{1}H, ^{13}C NMR and mass spectra for compound **8h** ....pages S30-S32
12. ^{1}H, ^{13}C NMR and mass spectra for compound **8i** ....pages S33-S35
13. ^{1}H, ^{13}C NMR and mass spectra for compound **8j** ....pages S36-S38
14. ^{1}H, ^{13}C NMR and mass spectra for compound **8k** ....pages S39-S41
15. ^{1}H, ^{13}C NMR and mass spectra for compound **8l** ....pages S42-S44
16. ^{1}H, ^{13}C NMR and mass spectra for compound **8m** ....pages S45-S47
17. ^{1}H, ^{13}C NMR and mass spectra for compound **8n** ....pages S48-S50
18. ^{1}H, ^{13}C NMR and mass spectra for compound **8o** ....pages S51-S53
19. ^{1}H, ^{13}C NMR and mass spectra for compound **8p** ....pages S54-S56
20. ^{1}H, ^{13}C NMR and mass spectra for compound **8q** ....pages S57-S59
21. ^{1}H, ^{13}C NMR and mass spectra for compound **8r** ....pages S60-S62
22. ^{1}H and ^{13}C NMR spectra and for compound **8s** ………..................................pages S63-S64
23. ^{1}H, ^{13}C NMR and mass spectra for compound **8t** ....pages S65-S67
24. ^{1}H, ^{13}C NMR and mass spectra for compound **8u** ....pages S68-S70
25. ^{1}H and ^{13}C NMR spectra for compound **8v** ....pages S71-S72
26. ^{1}H, ^{13}C NMR and mass spectra for compound **11a** ....pages S73-S75

27. ^{1}H, ^{13}C NMR and mass spectra for compound **11b** ....pages S76-S78
28. ^{1}H, ^{13}C NMR and mass spectra for compound **11c** ....pages S79-S81

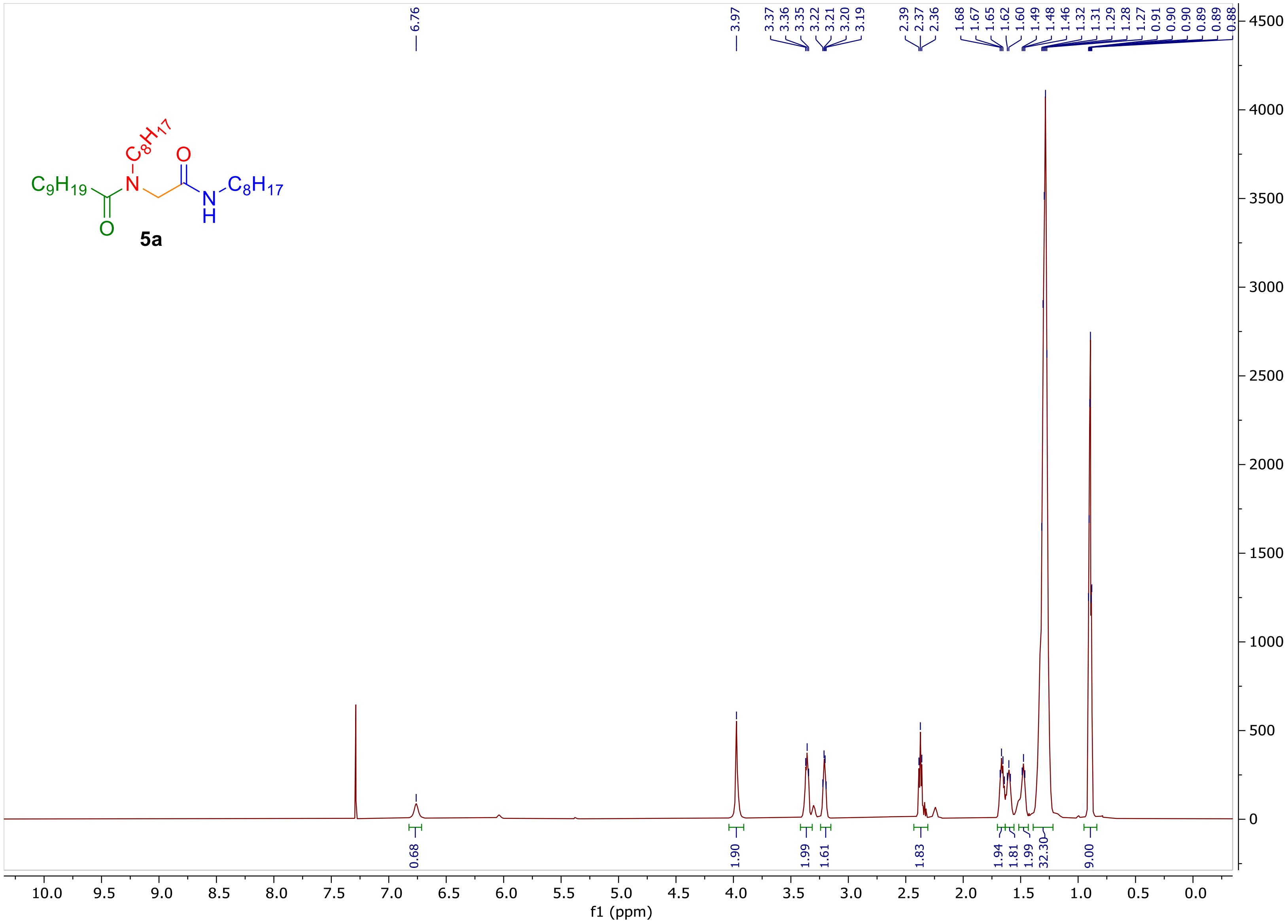

Figure S1. ^{1}H NMR (600 MHz, $CDCl_3$) Spectrum of compound **5a**.

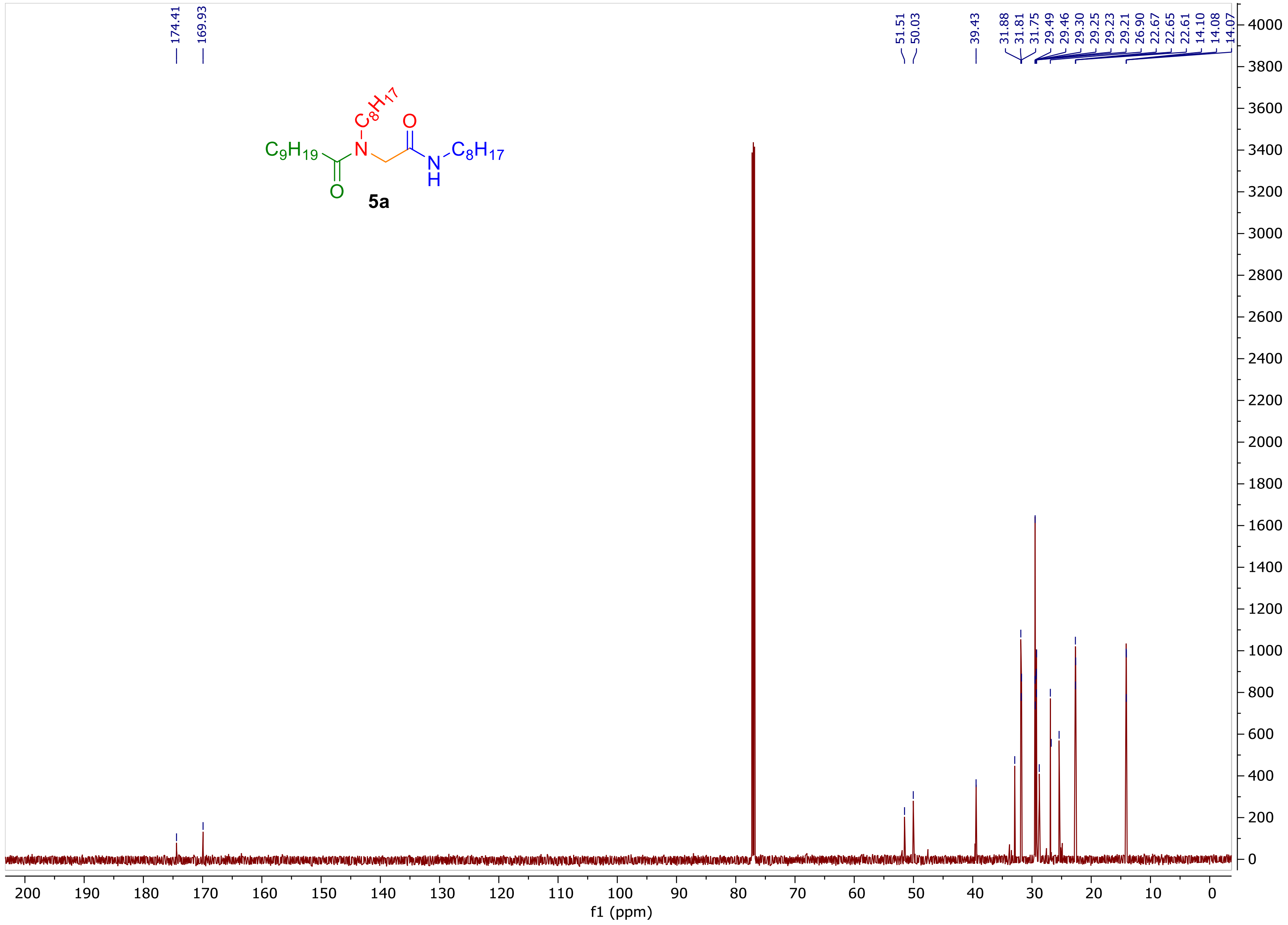

Figure S2. ^{13}C NMR (151 MHz, $CDCl_3$) Spectrum of compound **5a**.

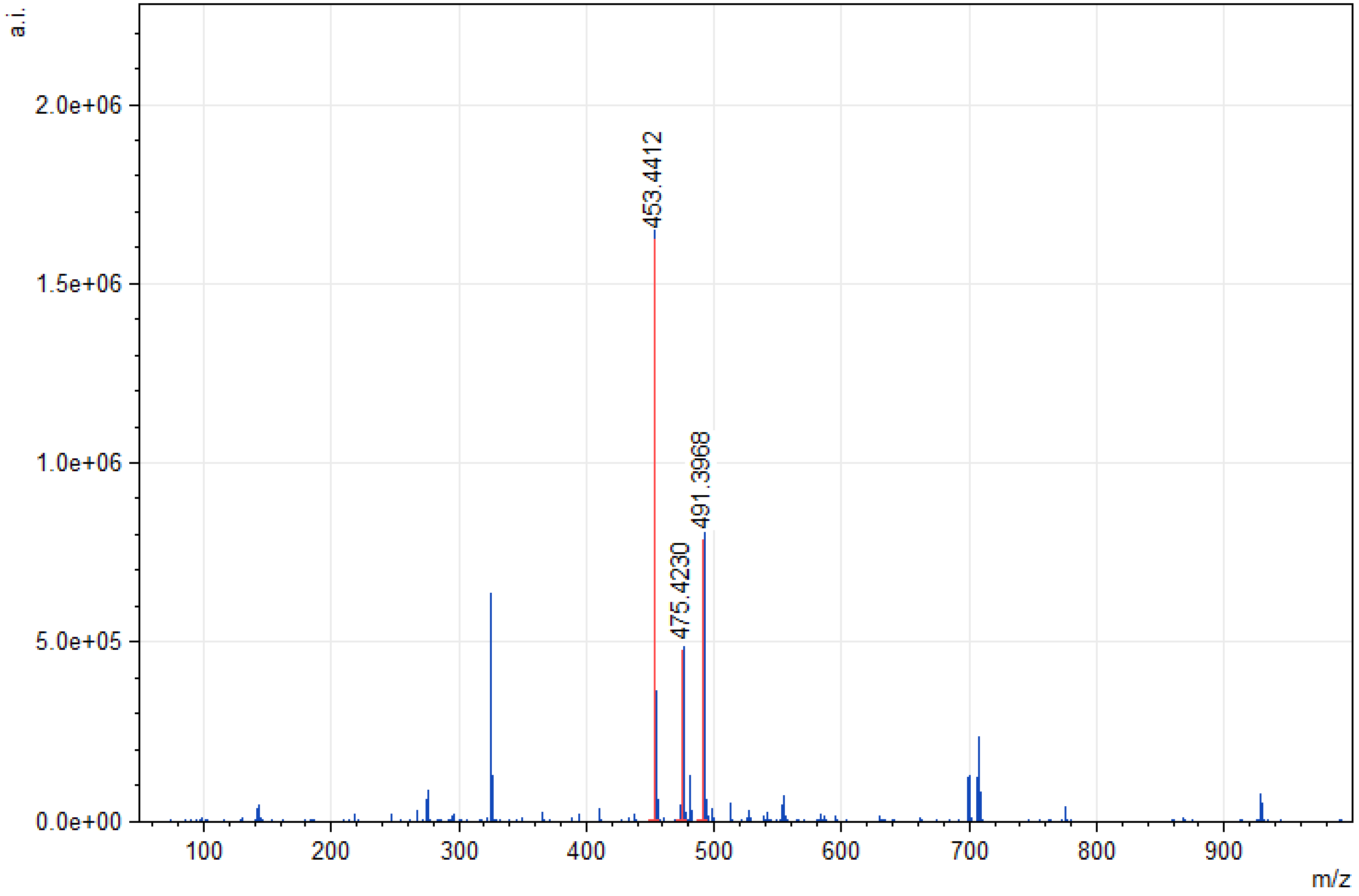

Figure S3. HRMS of compound **5a**.

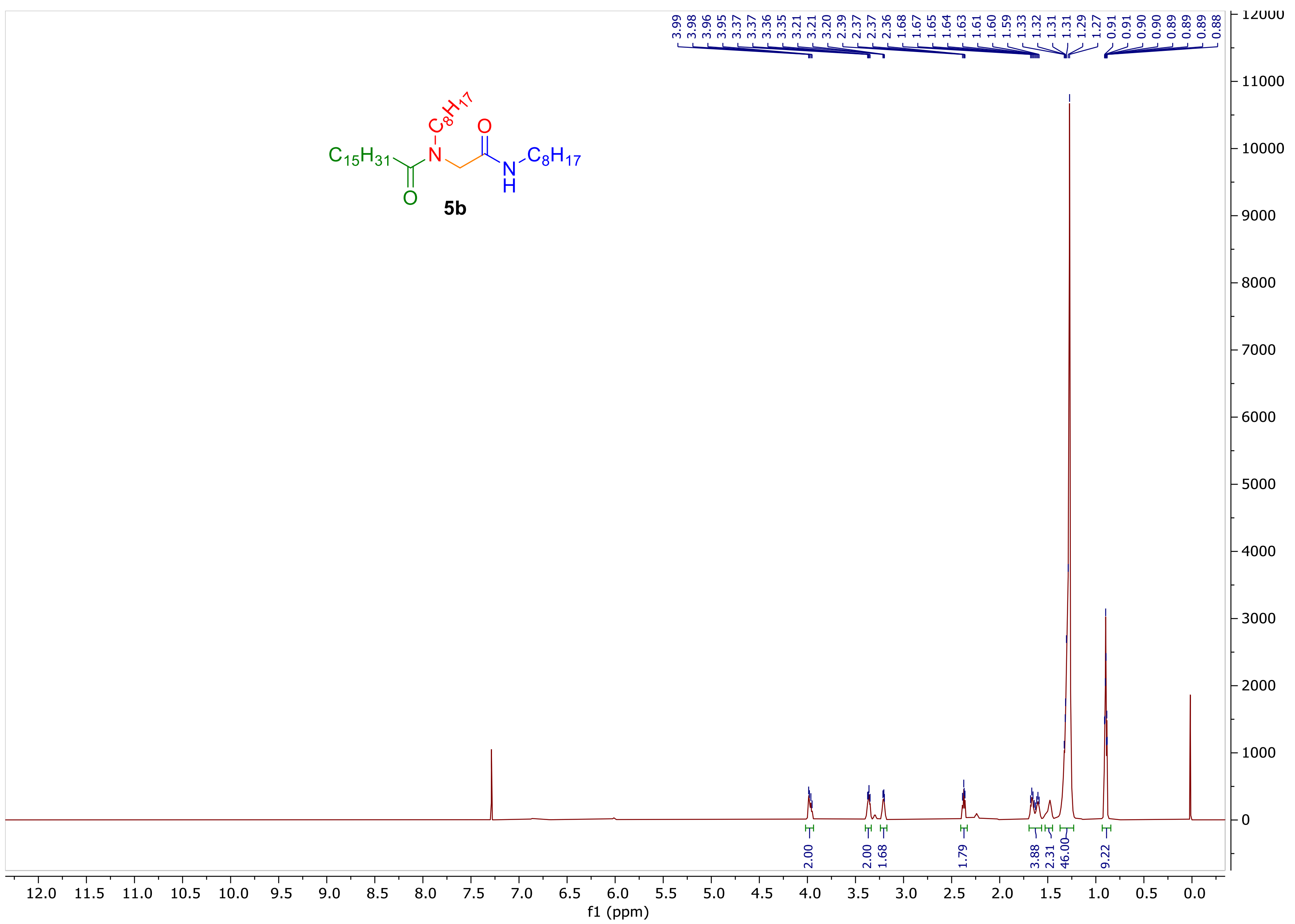

Figure S4. ^{1}H NMR (600 MHz, $CDCl_3$) Spectrum of compound **5b**.

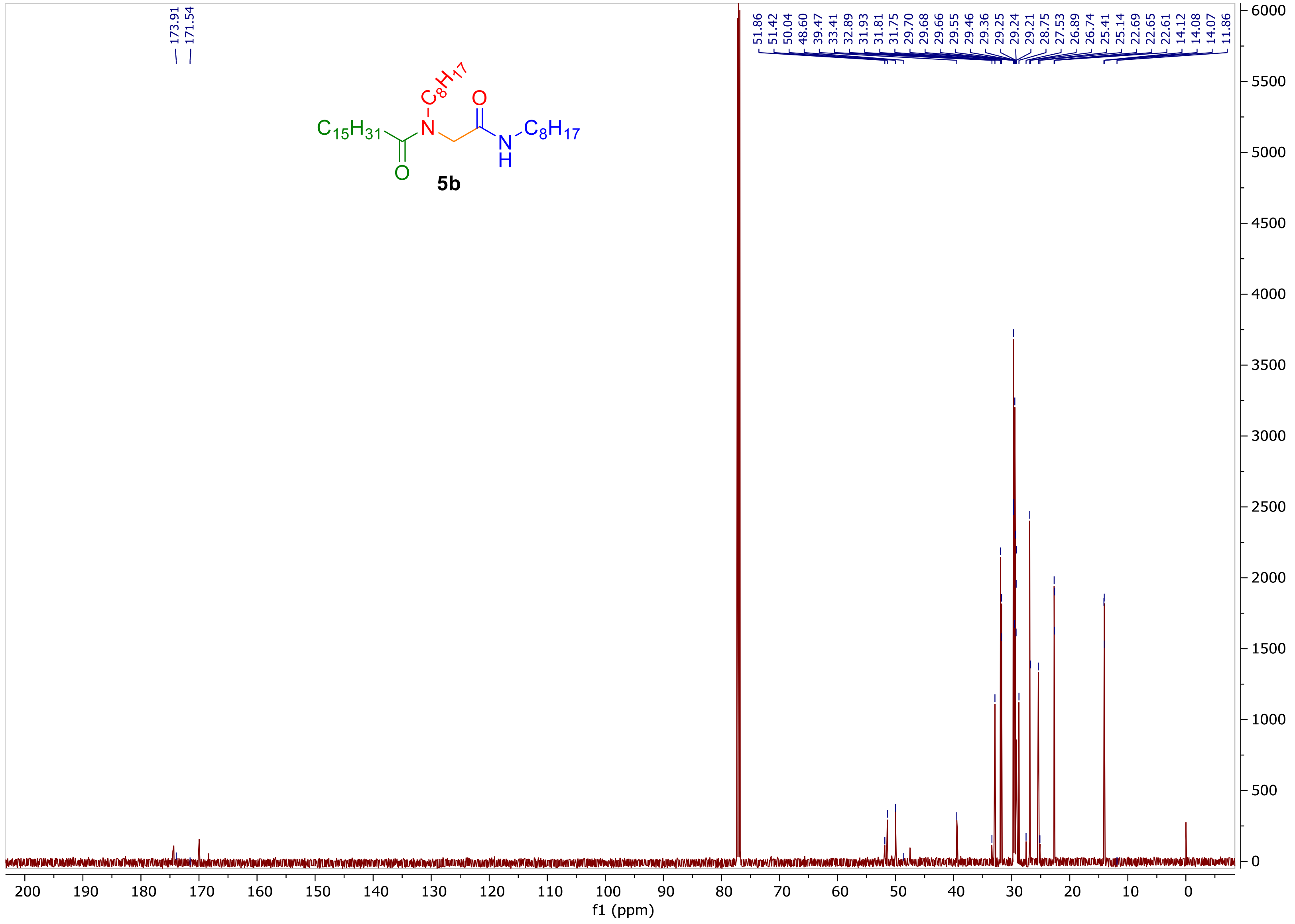

Figure S5. ^{13}C NMR (151 MHz, $CDCl_3$) Spectrum of compound **5b**.

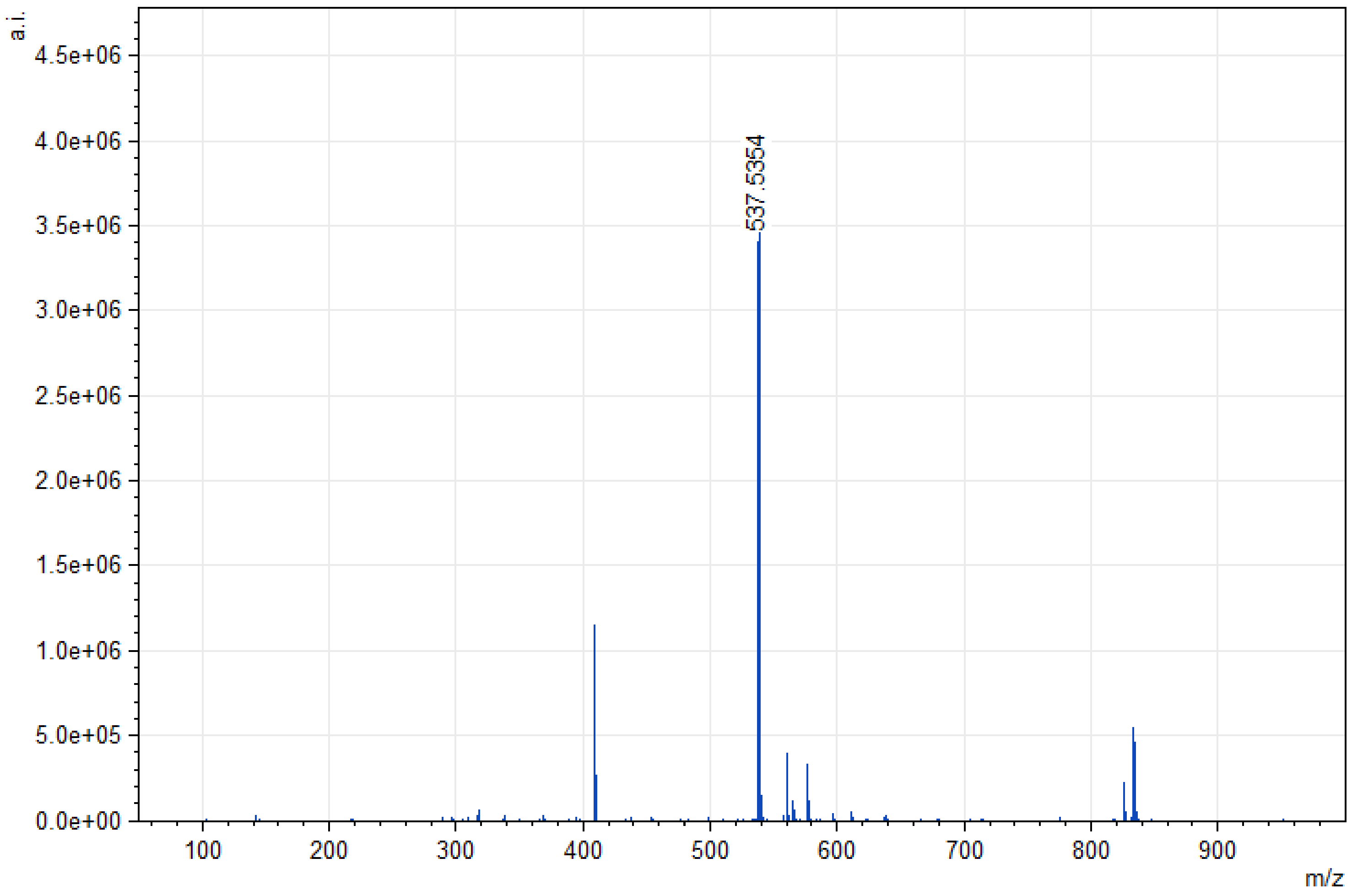

Figure S6. HRMS of compound **5b**.

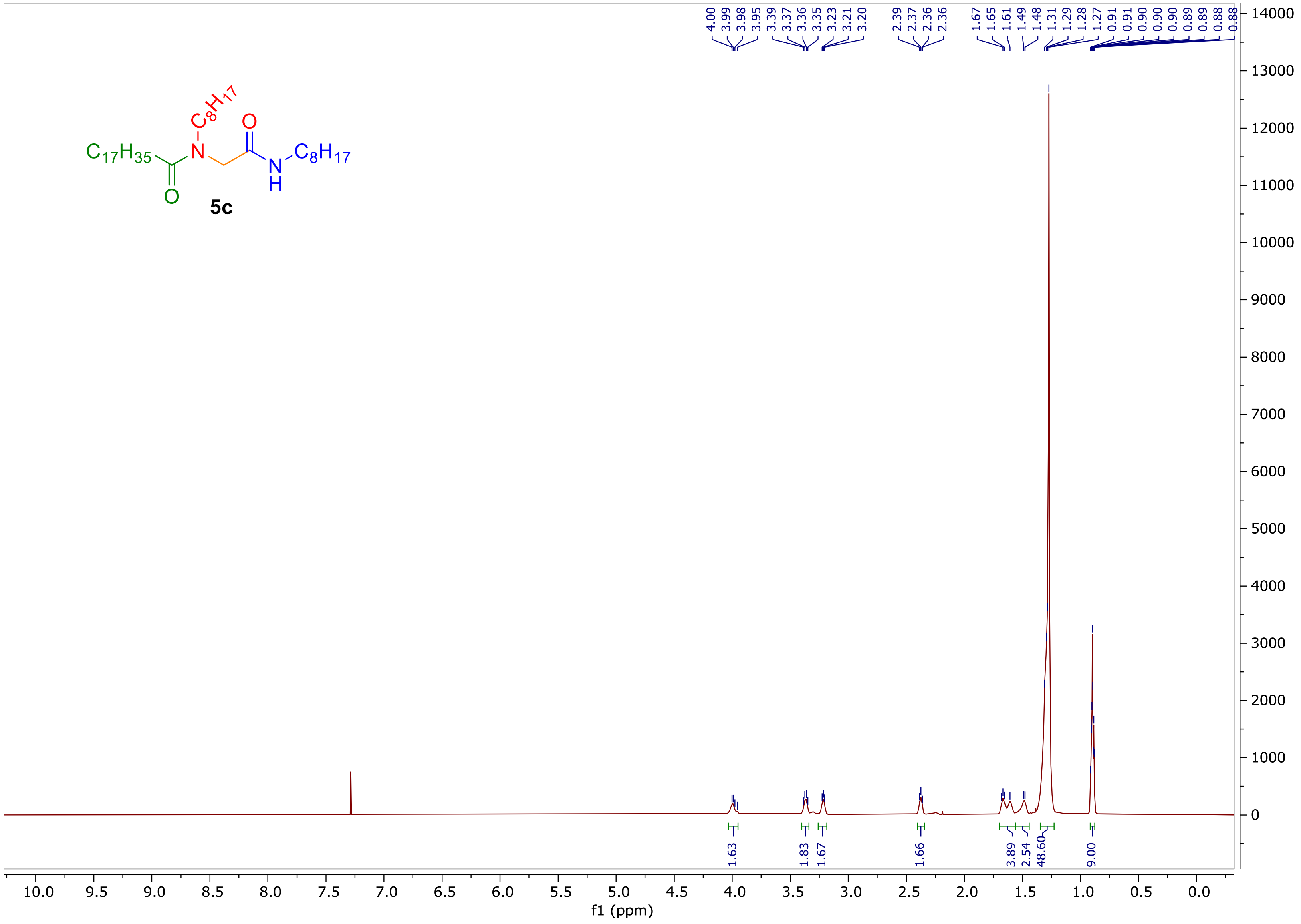

Figure S7. ^{1}H NMR (600 MHz, $CDCl_3$) Spectrum of compound **5c**.

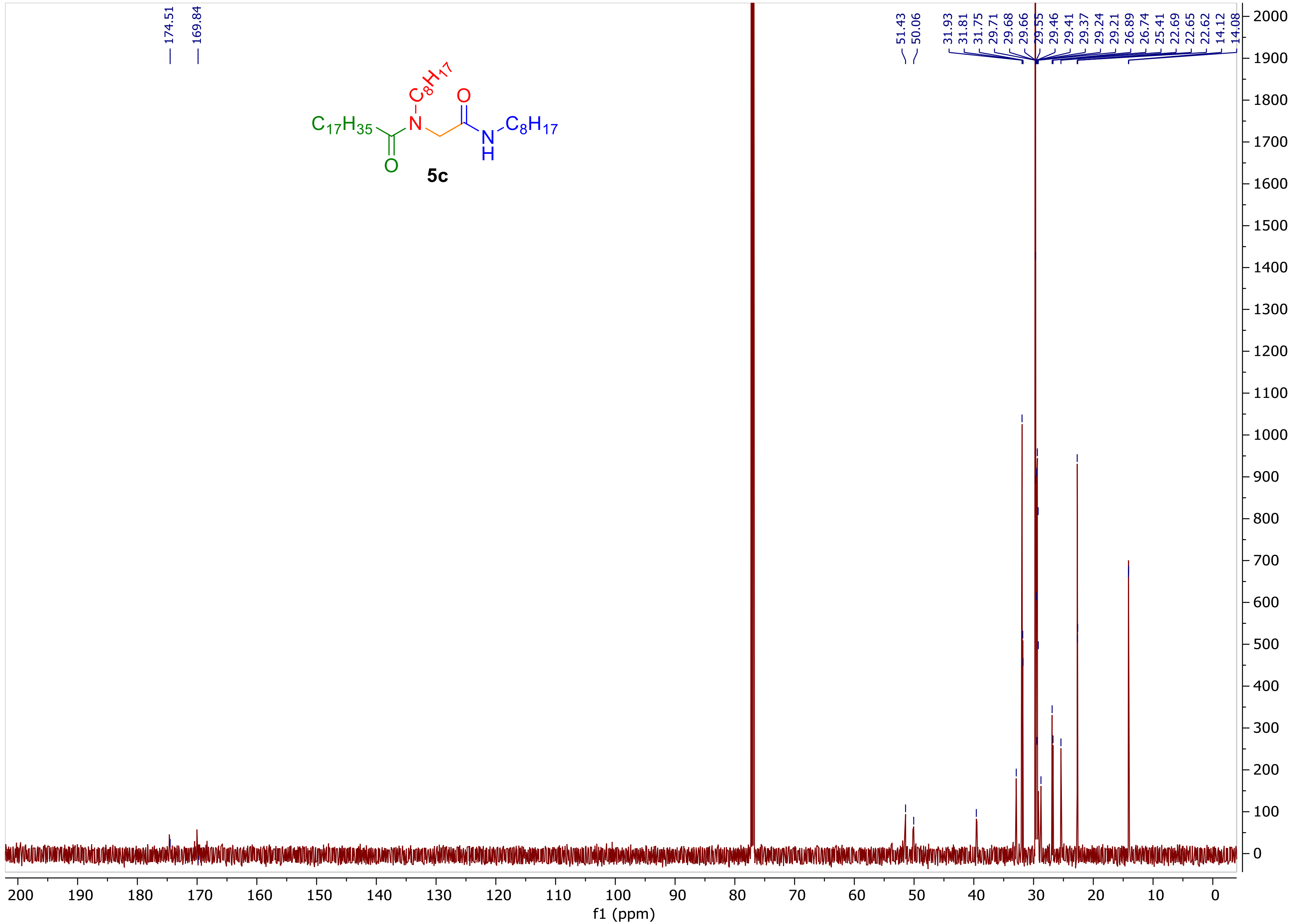

Figure S8. ^{13}C NMR (151 MHz, $CDCl_3$) Spectrum of compound **5c**.

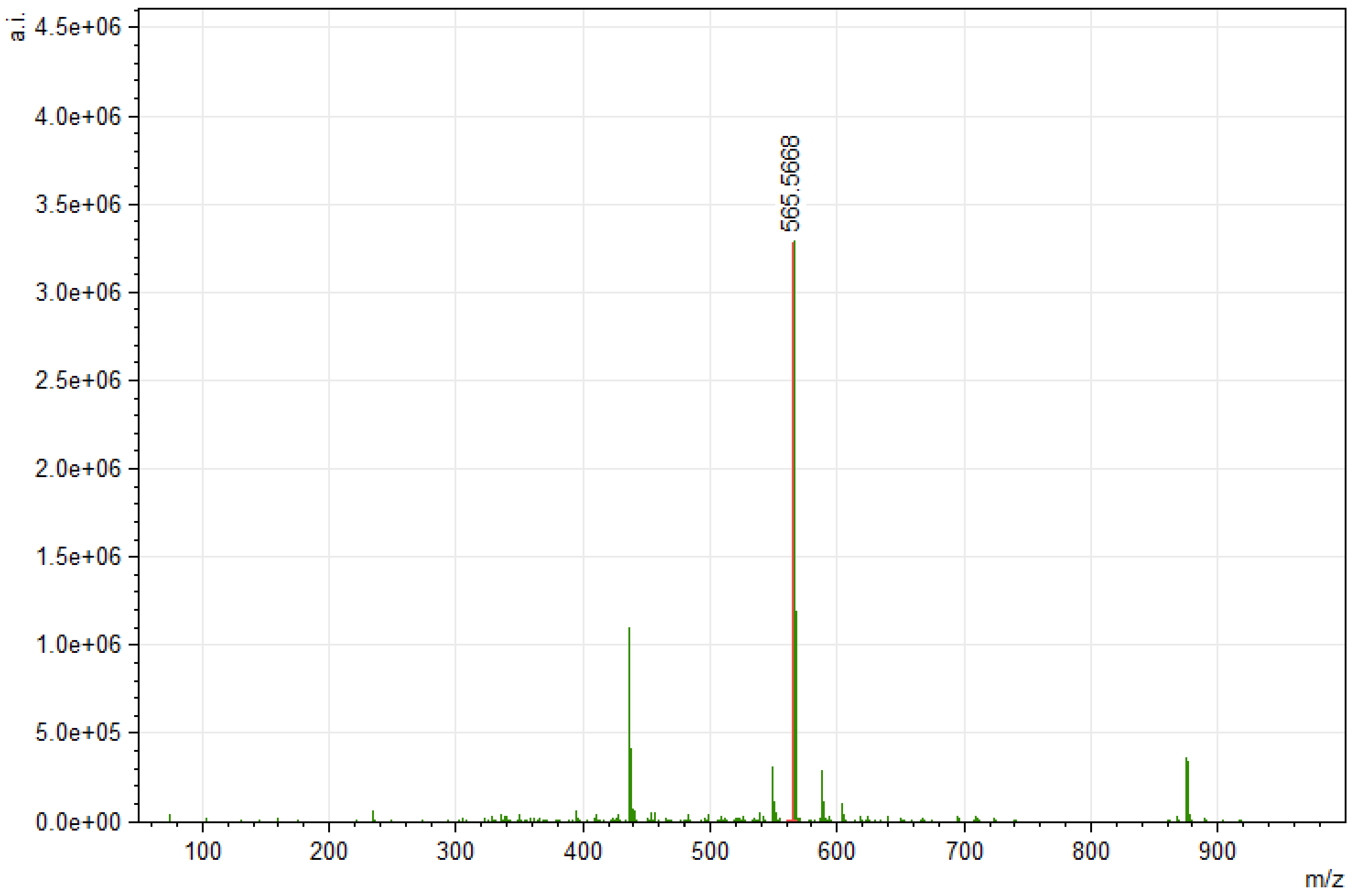

Figure S9. HRMS of compound **5c**.

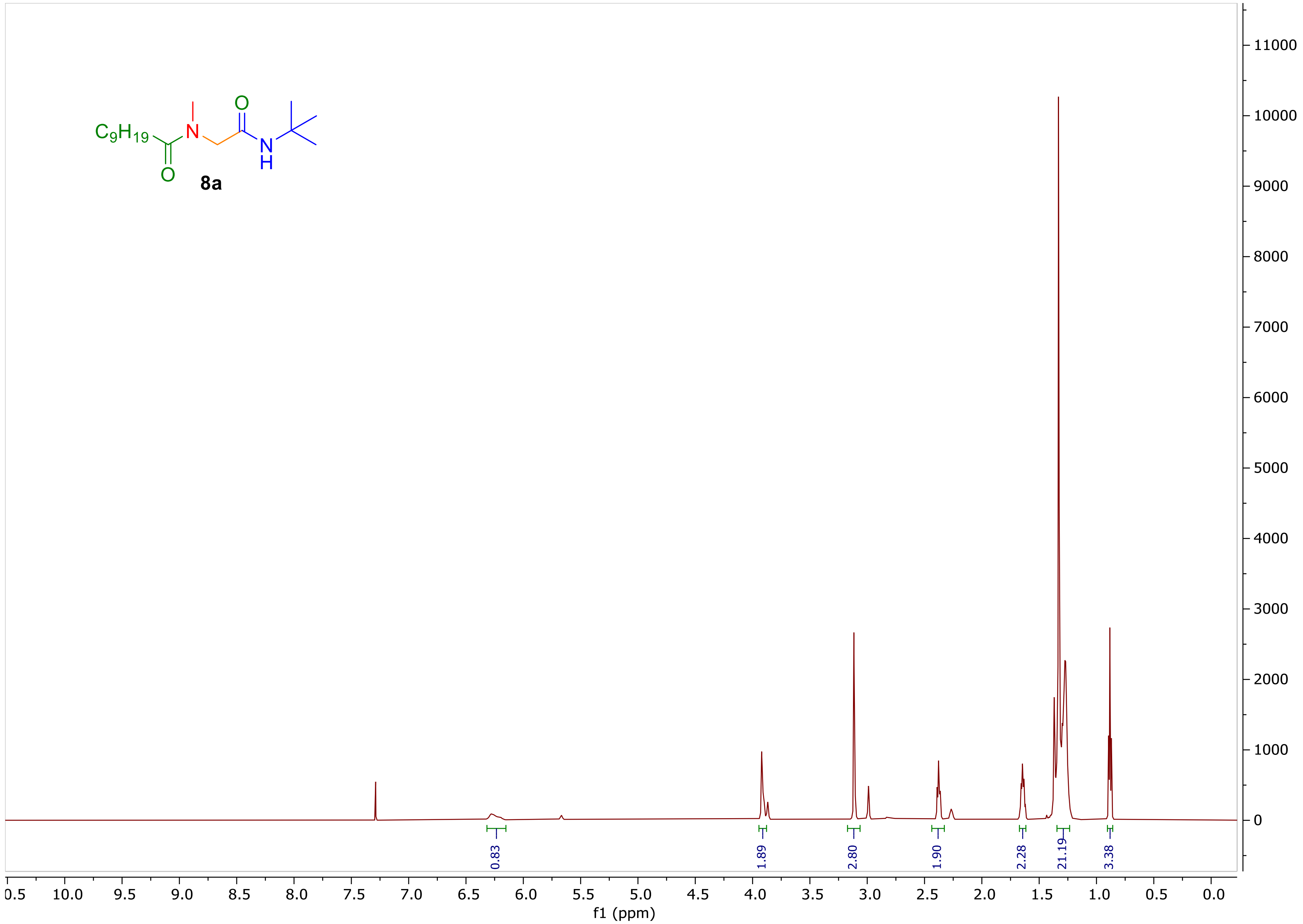

Figure S10. ^{1}H NMR (600 MHz, $CDCl_3$) Spectrum of compound **8a**.

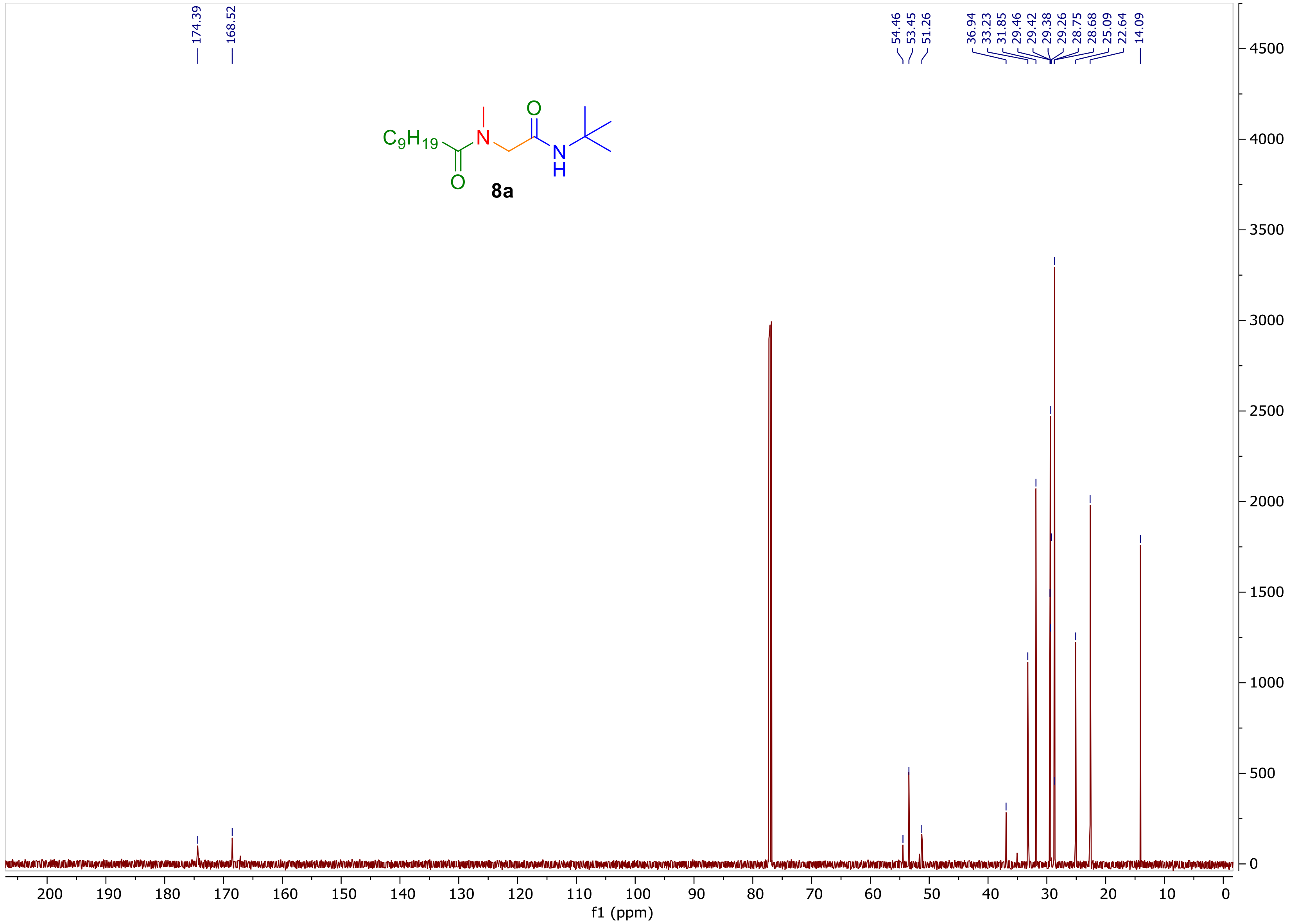

Figure S11. 13C NMR (151 MHz, CDCl3) Spectrum of compound **8a**.

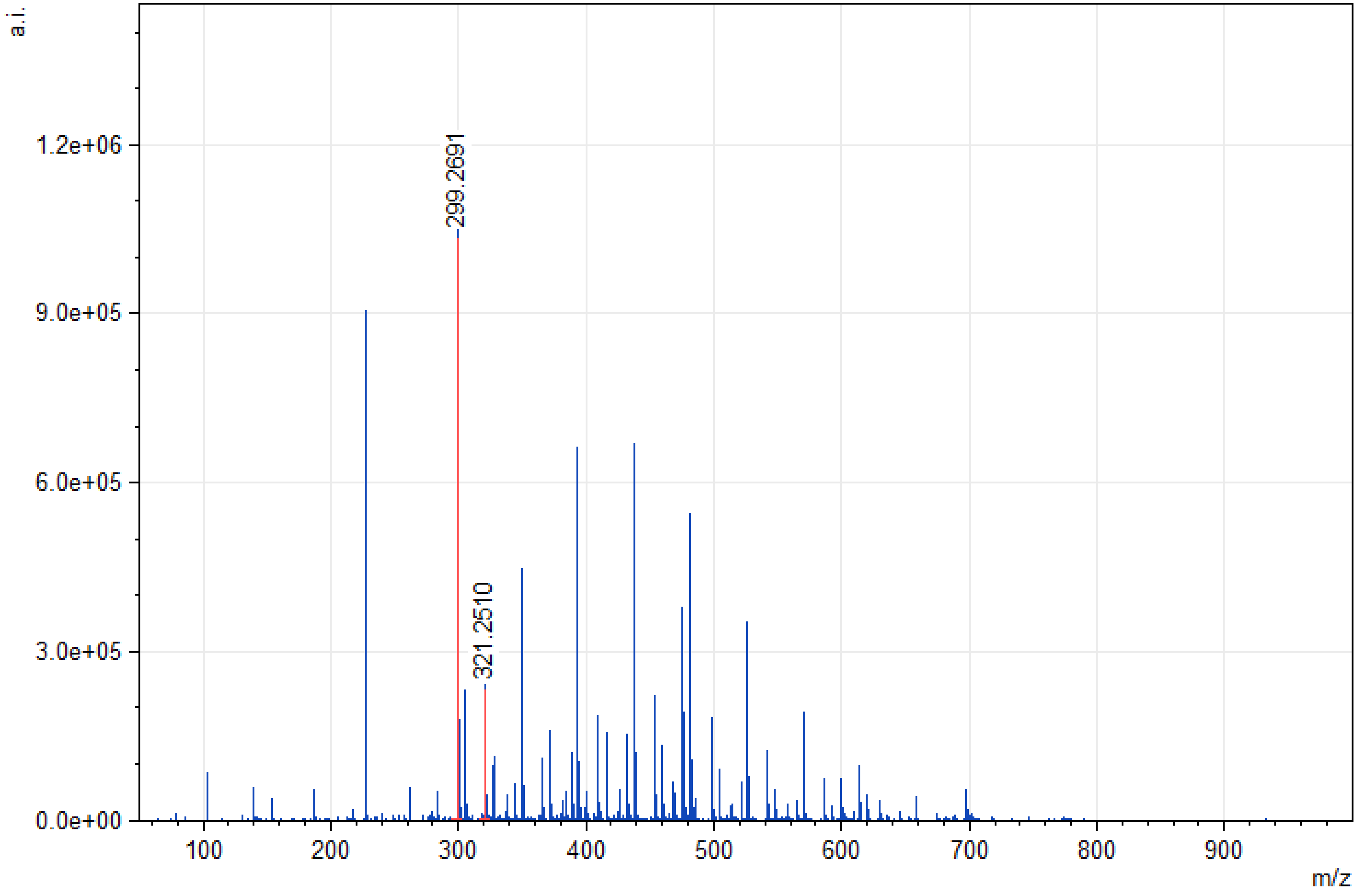

Figure S12. HRMS of compound **8a**.

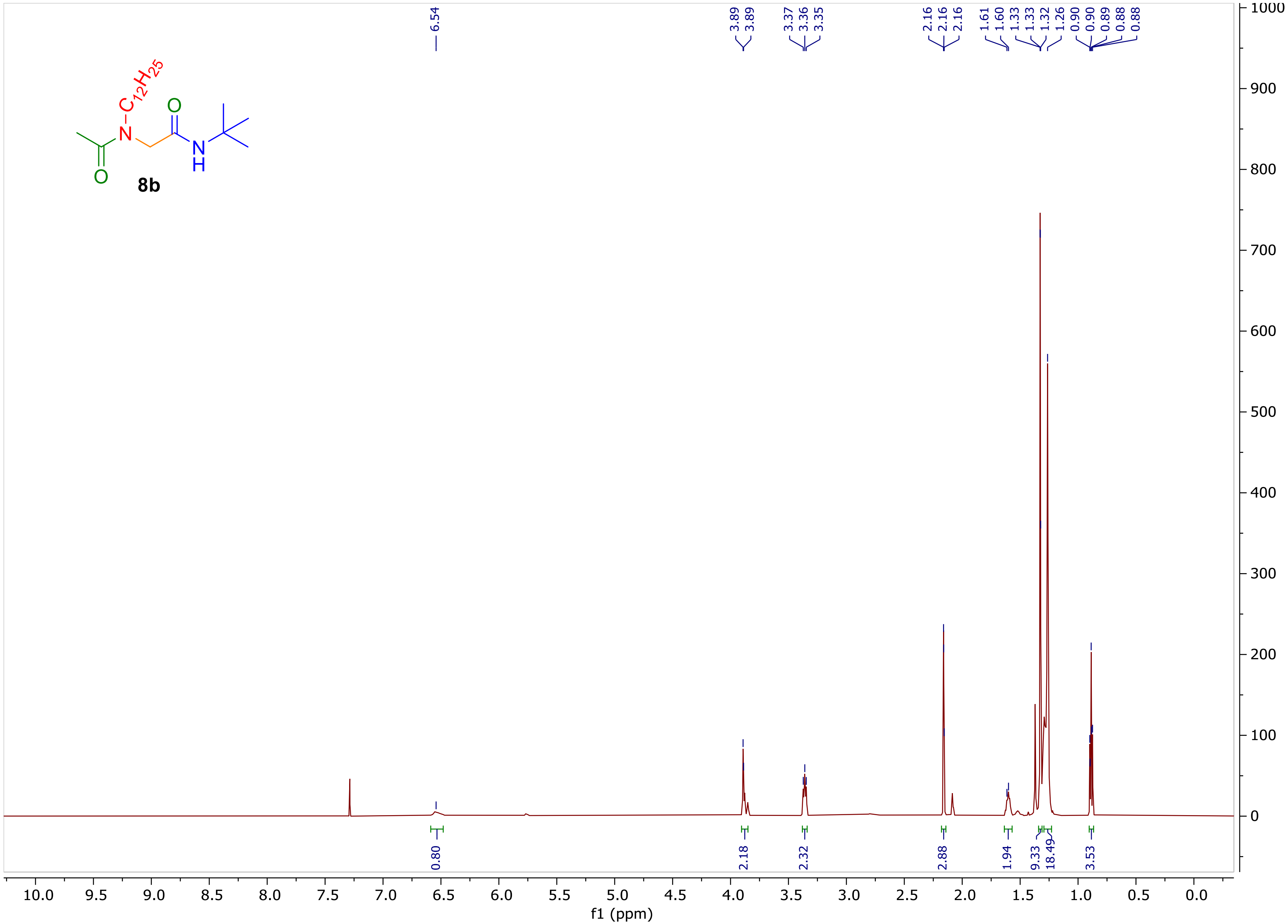

Figure S13. ^{1}H NMR (600 MHz, $CDCl_3$) Spectrum of compound **8b**.

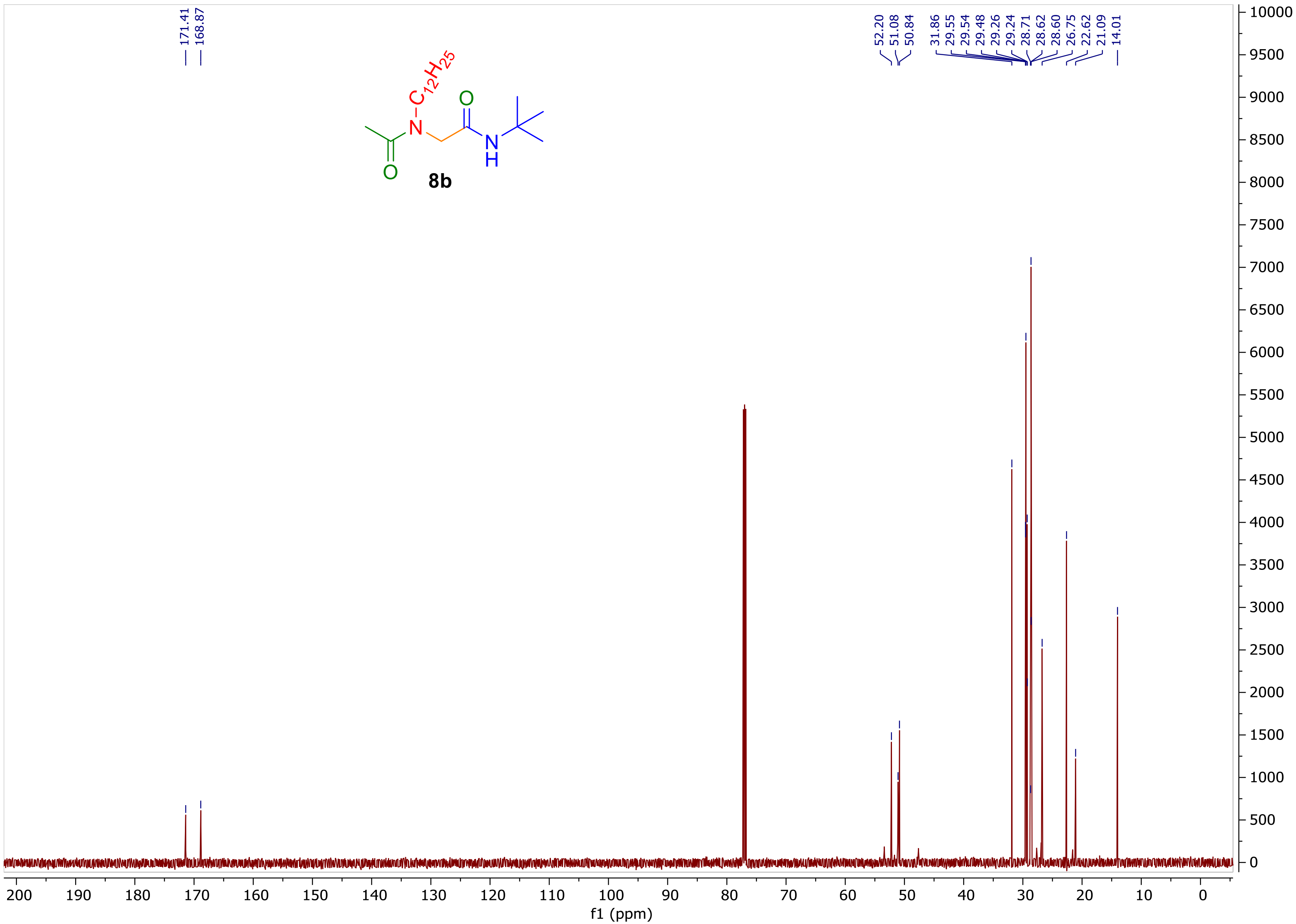

Figure S14. ^{13}C NMR (151 MHz, $CDCl_3$) Spectrum of compound **8b**.

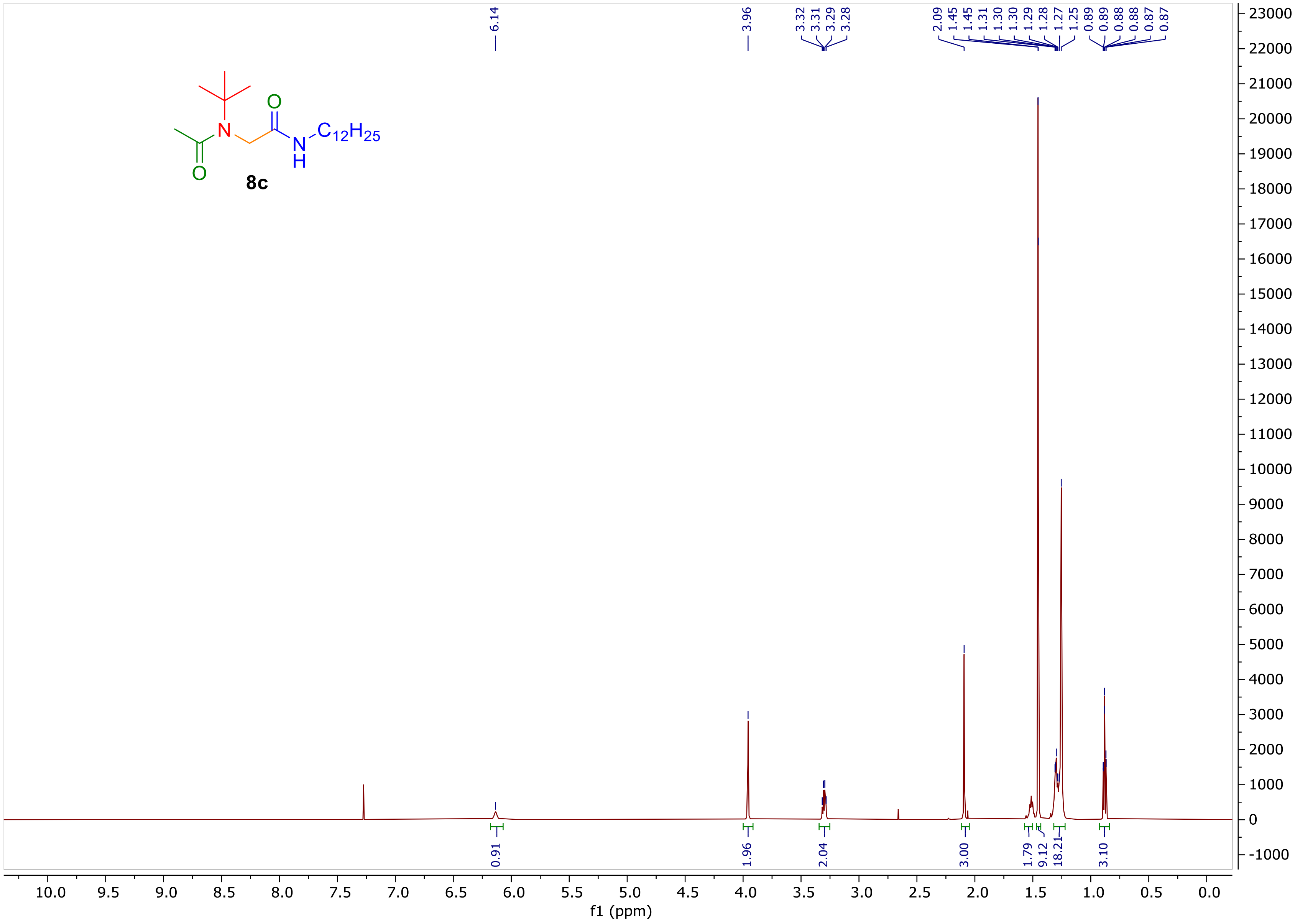

Figure S15. ^{1}H NMR (600 MHz, $CDCl_3$) Spectrum of compound **8c**.

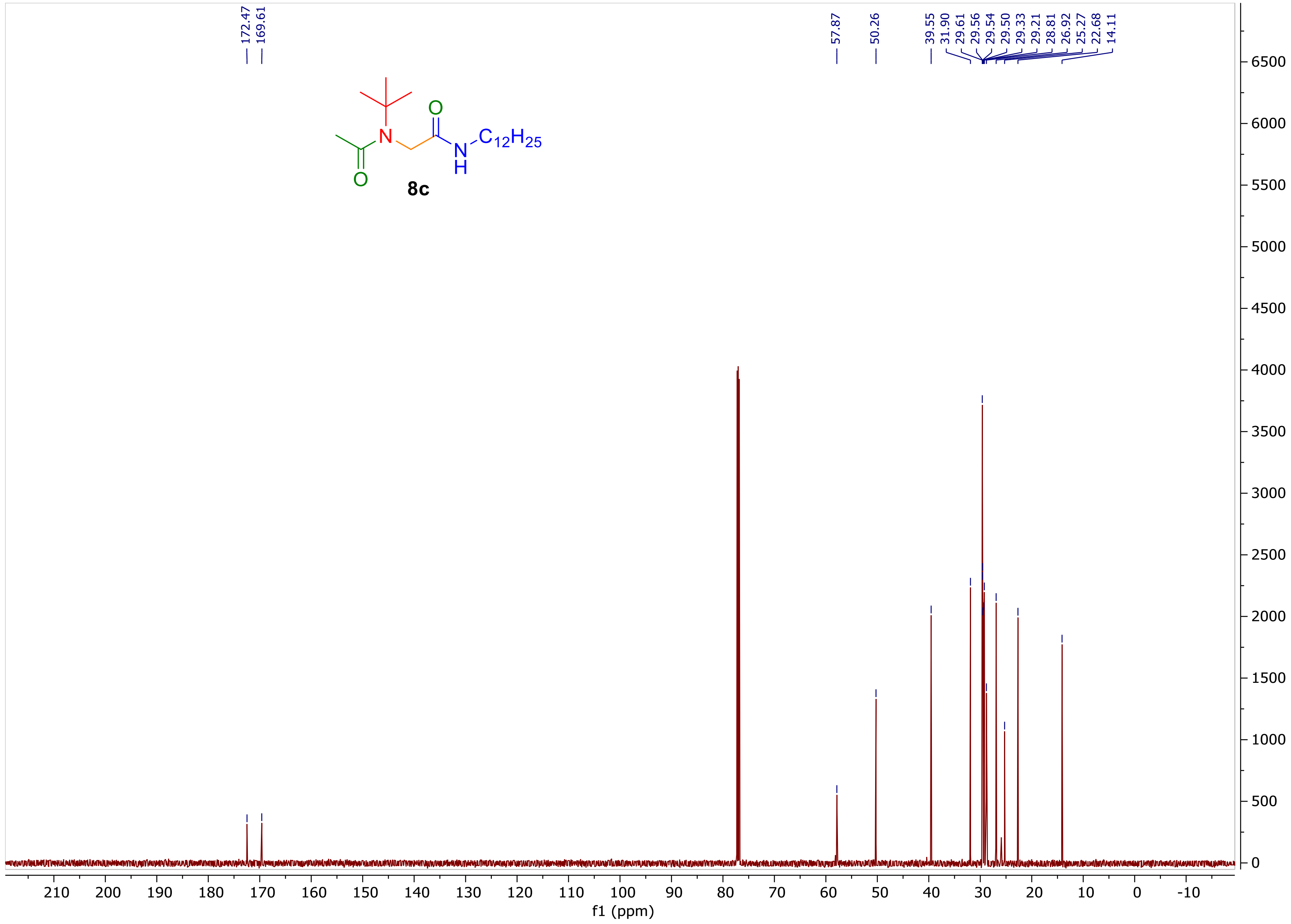

Figure S16. ${}^{13}C$ NMR (151 MHz, $CDCl_3$) Spectrum of compound **8c**.

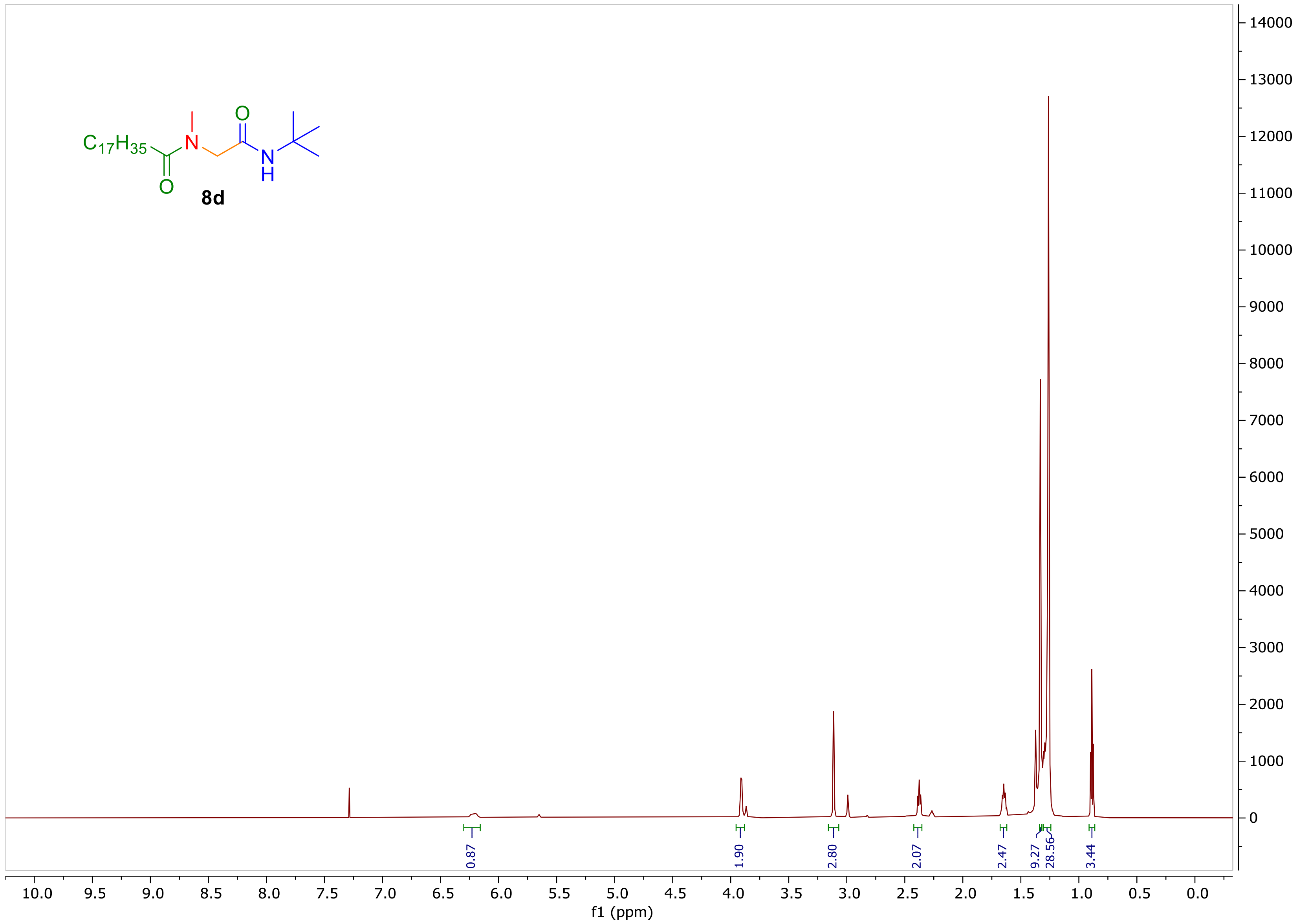

Figure S17. ^{1}H NMR (600 MHz, $CDCl_3$) Spectrum of compound **8d**.

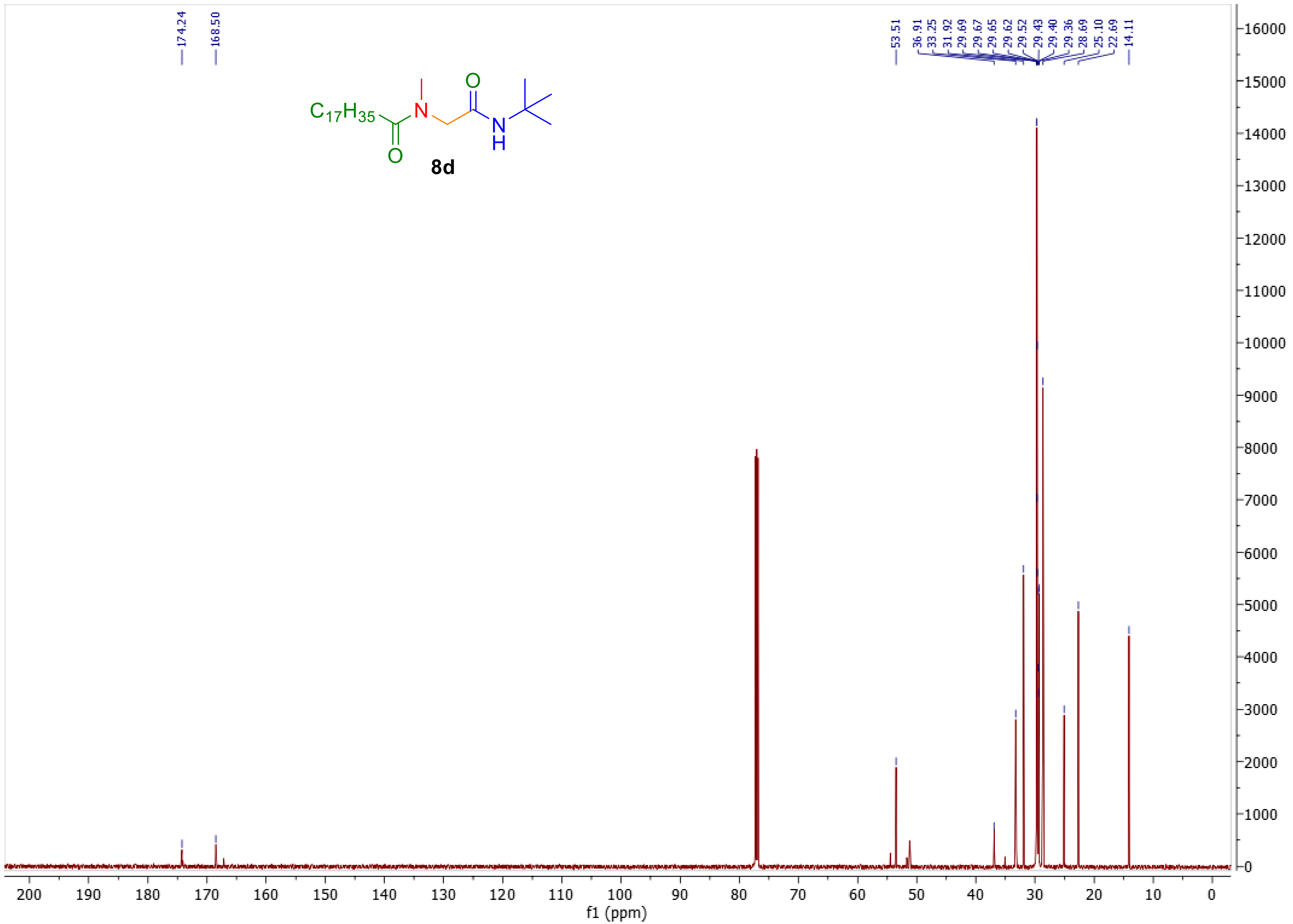

Figure S18. ^{13}C NMR (151 MHz, $CDCl_3$) Spectrum of compound **8d**.

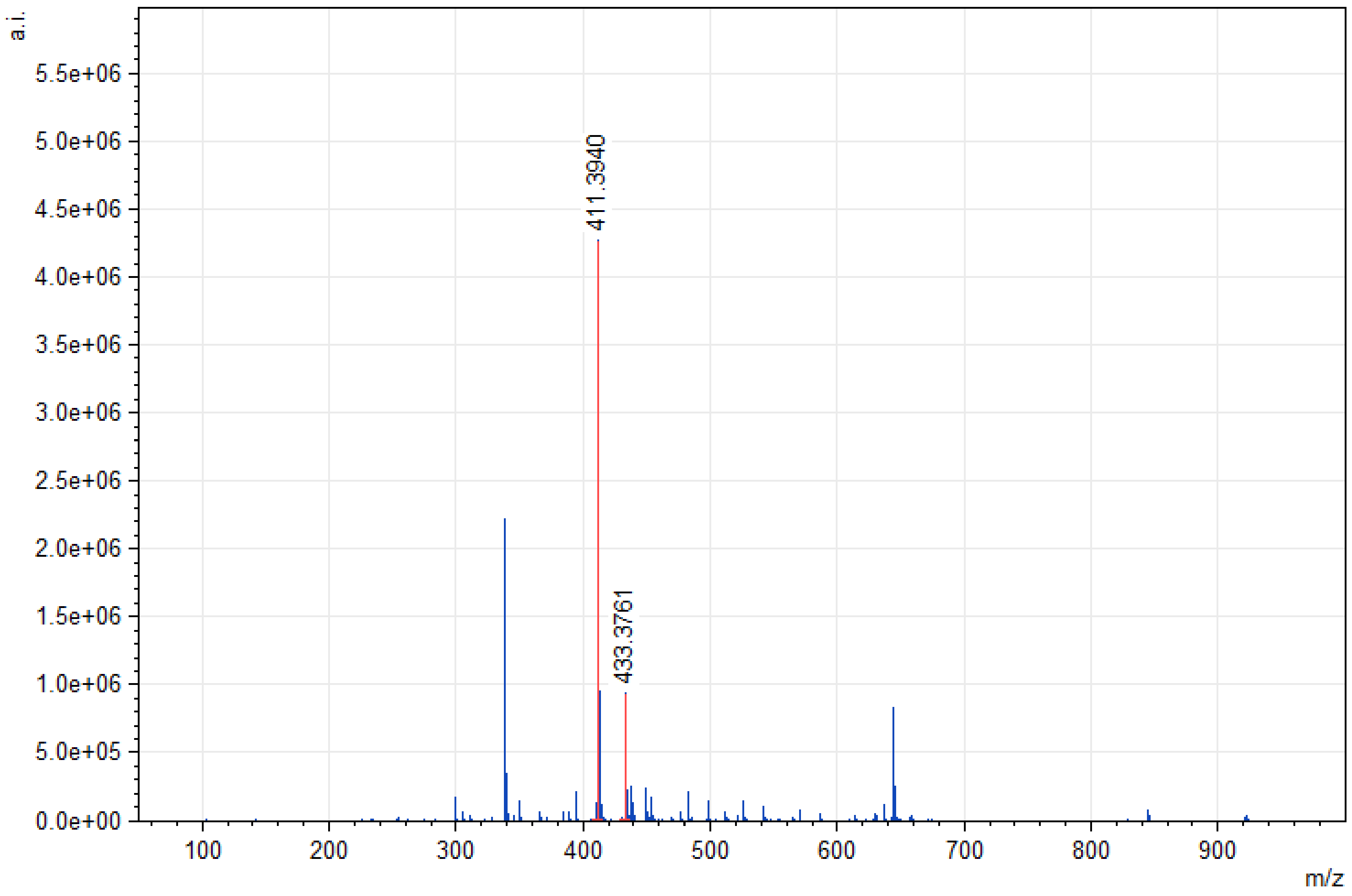

Figure S19. HRMS of compound **8d**.

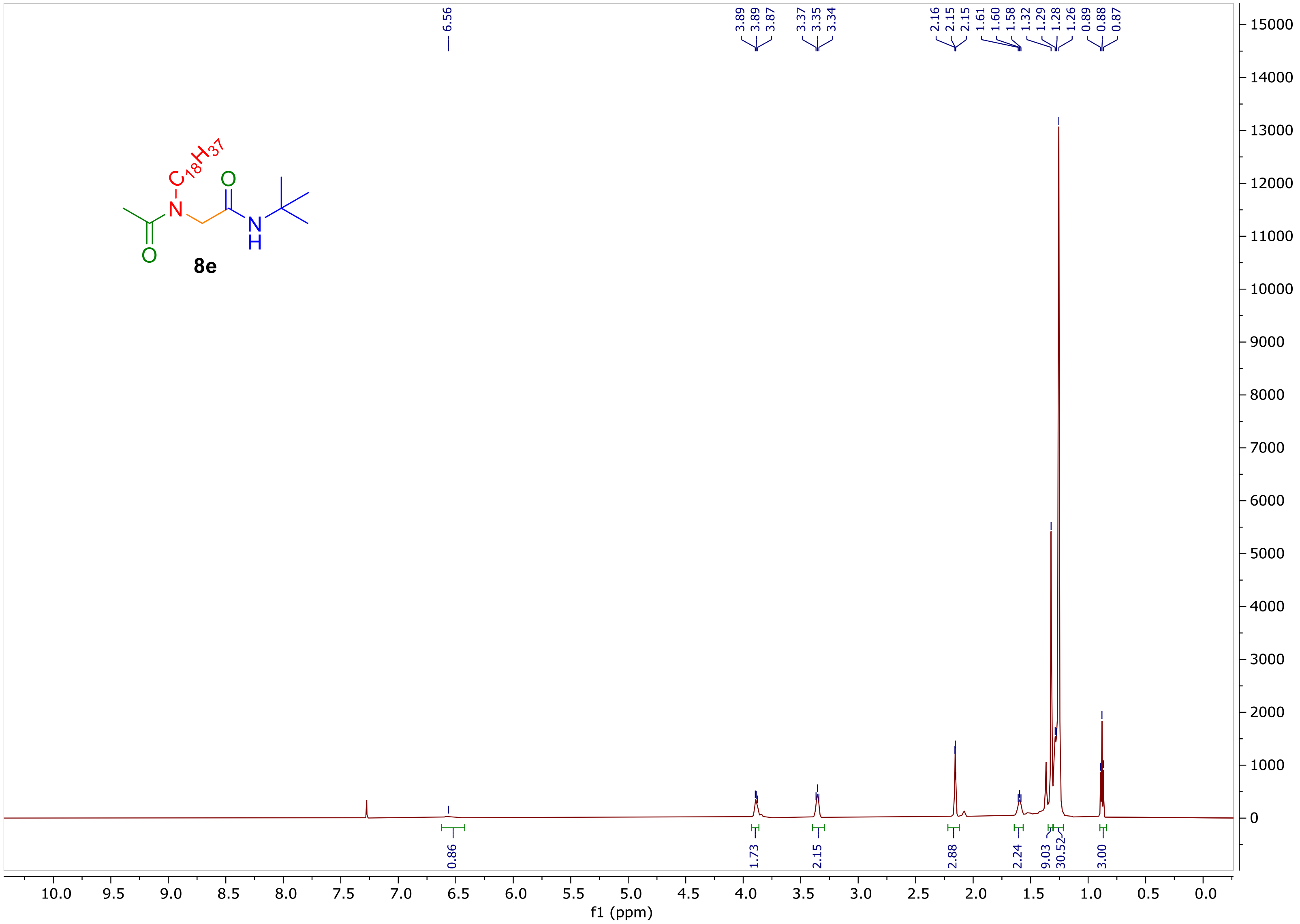

Figure S20. ^{1}H NMR (600 MHz, $CDCl_3$) Spectrum of compound **8e**.

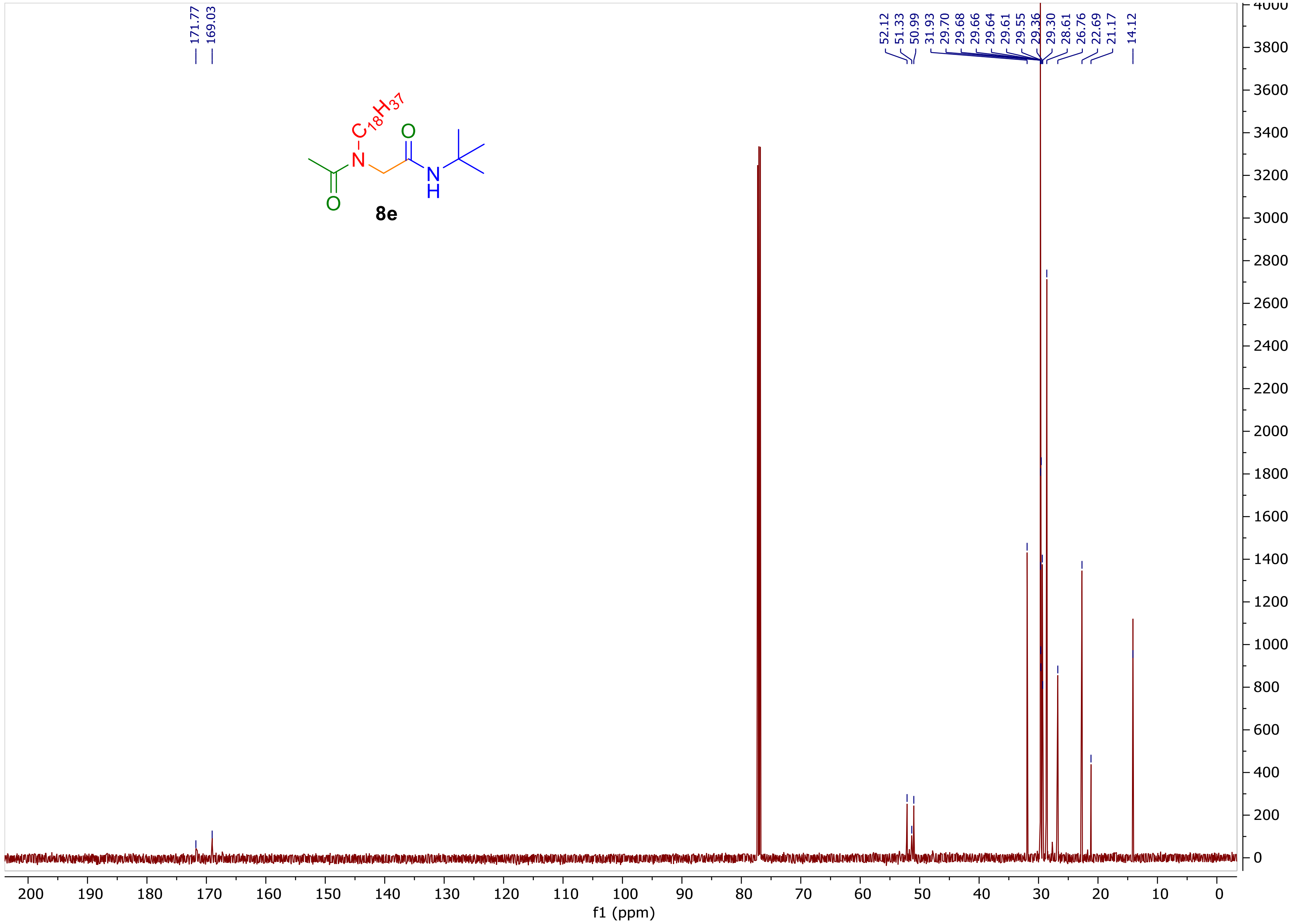

Figure S21. ^{13}C NMR (151 MHz, $CDCl_3$) Spectrum of compound **8e**.

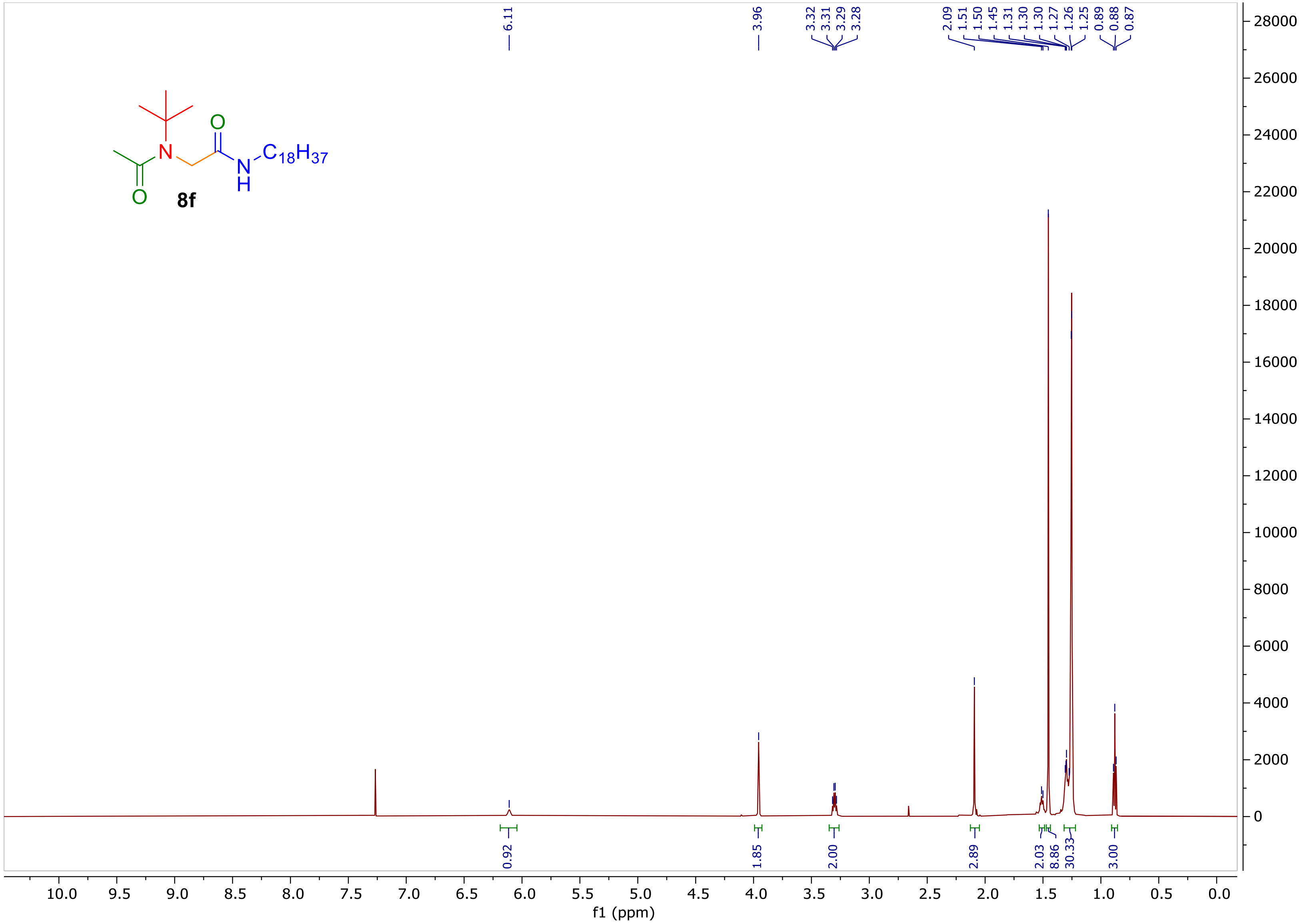

Figure S22. ^{1}H NMR (600 MHz, $CDCl_3$) Spectrum of compound **8f**.

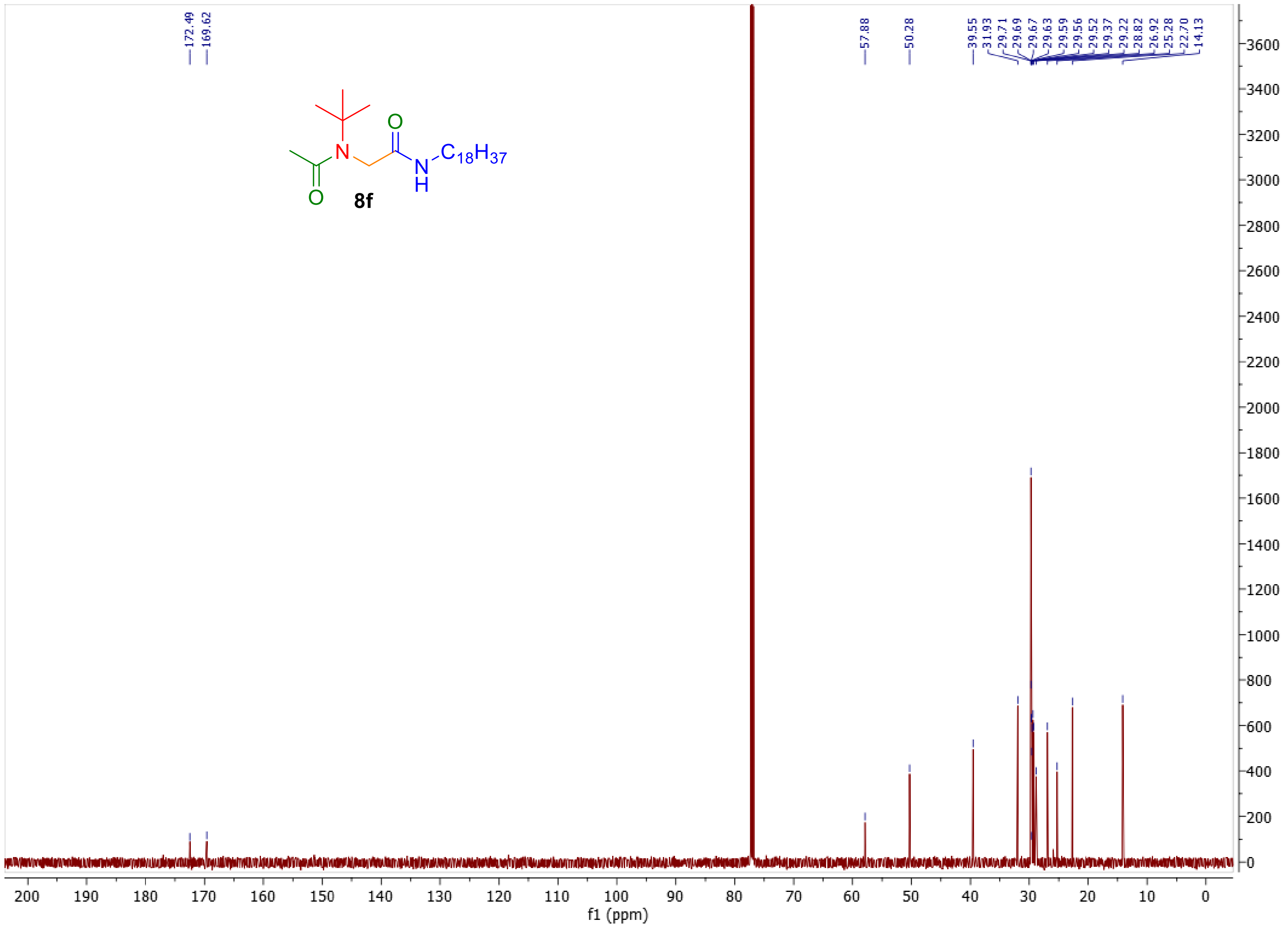

Figure S23. ^{13}C NMR (151 MHz, $CDCl_3$) Spectrum of compound **8f**.

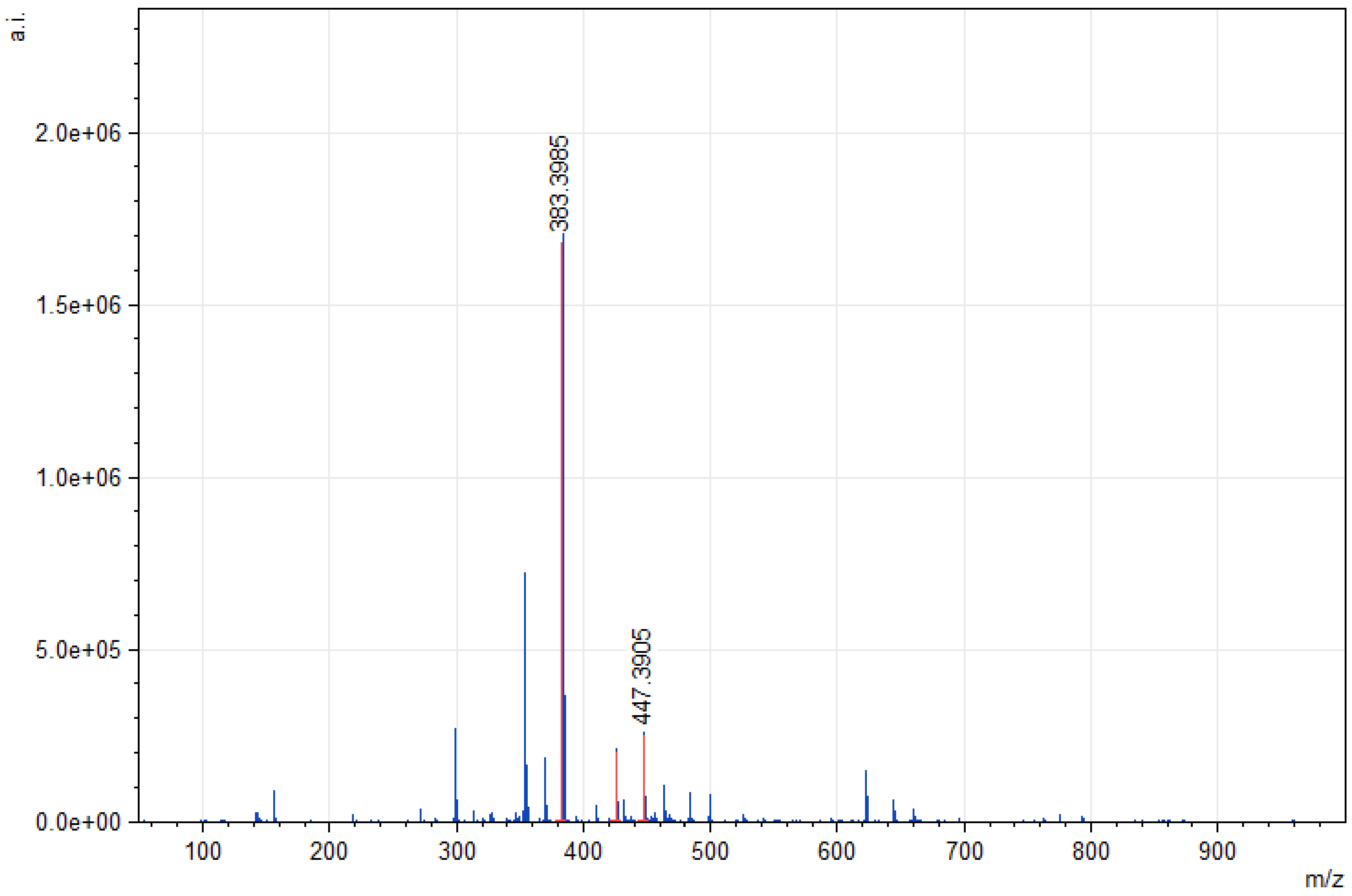

Figure S24. HRMS of compound **8f**.

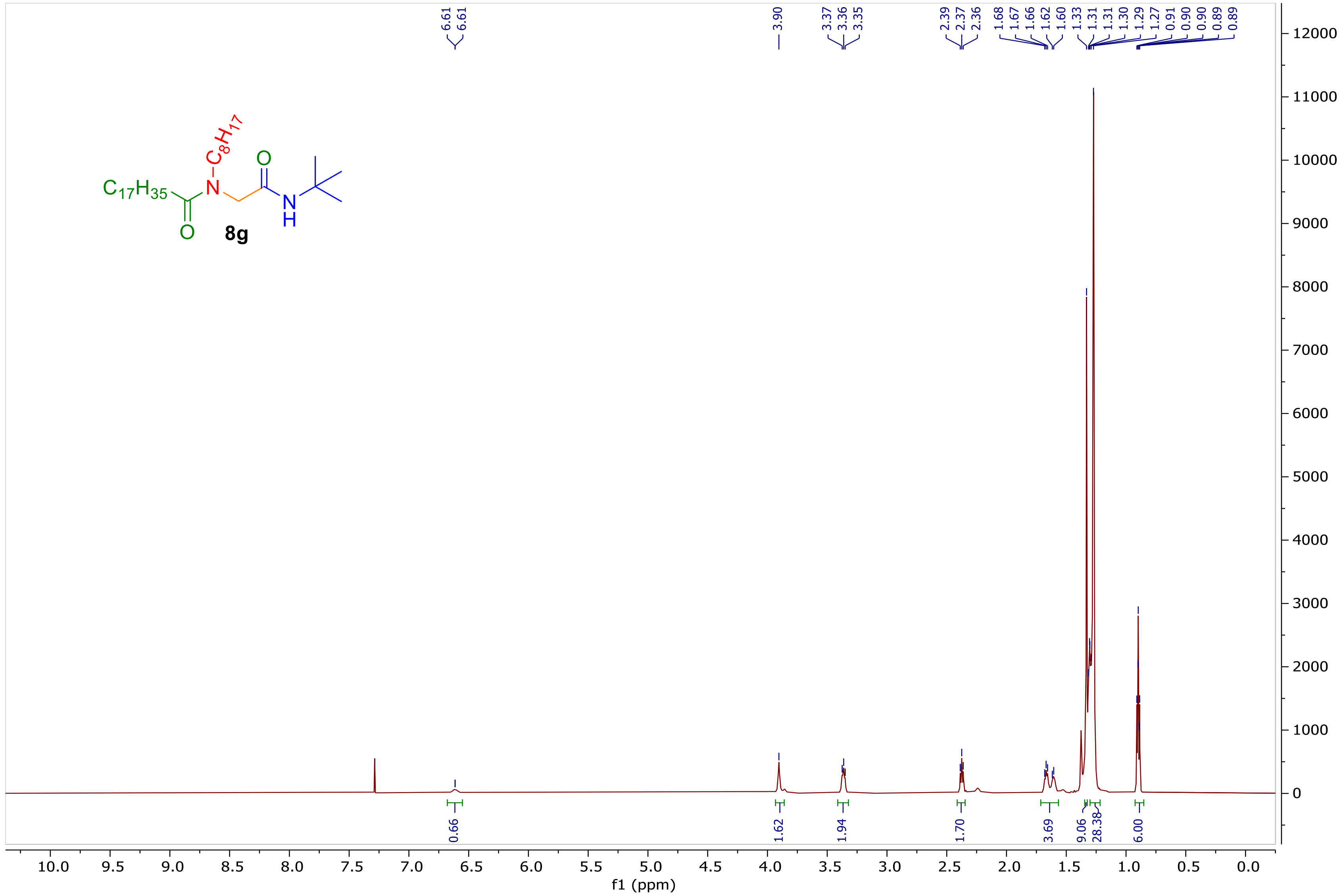

Figure S25. ^{1}H NMR (600 MHz, $CDCl_3$) Spectrum of compound **8g**.

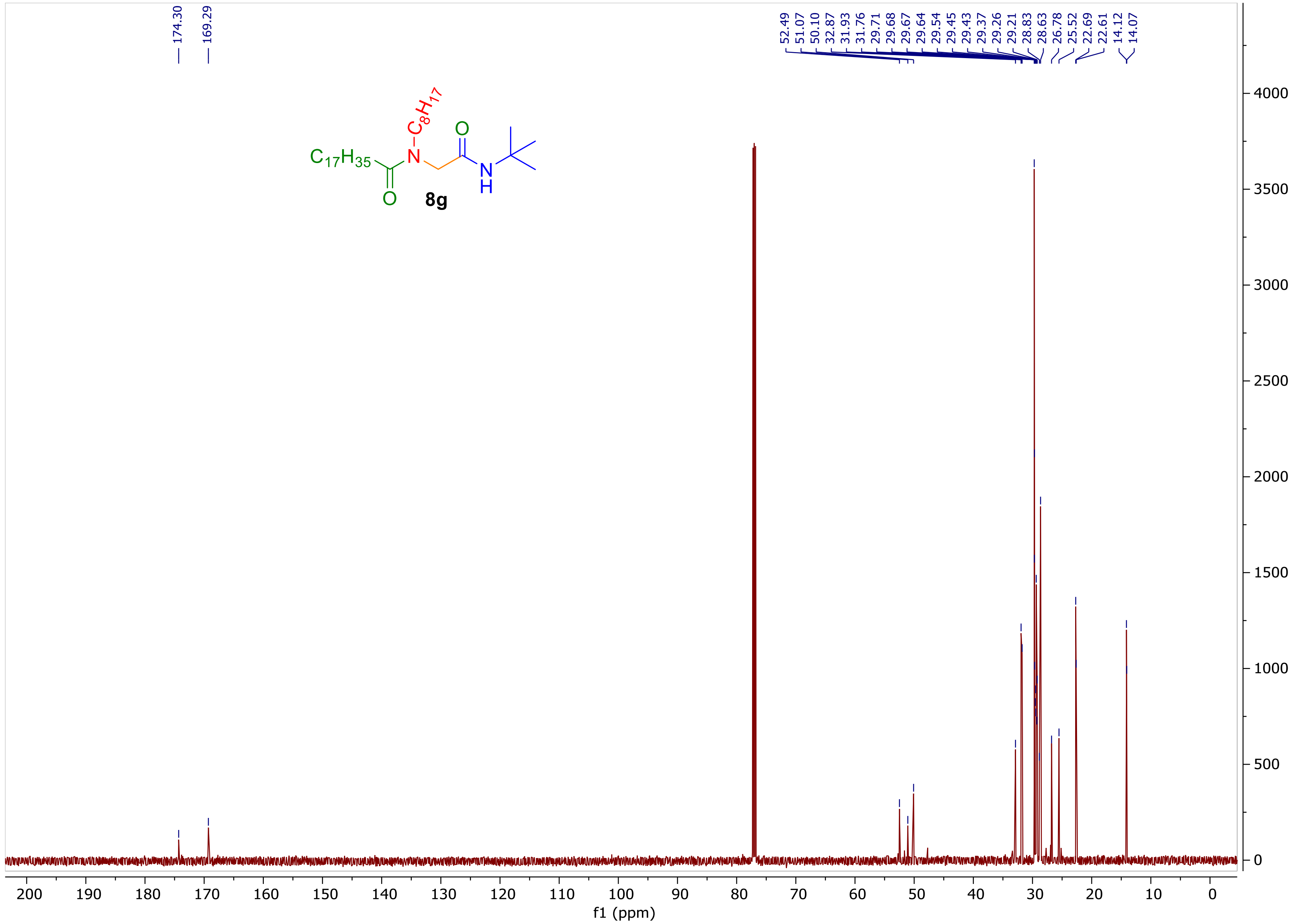

Figure S26. ^{13}C NMR (151 MHz, $CDCl_3$) Spectrum of compound **8g**.

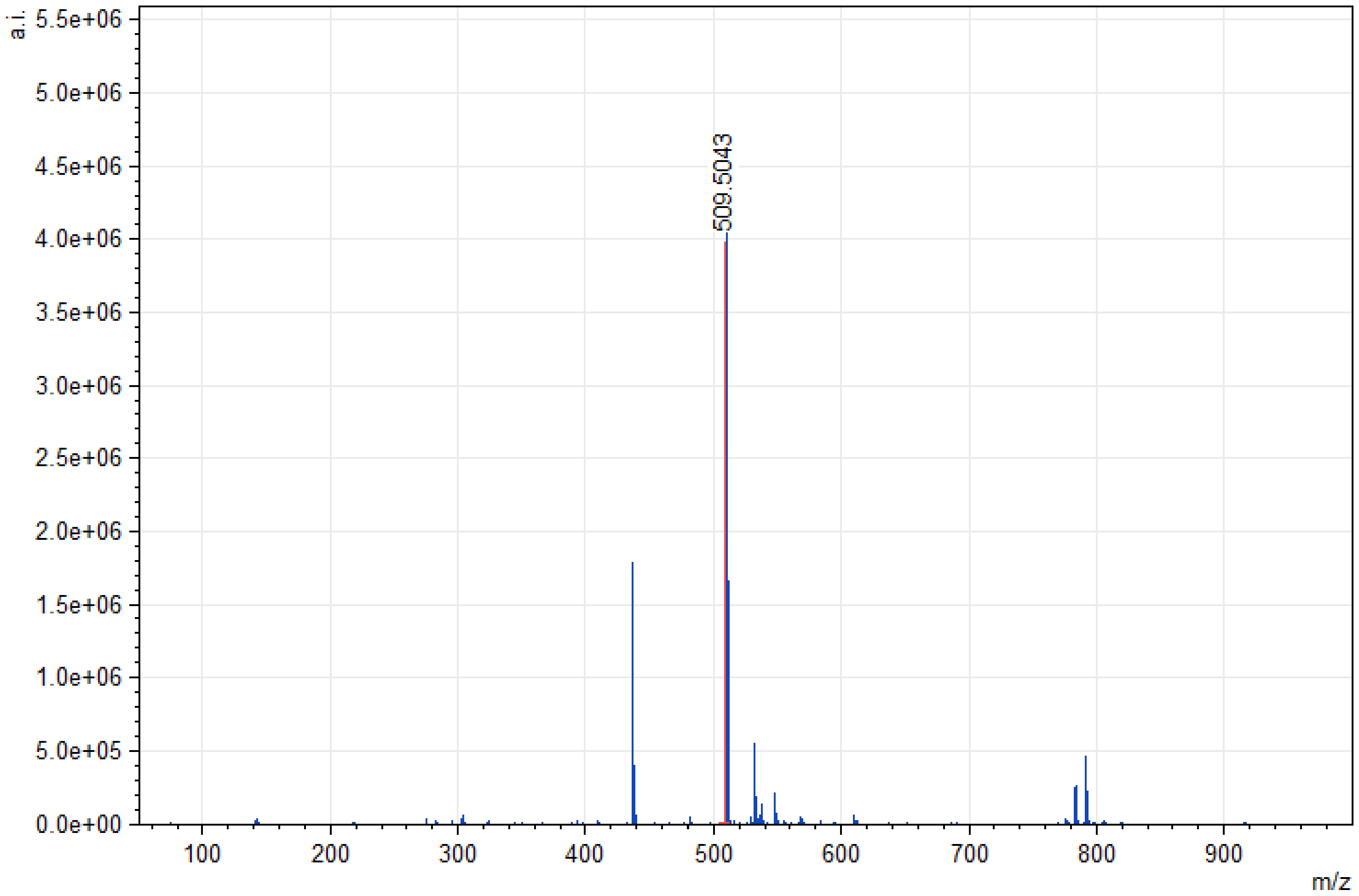

Figure S27. HRMS of compound **8g**.

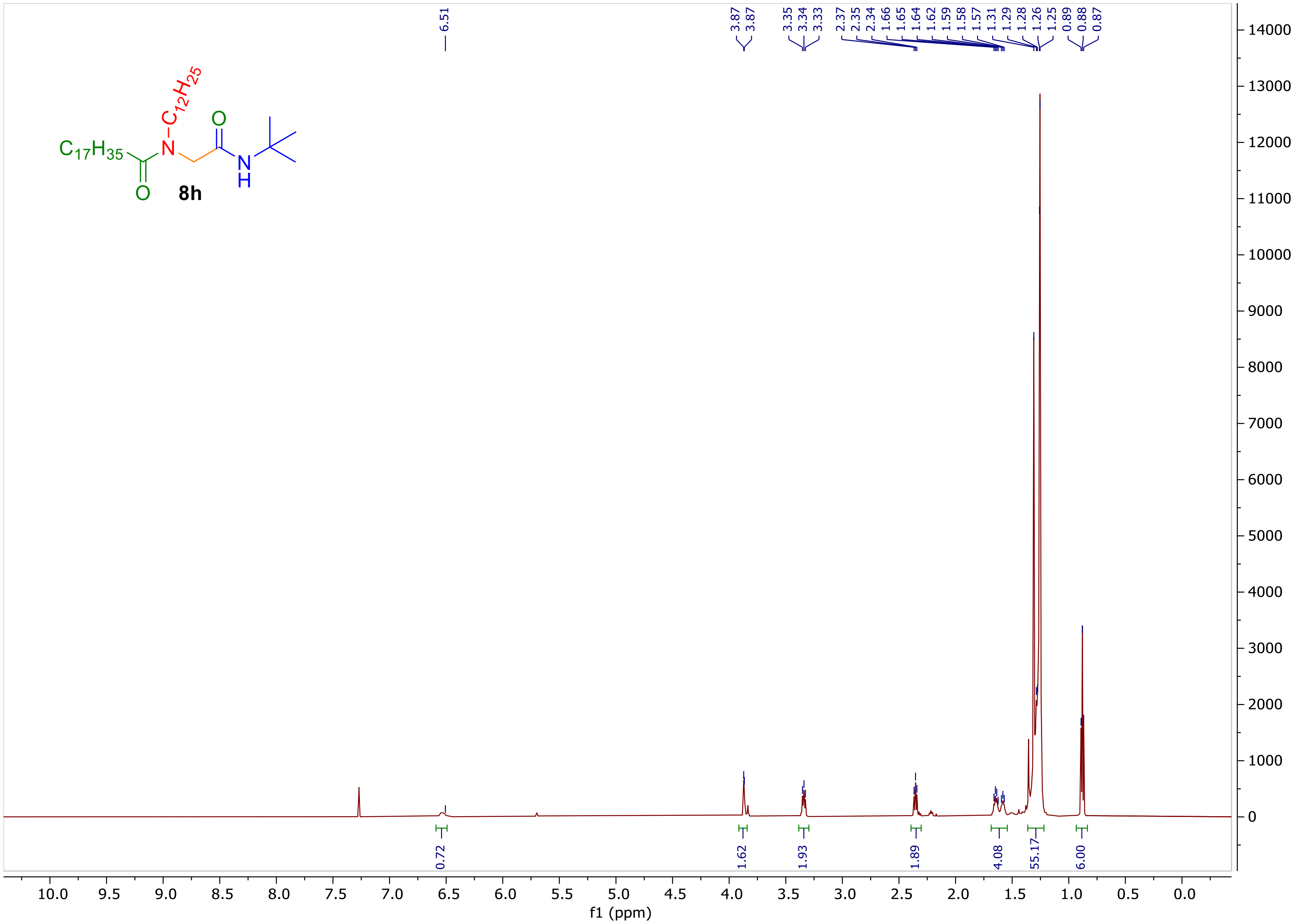

Figure S28. ¹H NMR (600 MHz, $CDCl_3$) Spectrum of compound **8h.**

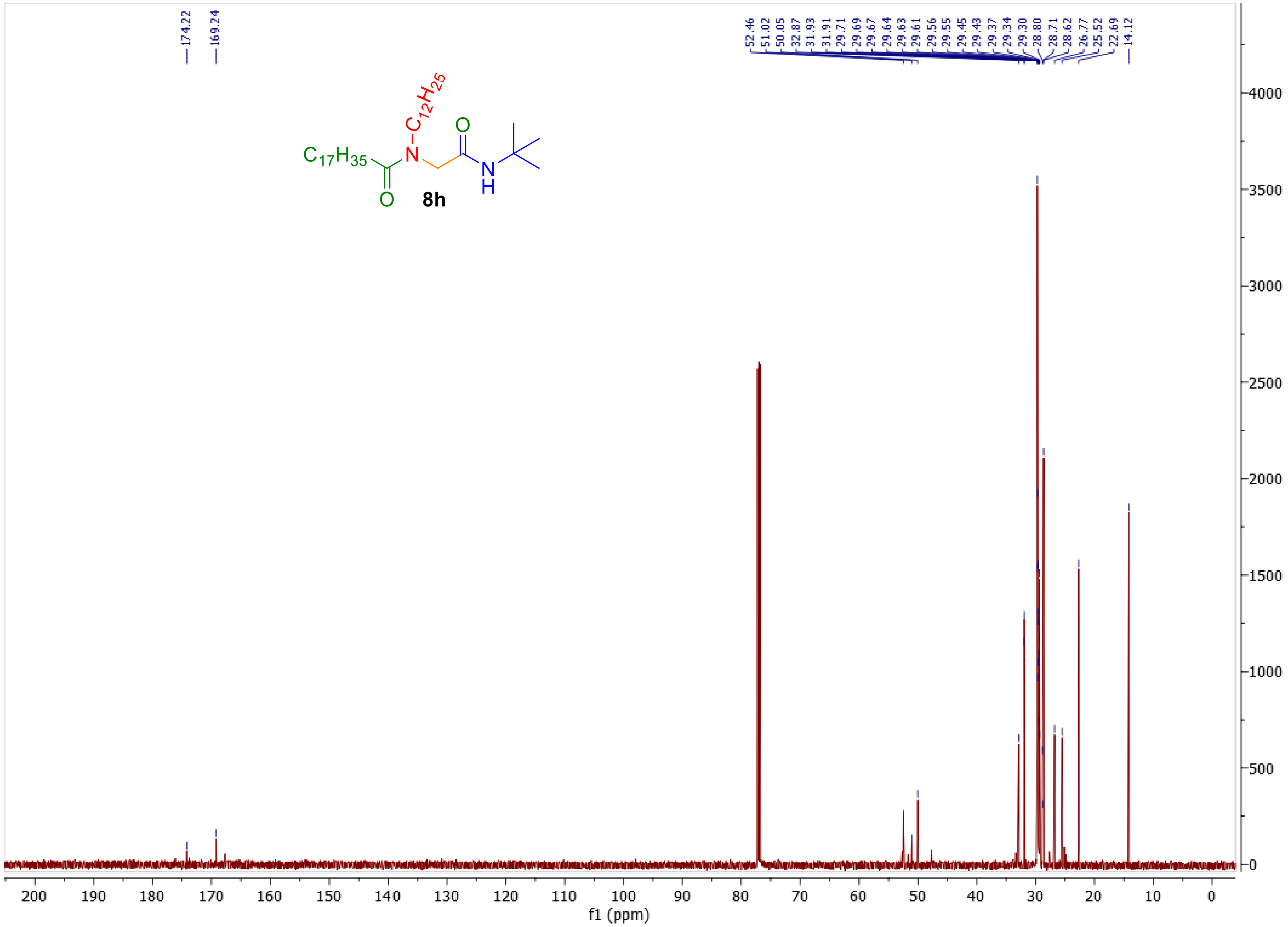

Figure S29. ^{13}C NMR (151 MHz, $CDCl_3$) Spectrum of compound **8h**.

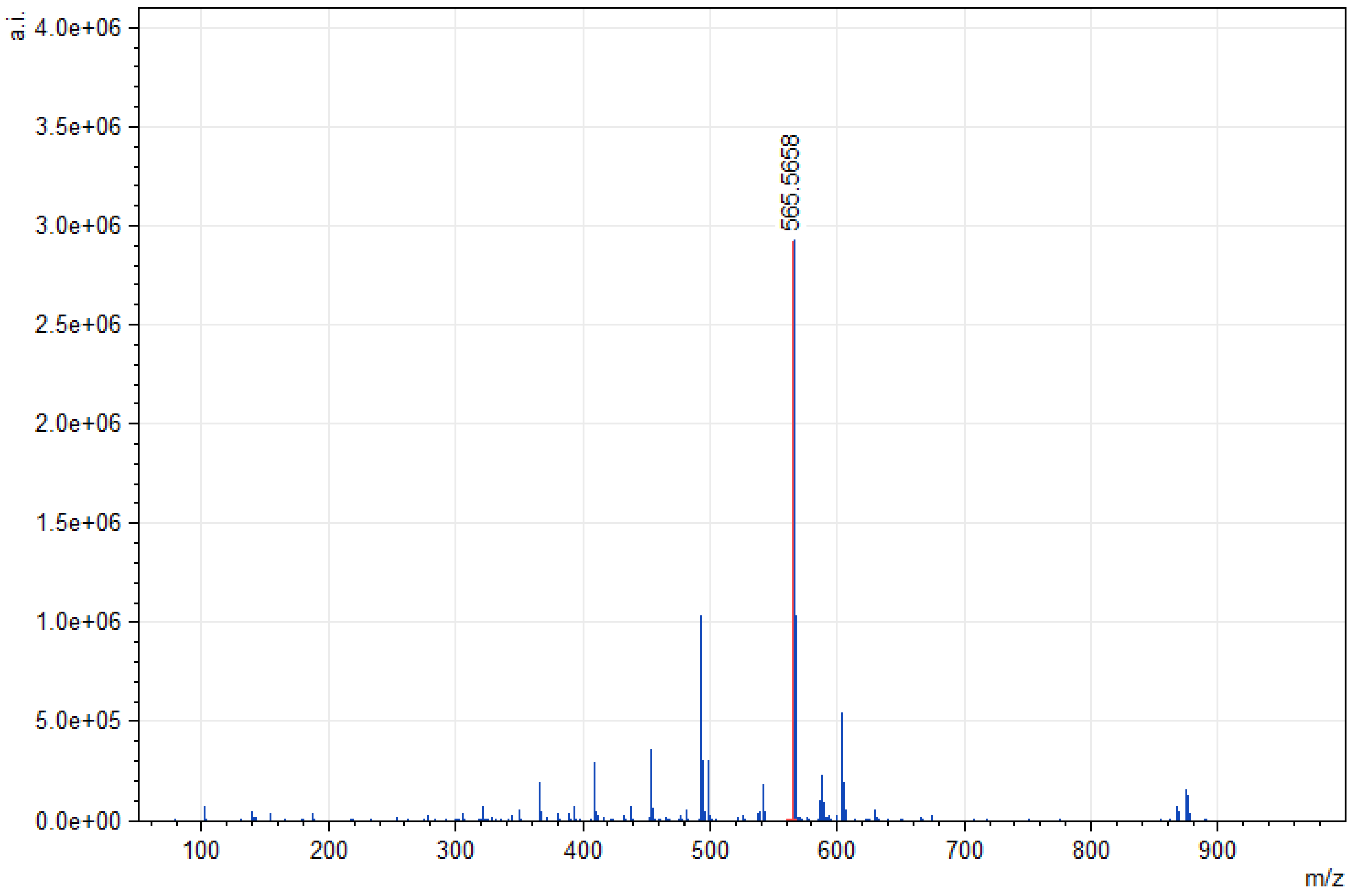

Figure S30. HRMS of compound **8h**.

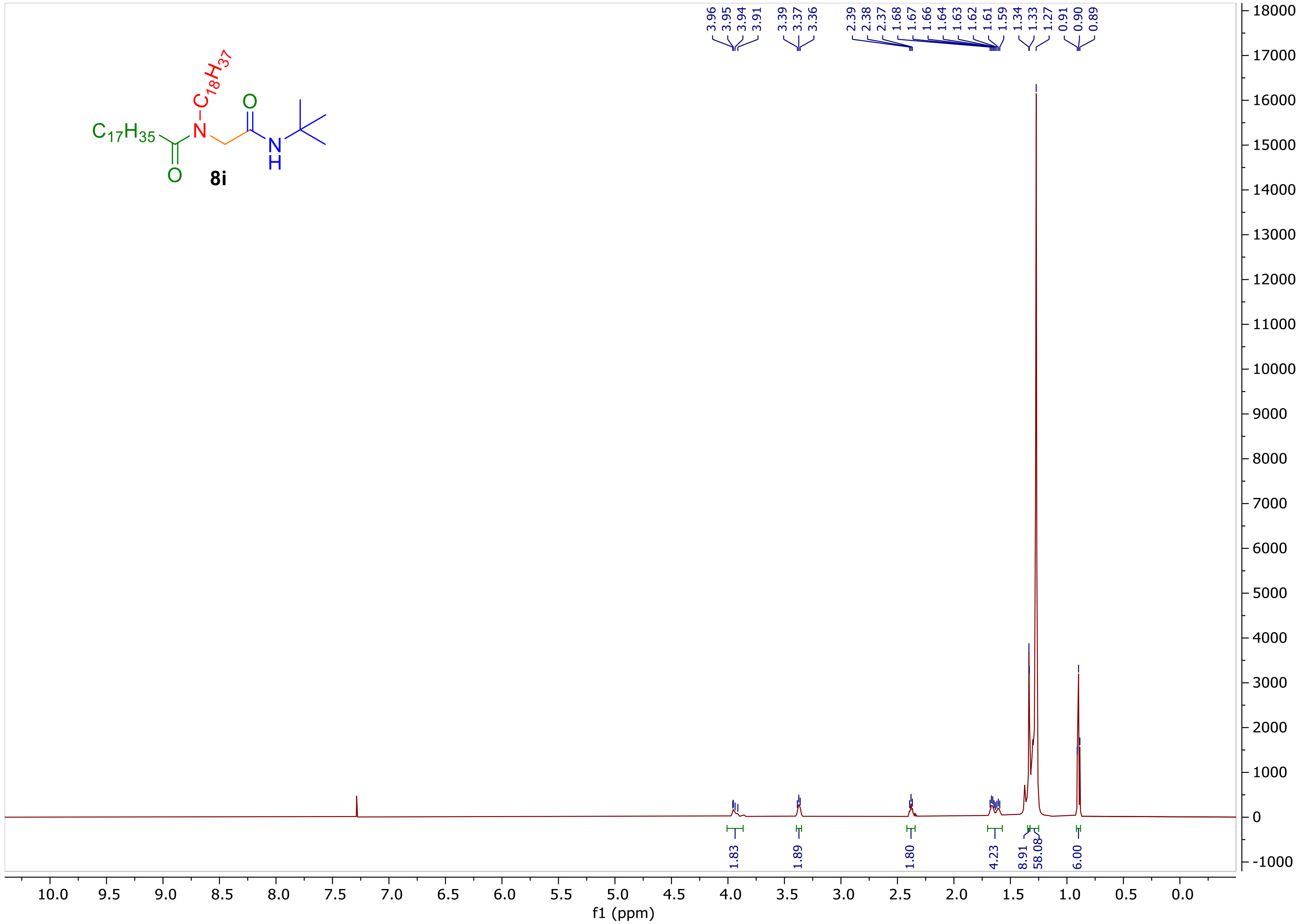

Figure S31. ^{1}H NMR (600 MHz, $CDCl_3$) Spectrum of compound **8i.**

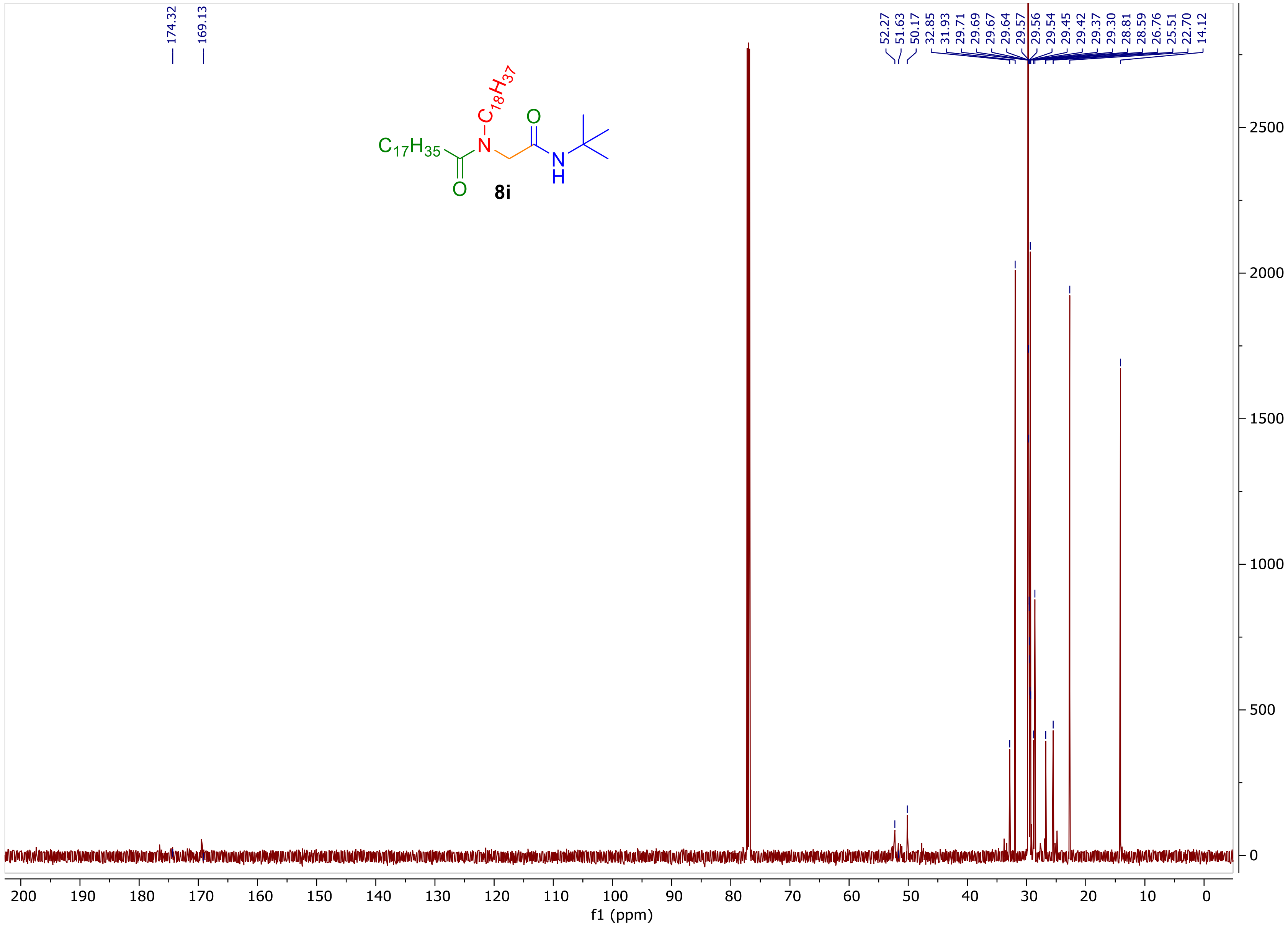

Figure S32. ^{13}C NMR (151 MHz, $CDCl_3$) Spectrum of compound **8i.**

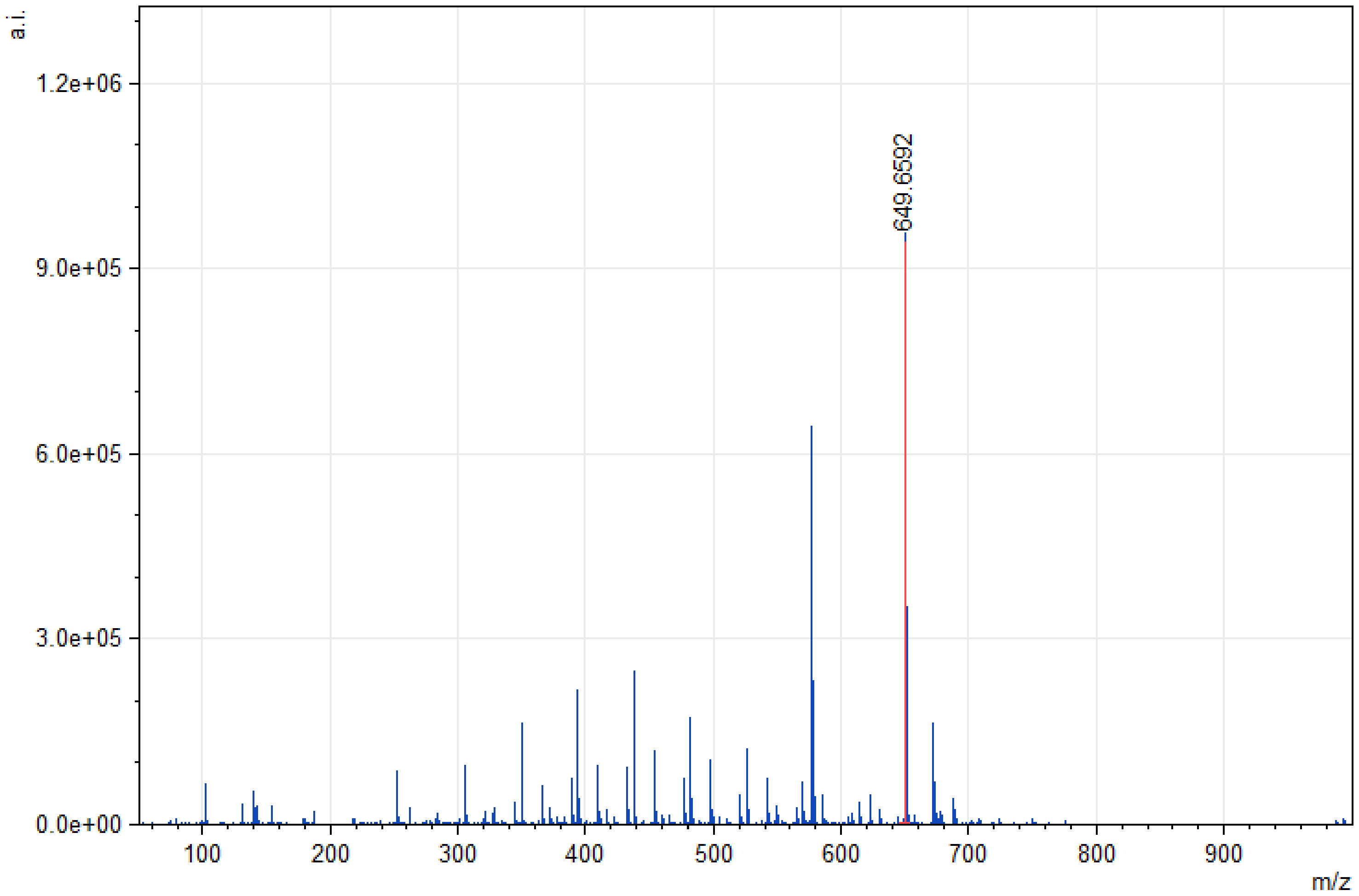

Figure S33. HRMS of compound **8i**.

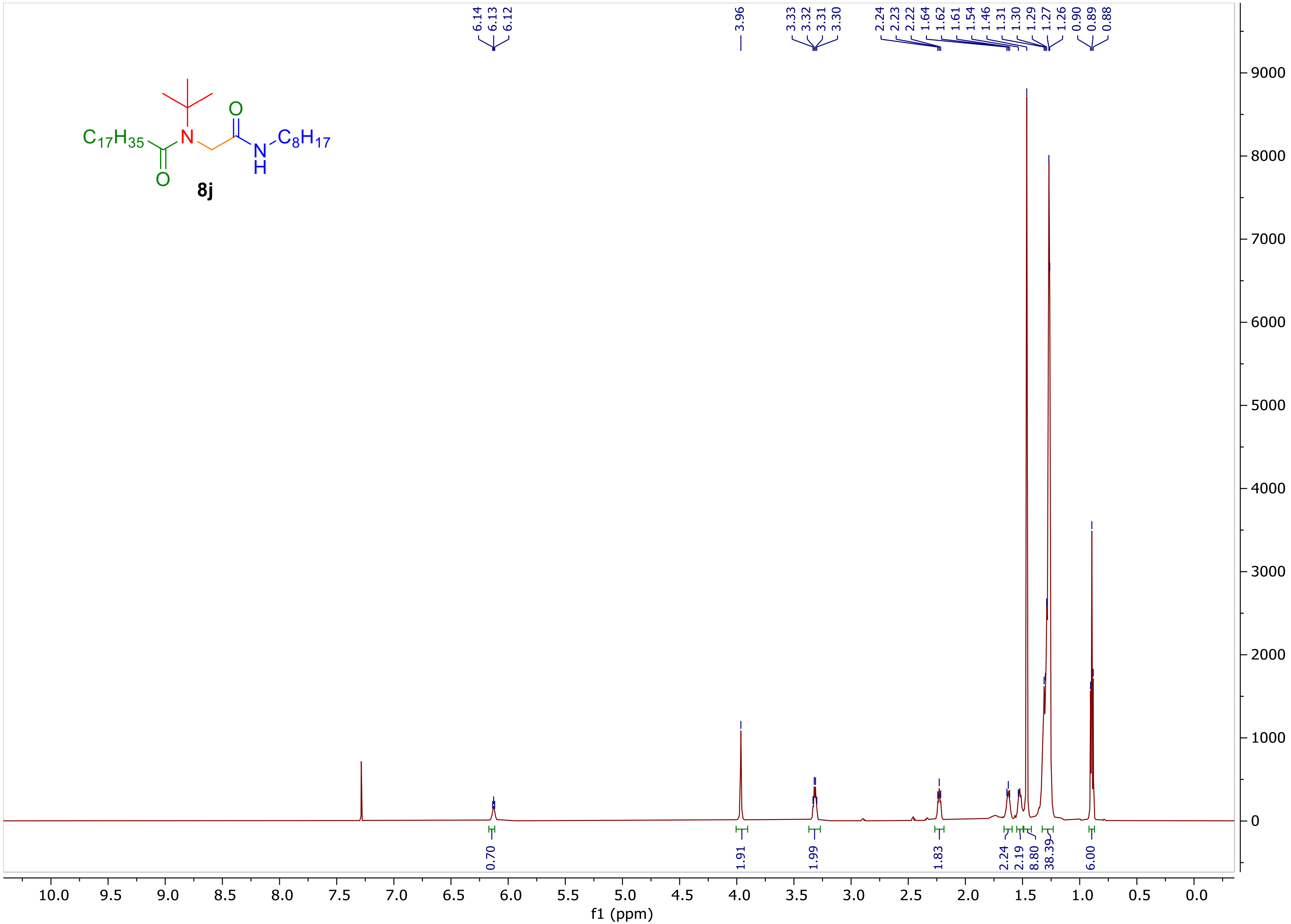

Figure S34. ^{1}H NMR (600 MHz, $CDCl_3$) Spectrum of compound **8j**.

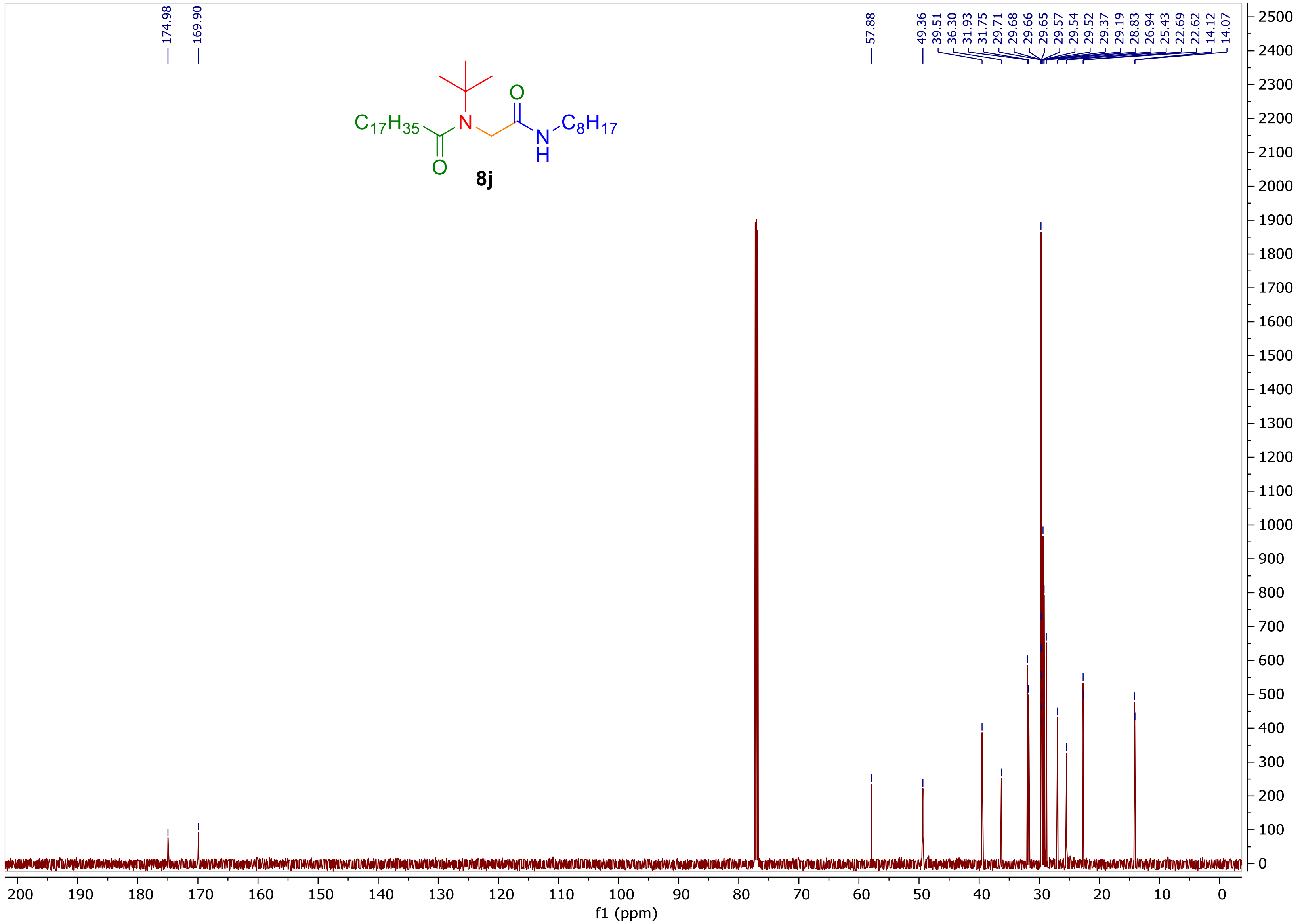

Figure S35. ^{13}C NMR (151 MHz, $CDCl_3$) Spectrum of compound **8j**.

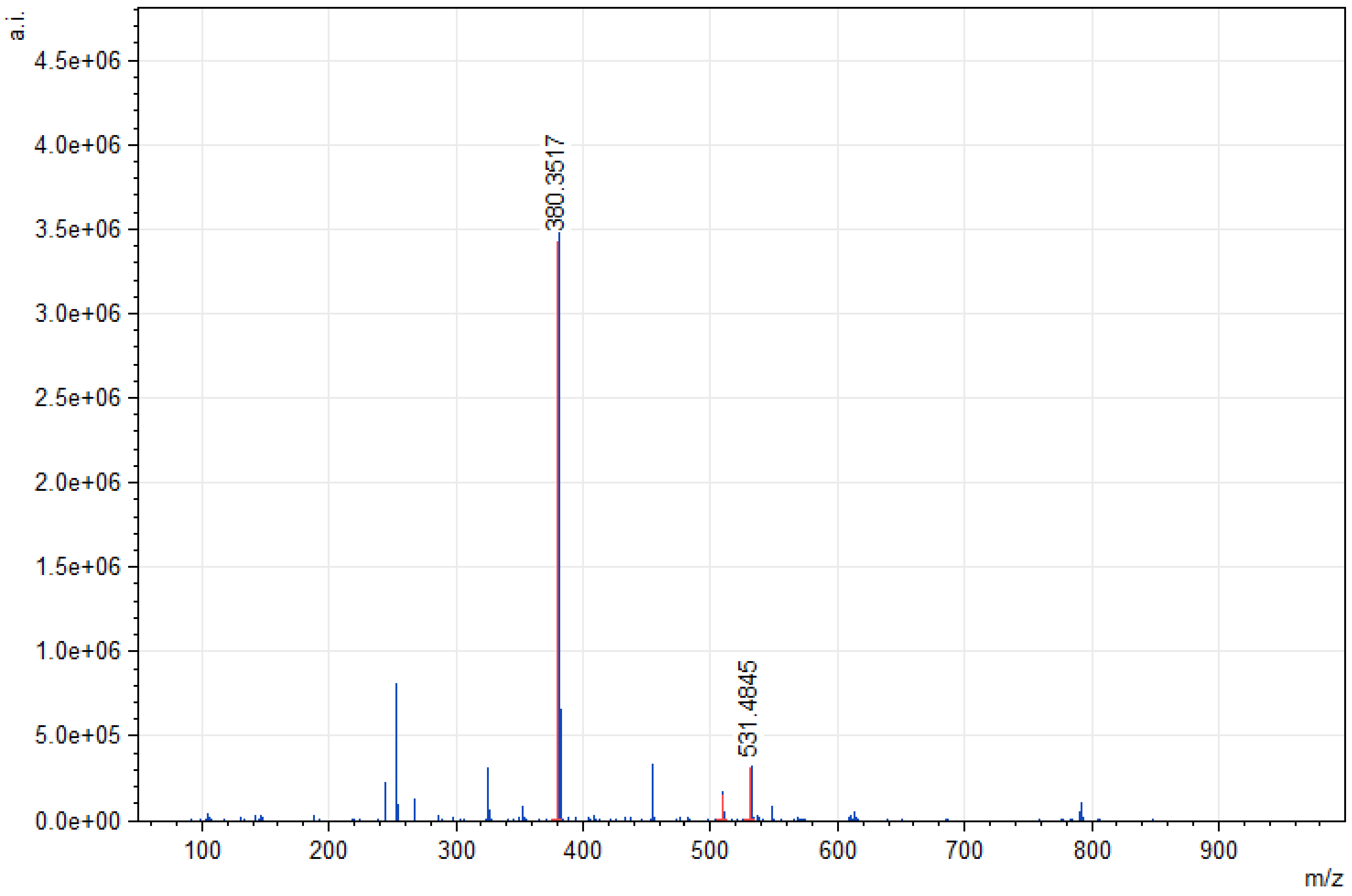

Figure S36. HRMS of compound **8j**.

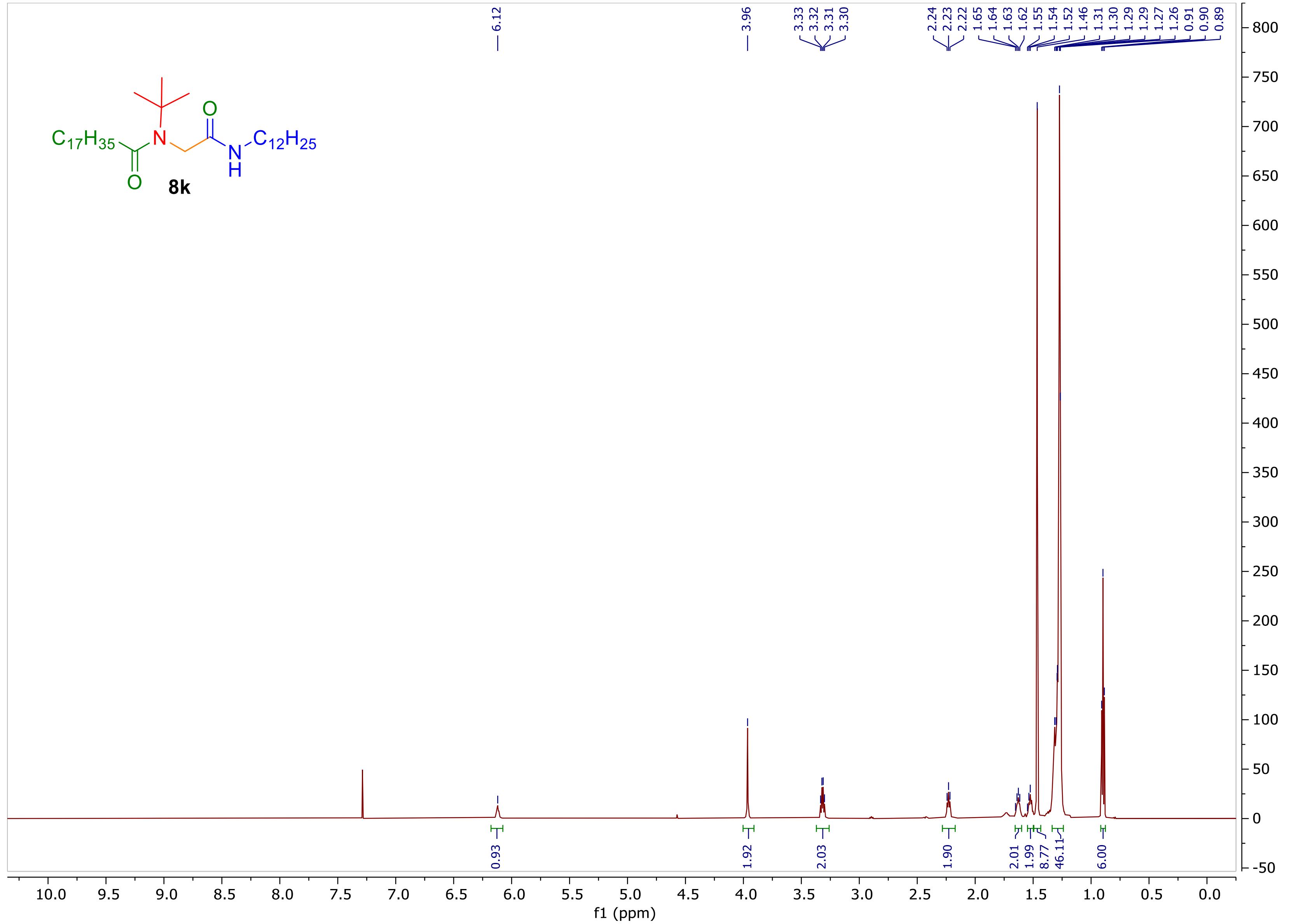

Figure S37. ^{1}H NMR (600 MHz, $CDCl_3$) Spectrum of compound **8k**.

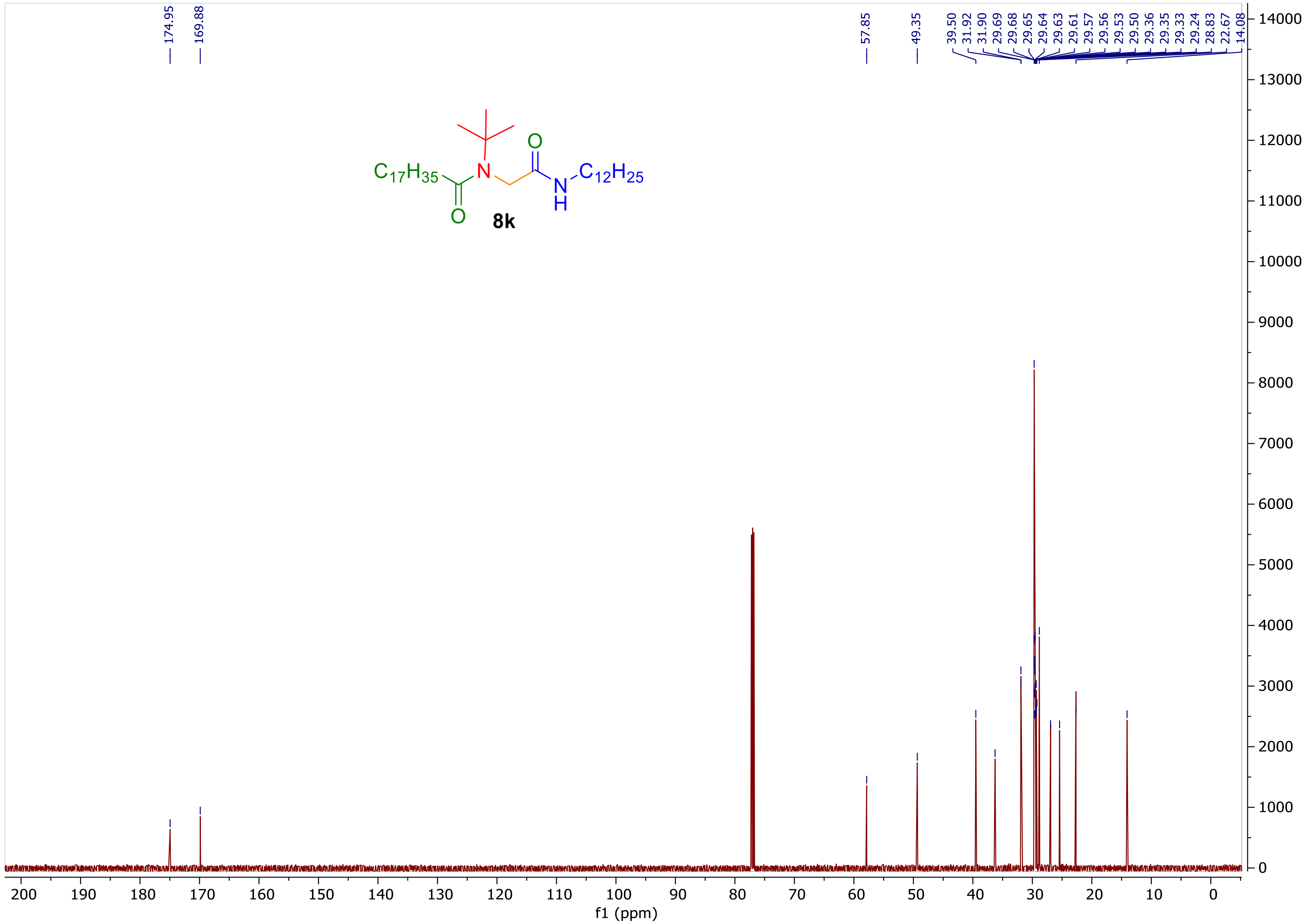

Figure S38. ^{13}C NMR (151 MHz, $CDCl_3$) Spectrum of compound **8k**.

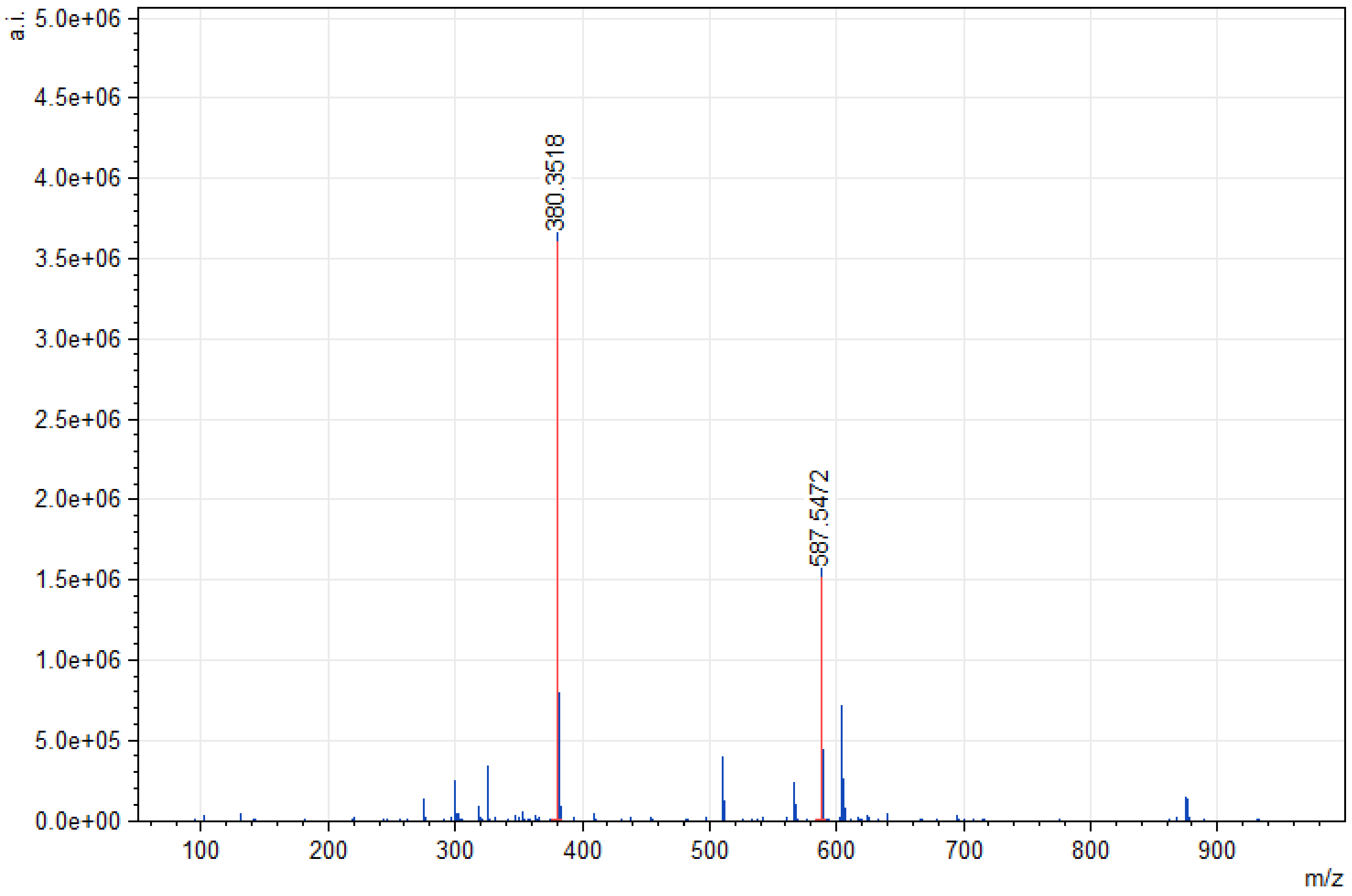

Figure S39. HRMS of compound **8k**.

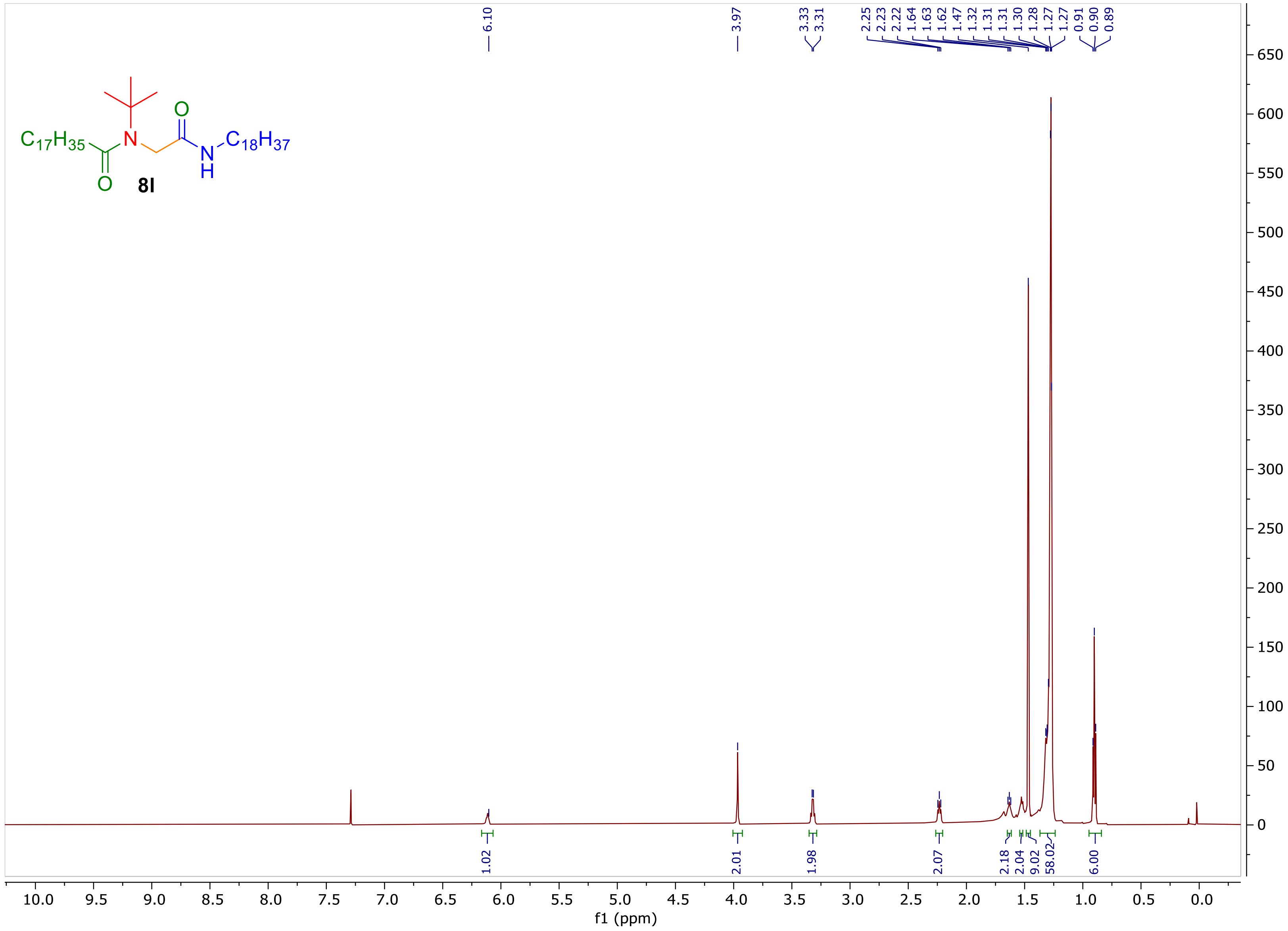

Figure S40. ^{1}H NMR (600 MHz, $CDCl_3$) Spectrum of compound **8l**.

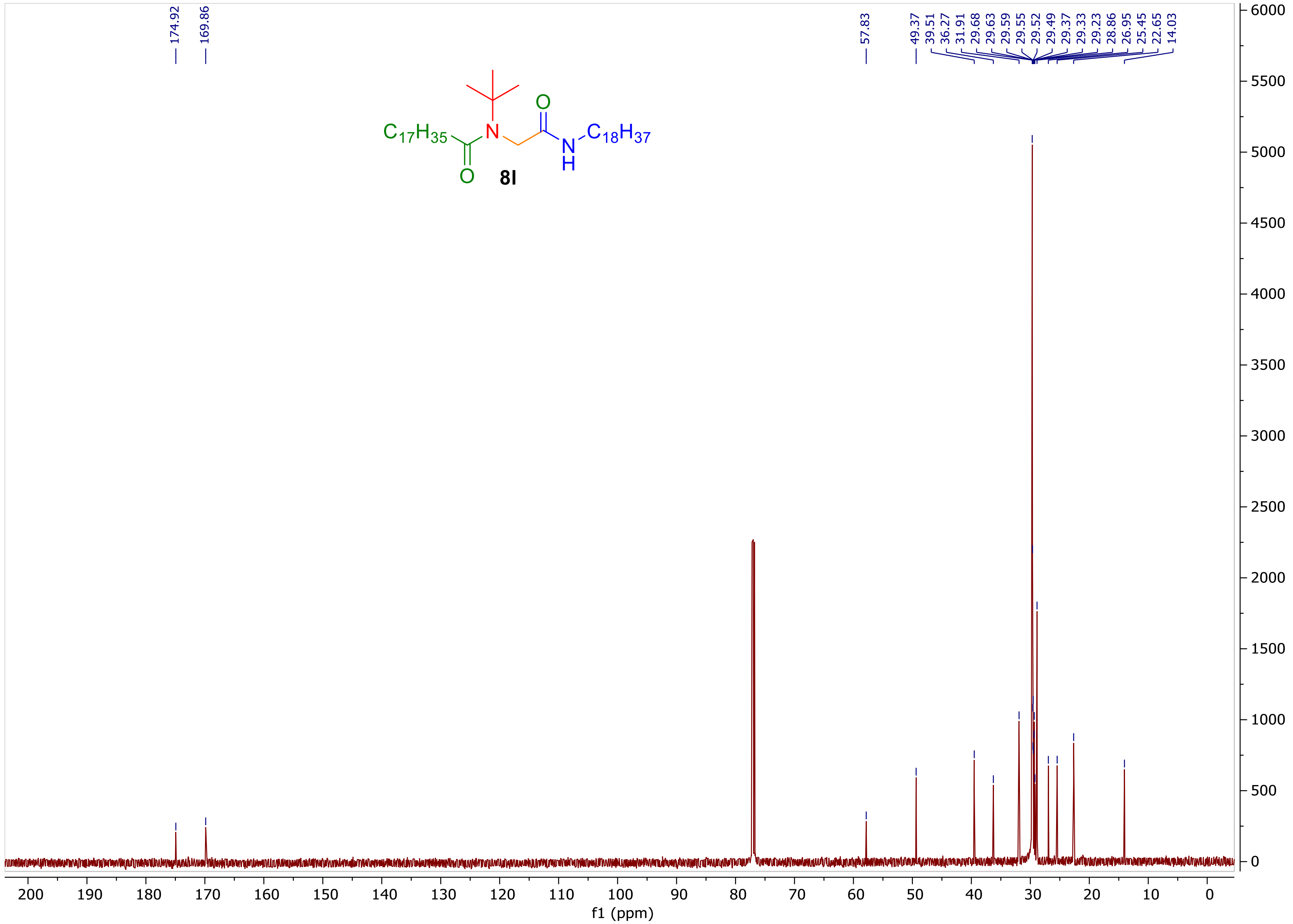

Figure S41. ^{13}C NMR (151 MHz, $CDCl_3$) Spectrum of compound **8l**.

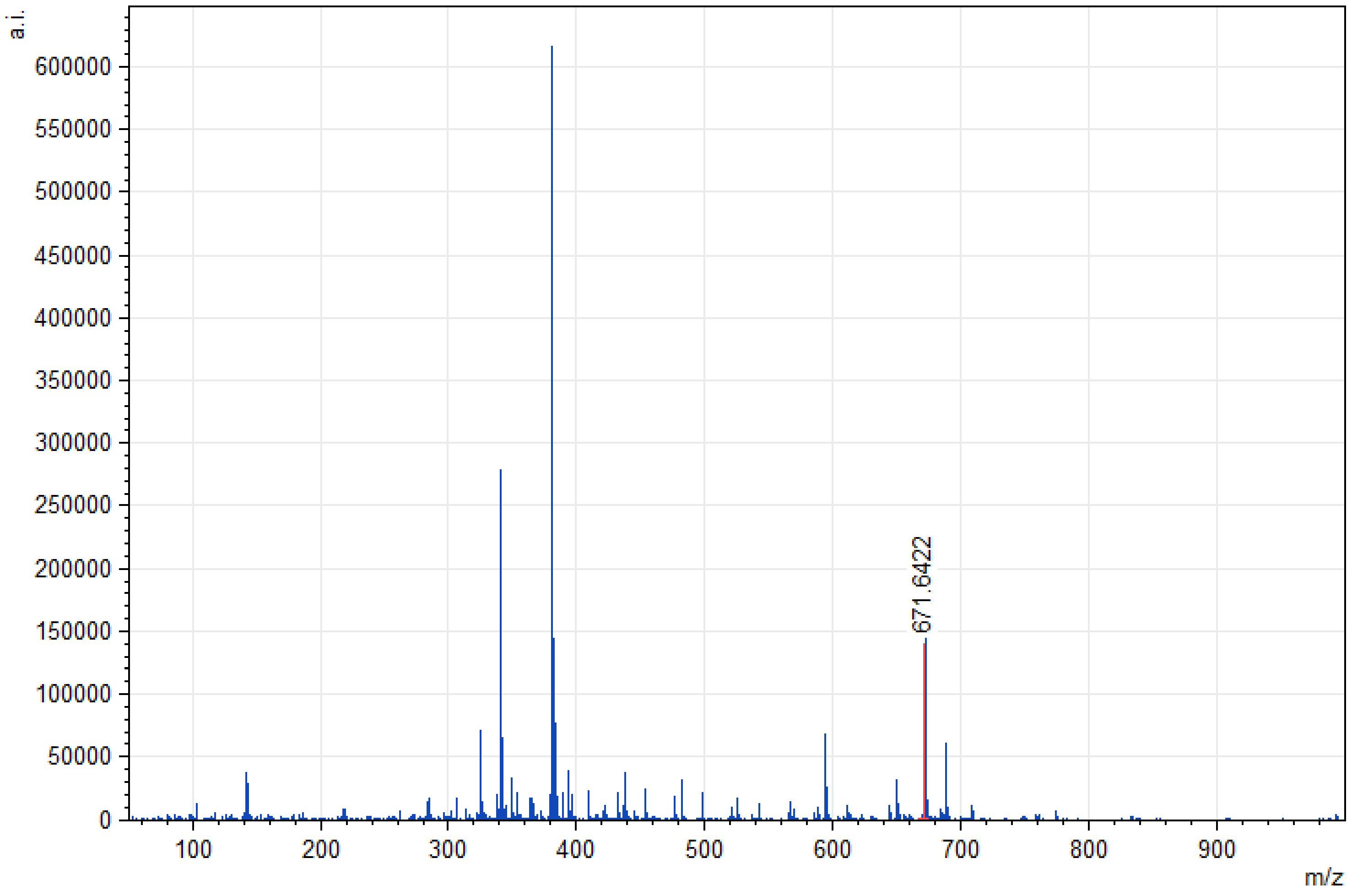

Figure S42. HRMS of compound **8l**.

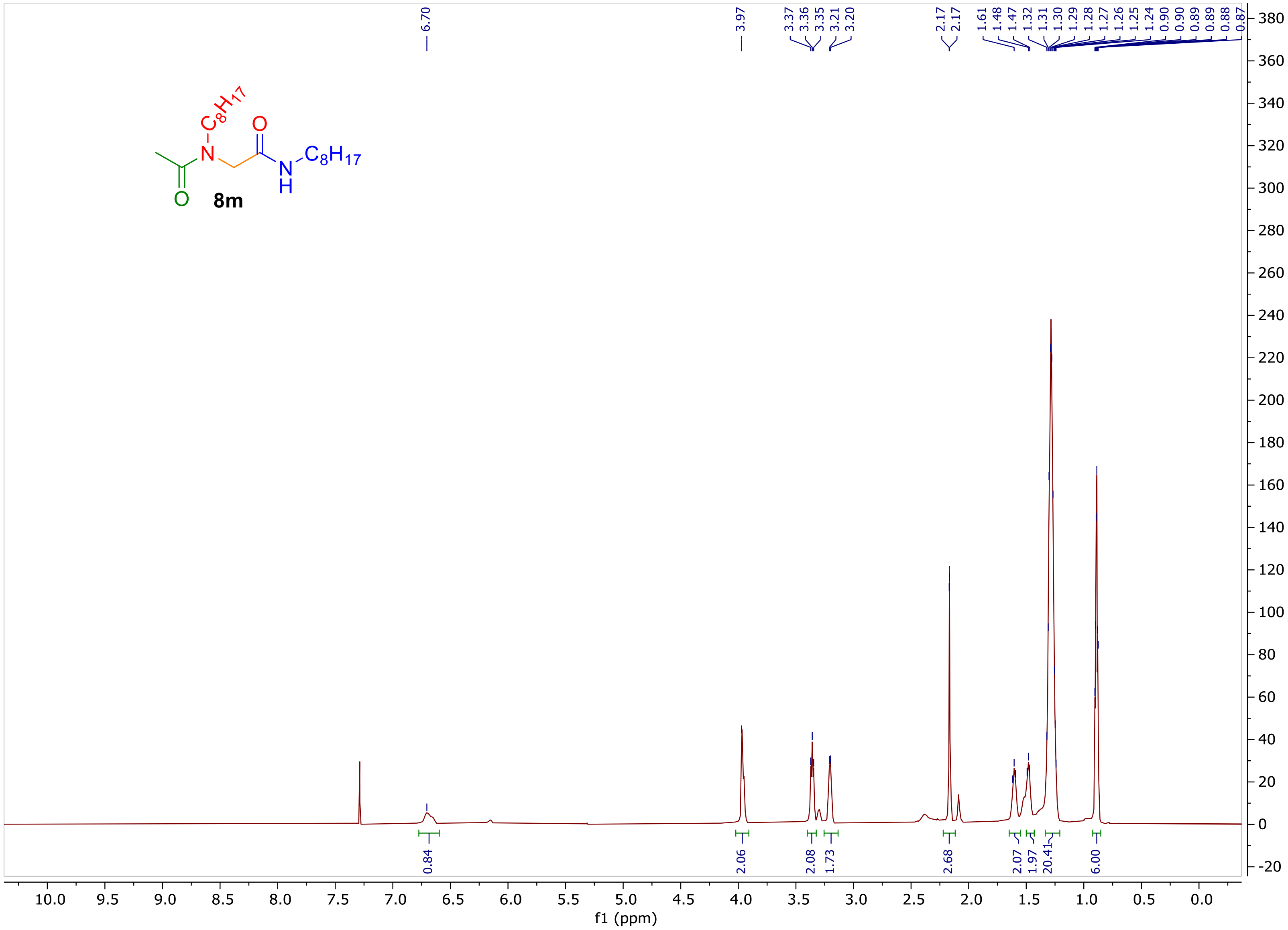

Figure S43. ^{1}H NMR (600 MHz, $CDCl_3$) Spectrum of compound **8m**.

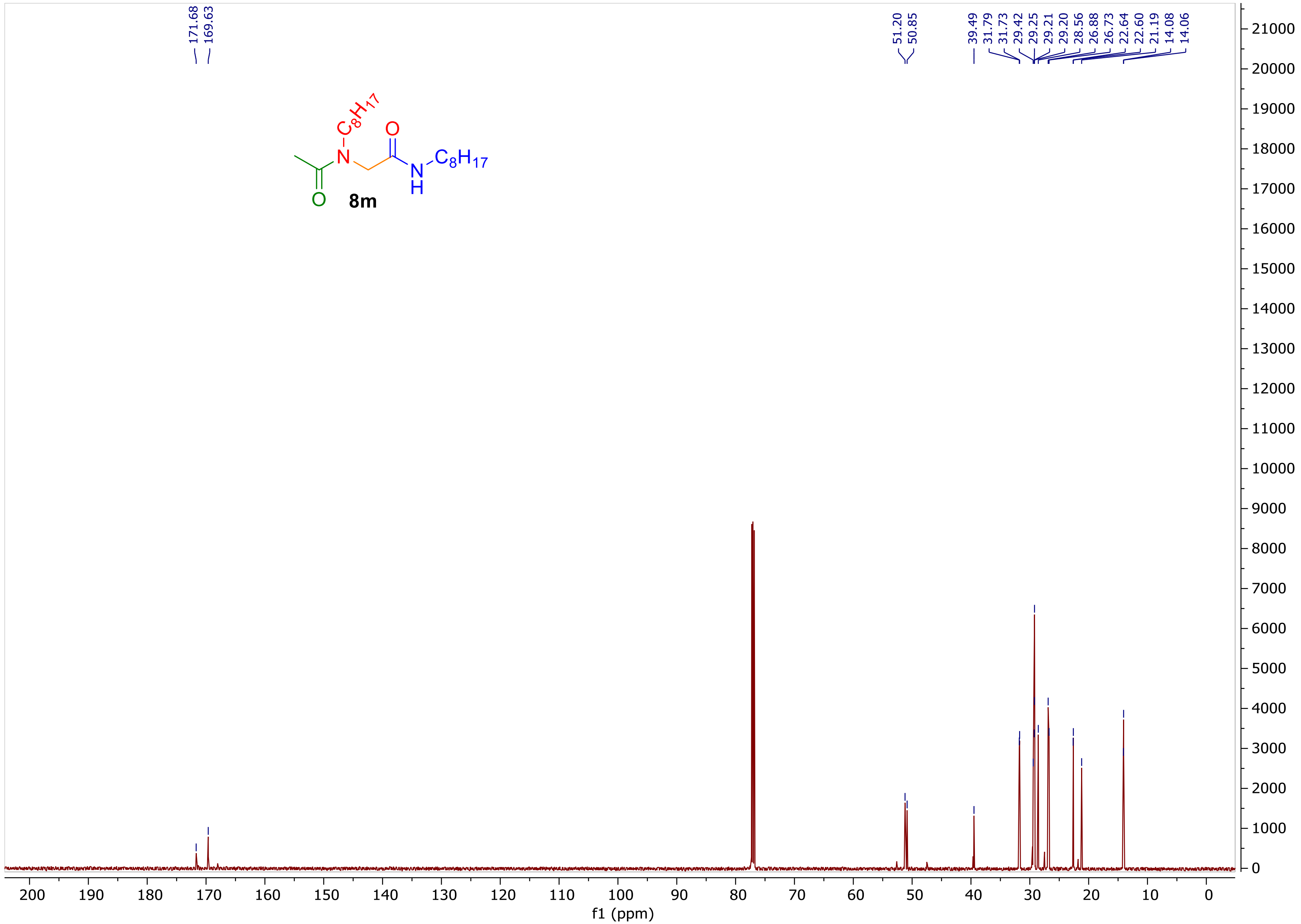

Figure S44. ^{13}C NMR (151 MHz, $CDCl_3$) Spectrum of compound **8m**.

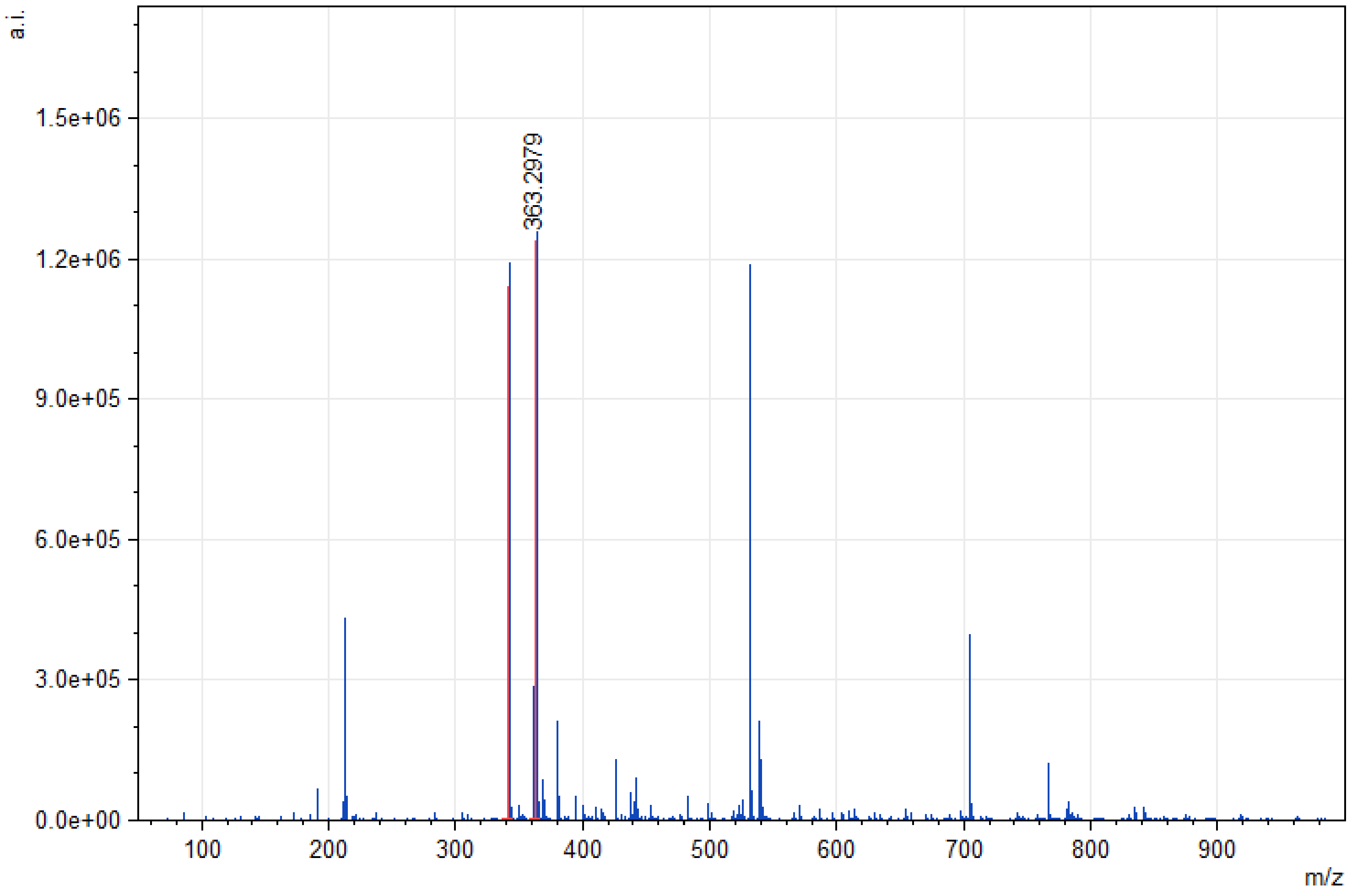

Figure S45. HRMS of compound **8m.**

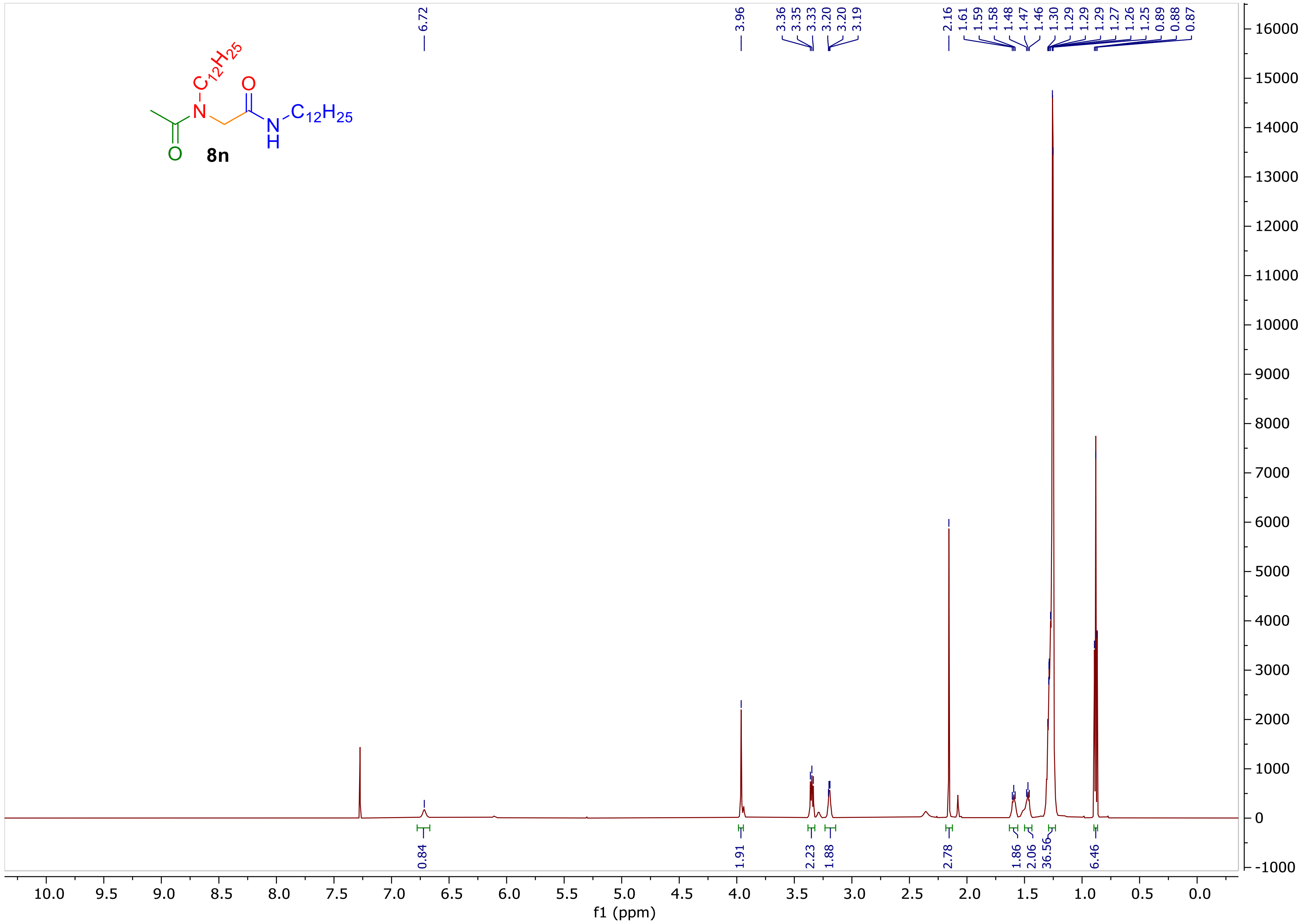

Figure S46. ^{1}H NMR (600 MHz, $CDCl_3$) Spectrum of compound **8n**.

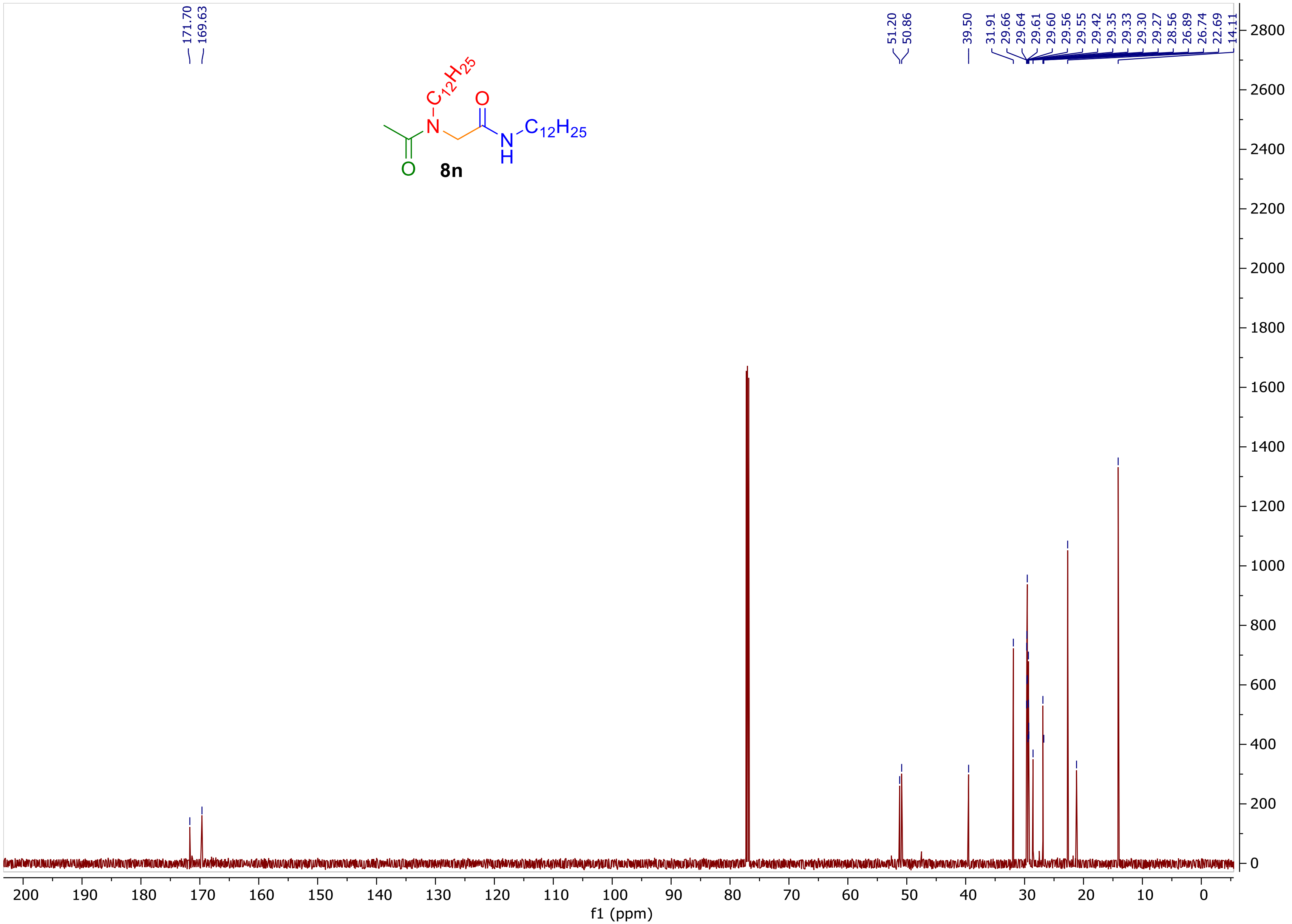

Figure S47. ^{13}C NMR (151 MHz, $CDCl_3$) Spectrum of compound **8n**.

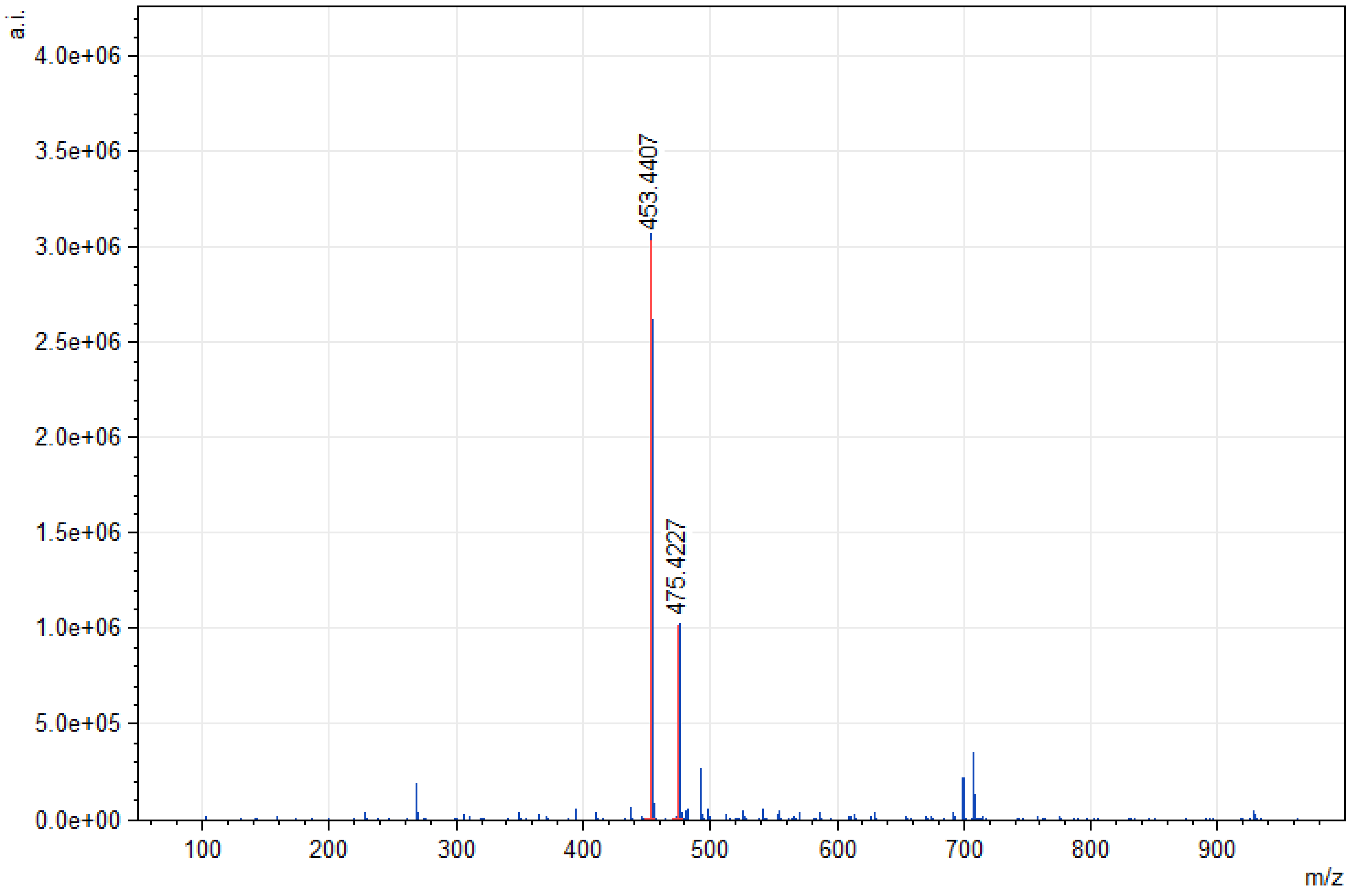

Figure S48. HRMS of compound **8n**.

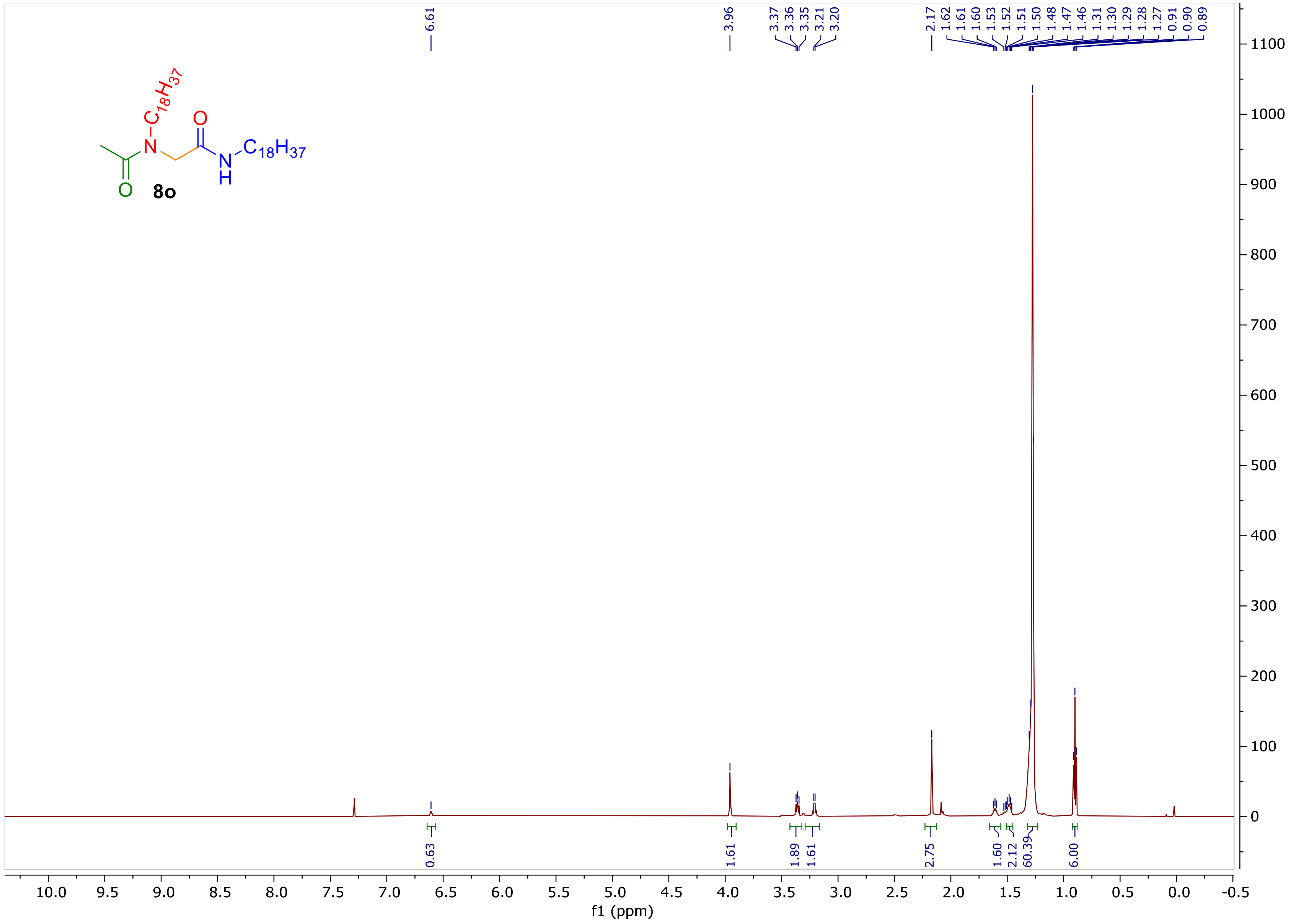

g

Figure S49. ^{1}H NMR (600 MHz, $CDCl_3$) Spectrum of compound **8o**.

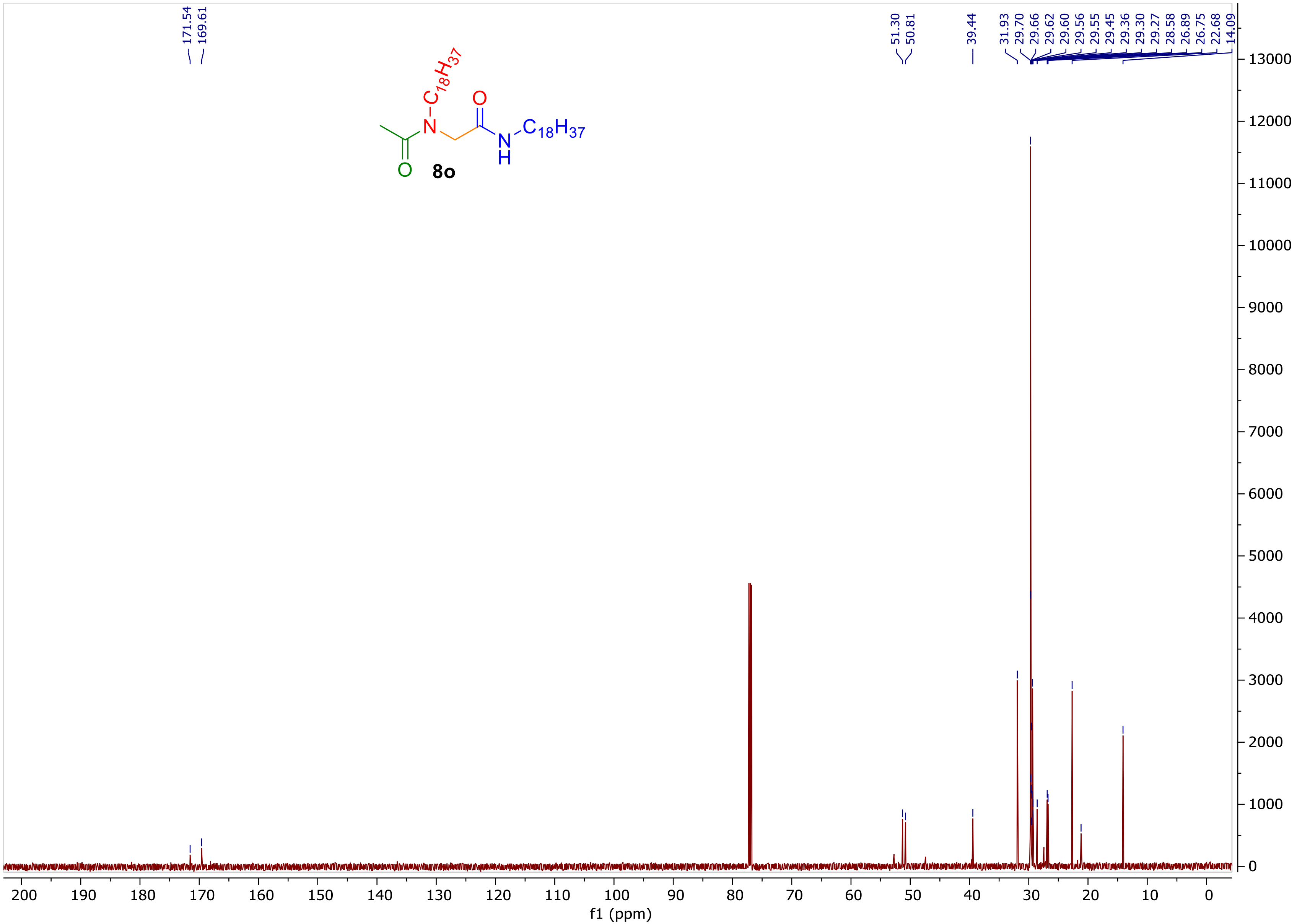

Figure S50. ^{13}C NMR (151 MHz, $CDCl_3$) Spectrum of compound **8o**.

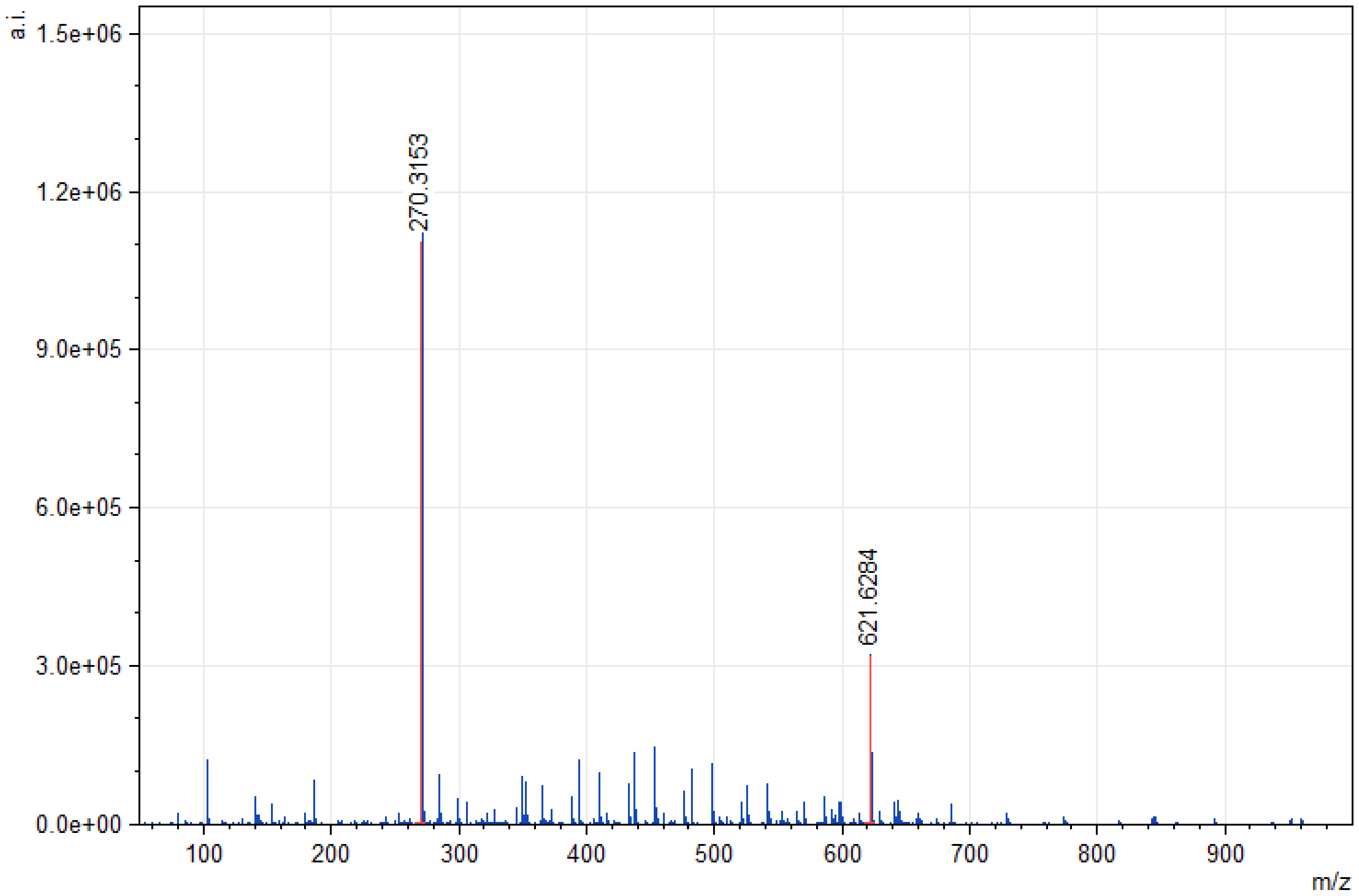

Figure S51. HRMS of compound **8o**.

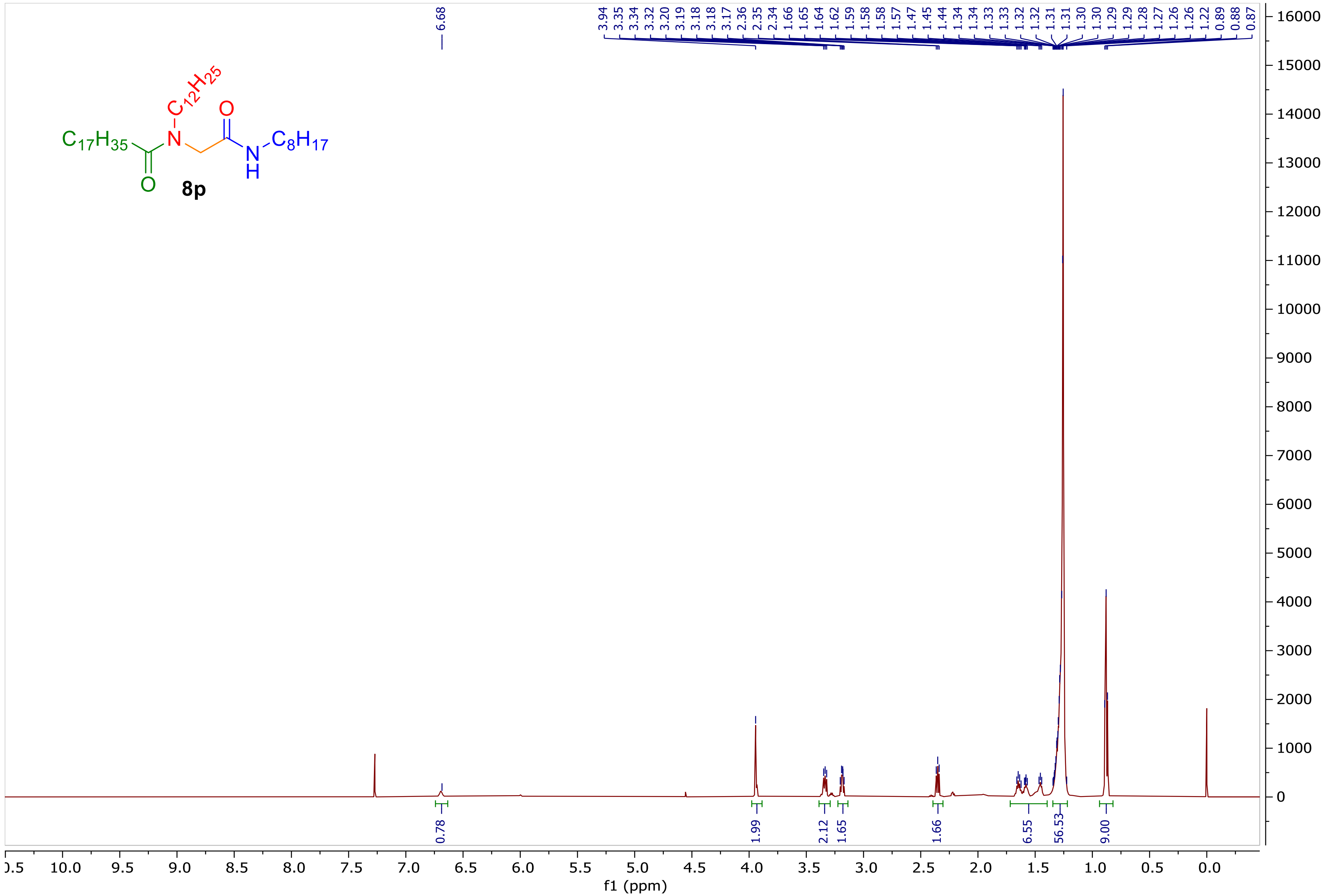

Figure S52. ^{1}H NMR (600 MHz, $CDCl_3$) Spectrum of compound **8p**.

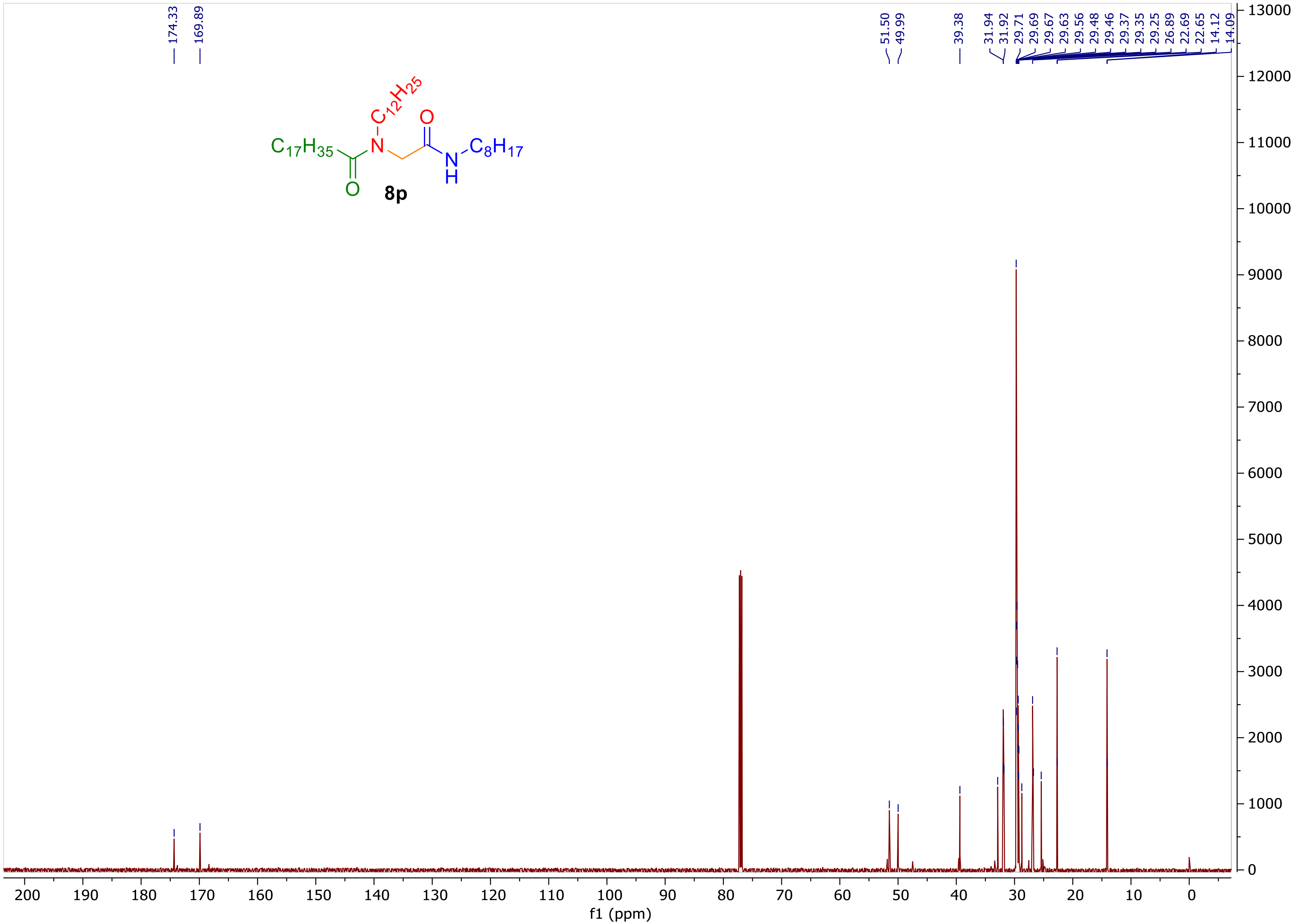

Figure S53. ^{13}C NMR (151 MHz, $CDCl_3$) Spectrum of compound **8p**.

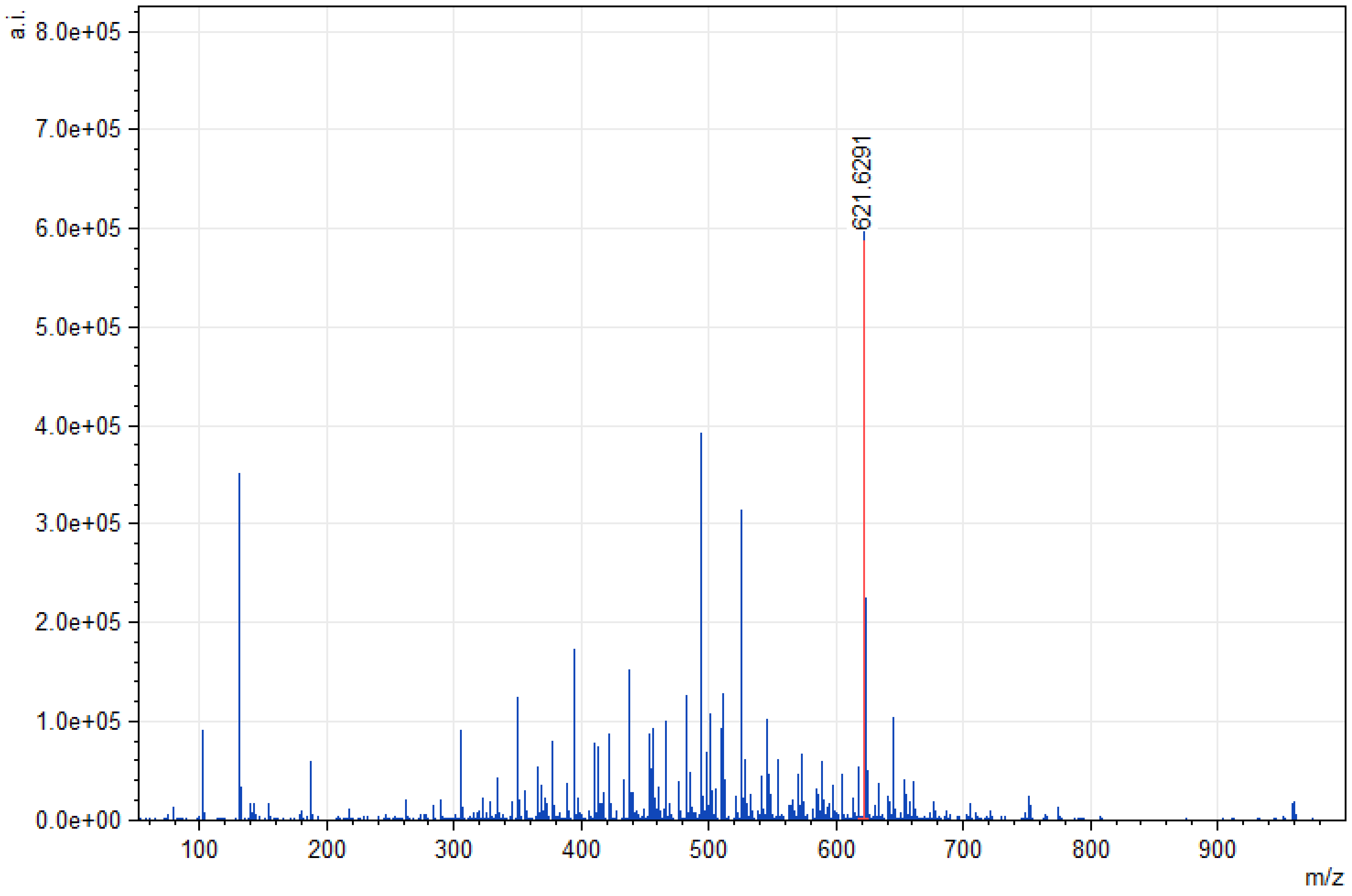

Figure S54. HRMS of compound **8p**.

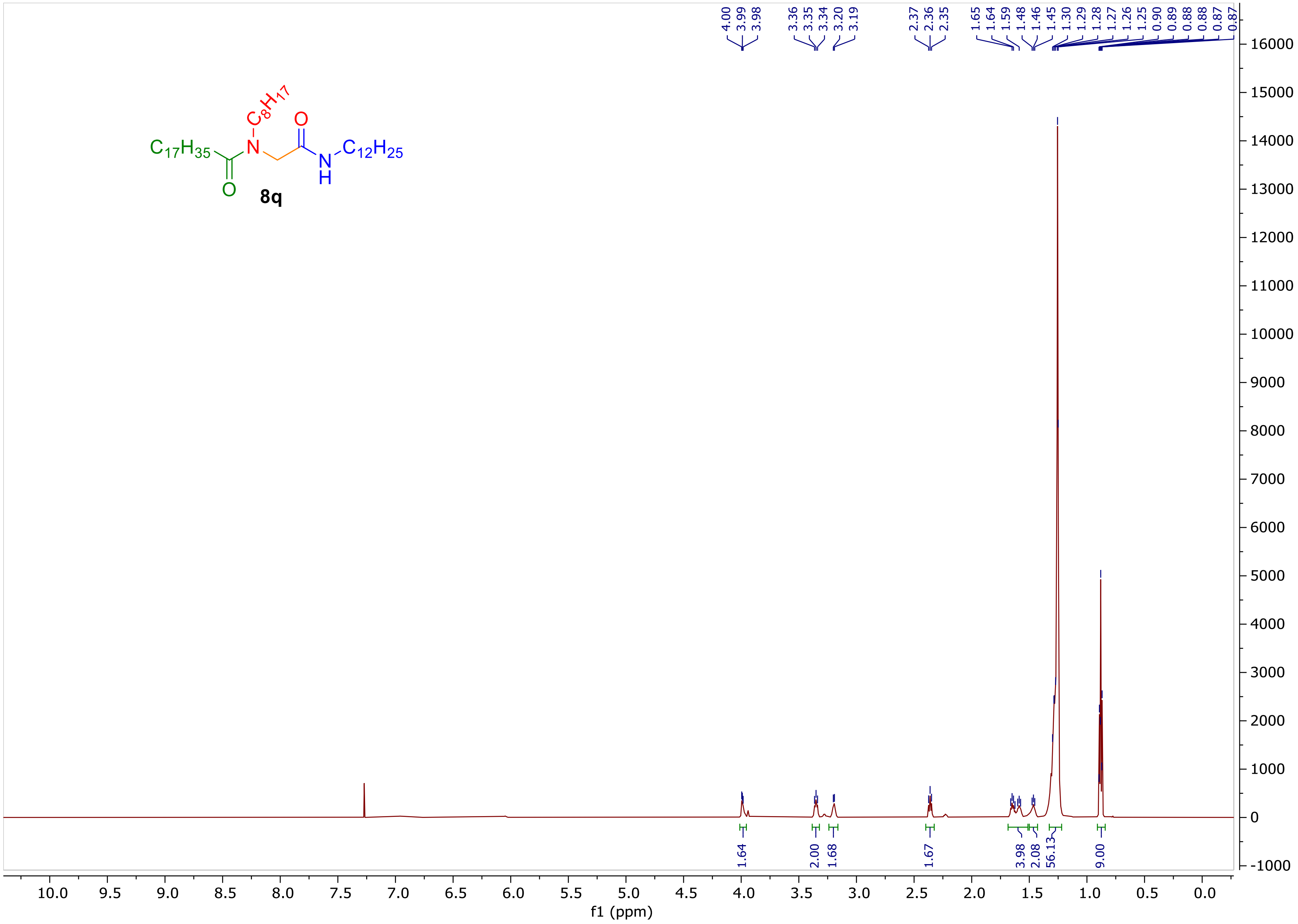

Figure S55. ^{1}H NMR (600 MHz, $CDCl_3$) Spectrum of compound **8q.**

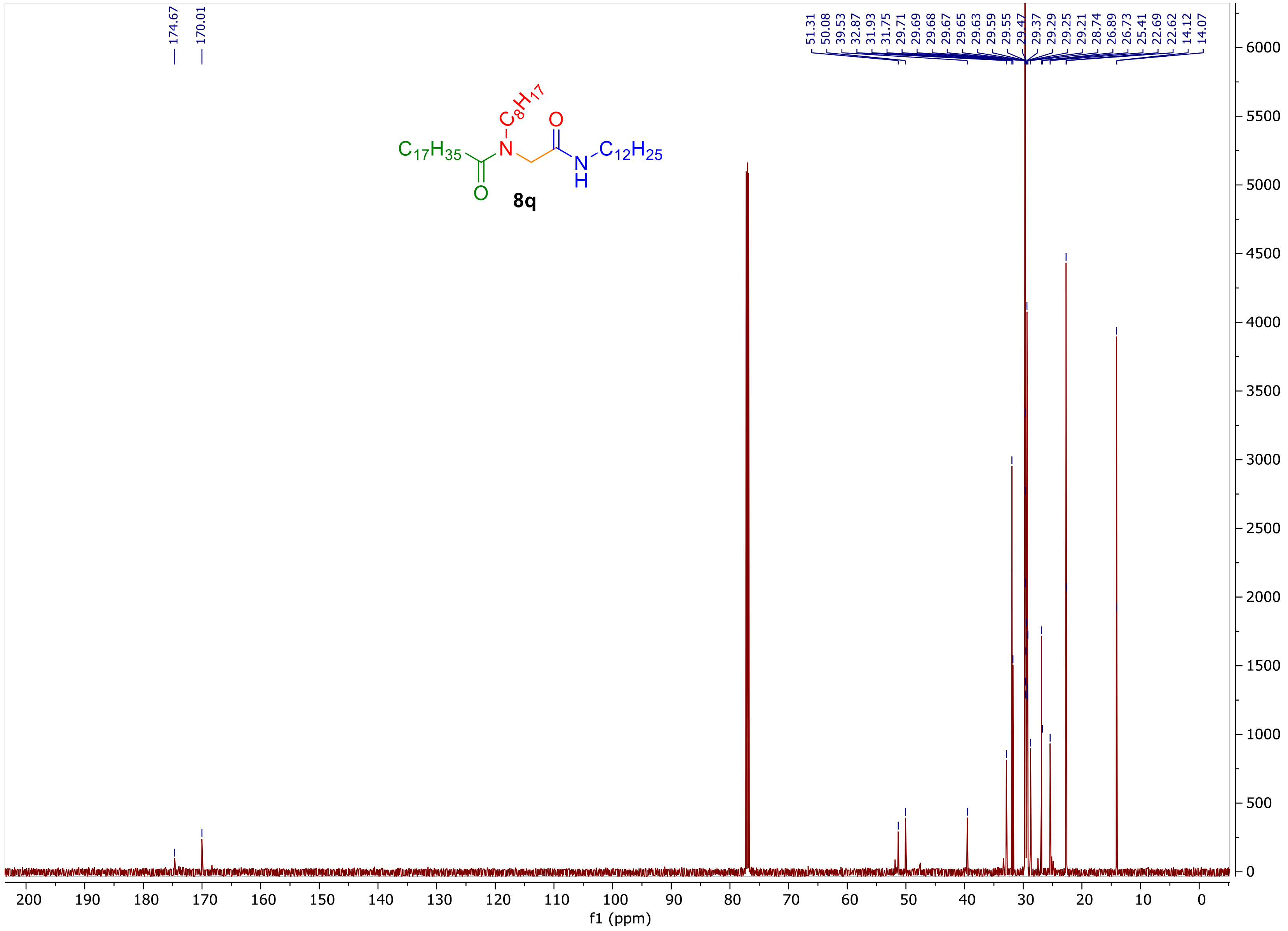

Figure S56. ^{13}C NMR (151 MHz, $CDCl_3$) Spectrum of compound **8q**.

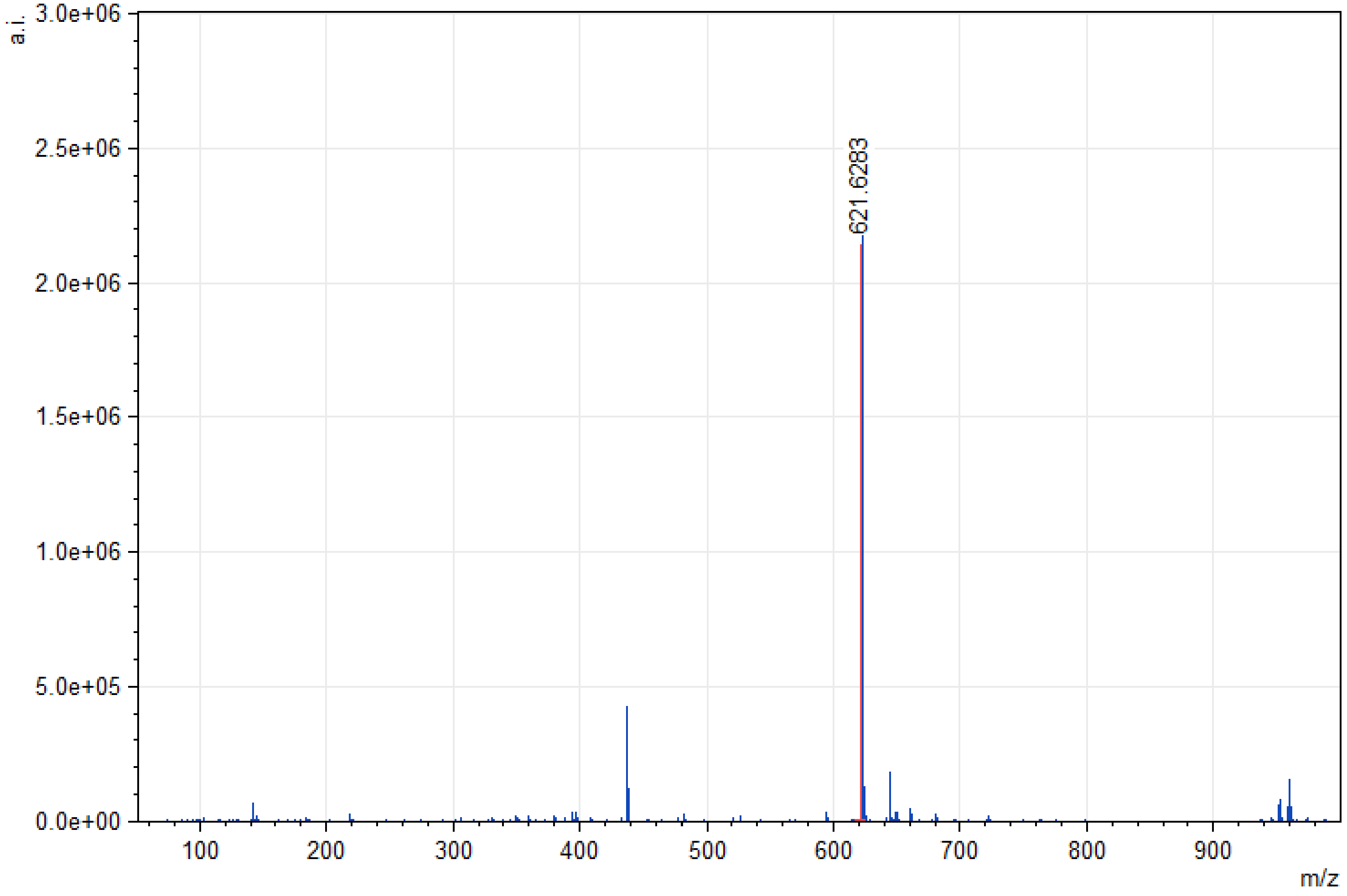

Figure S57. HRMS of compound **8q**.

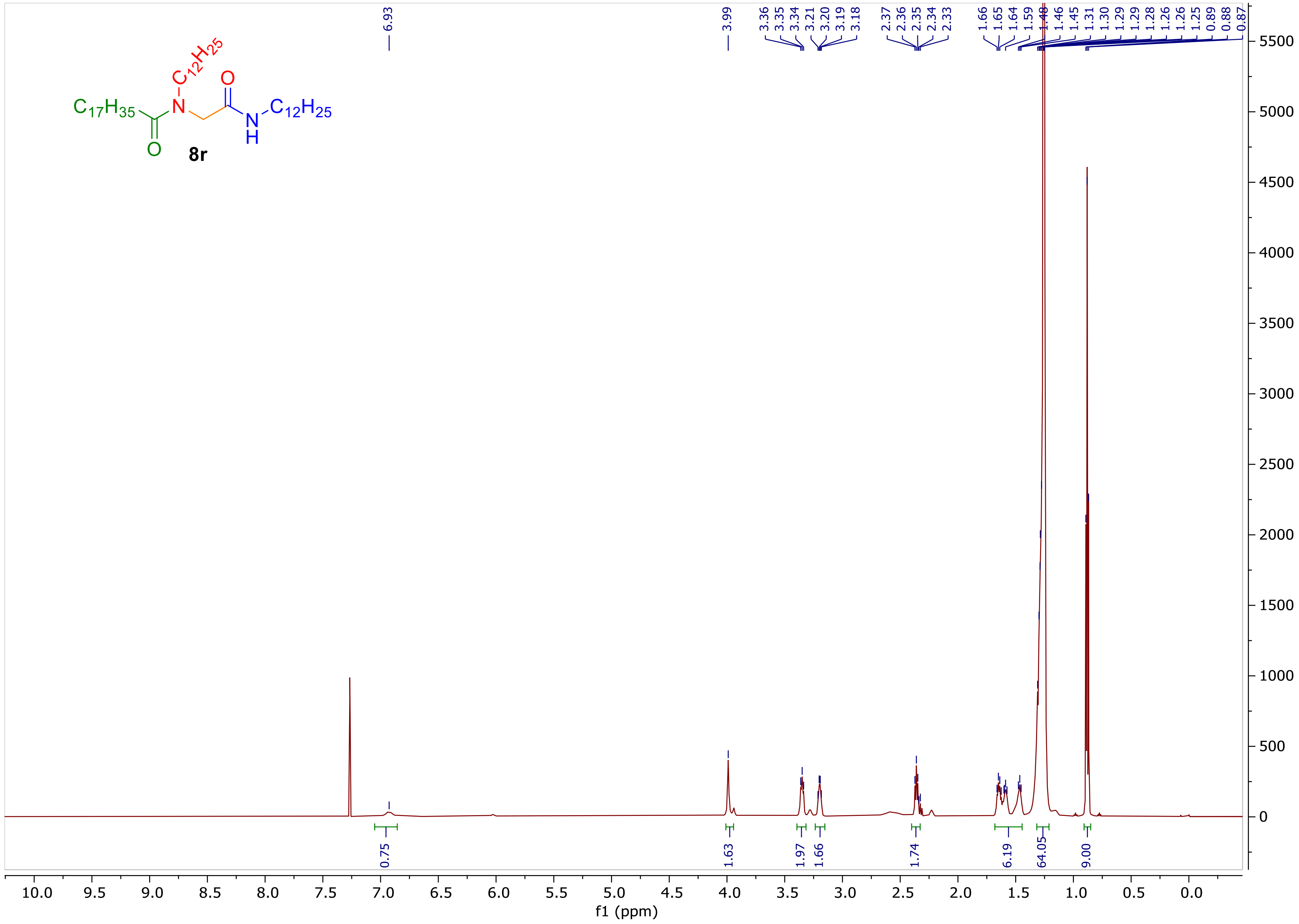

Figure S58. ^{1}H NMR (600 MHz, $CDCl_3$) Spectrum of compound **8r.**

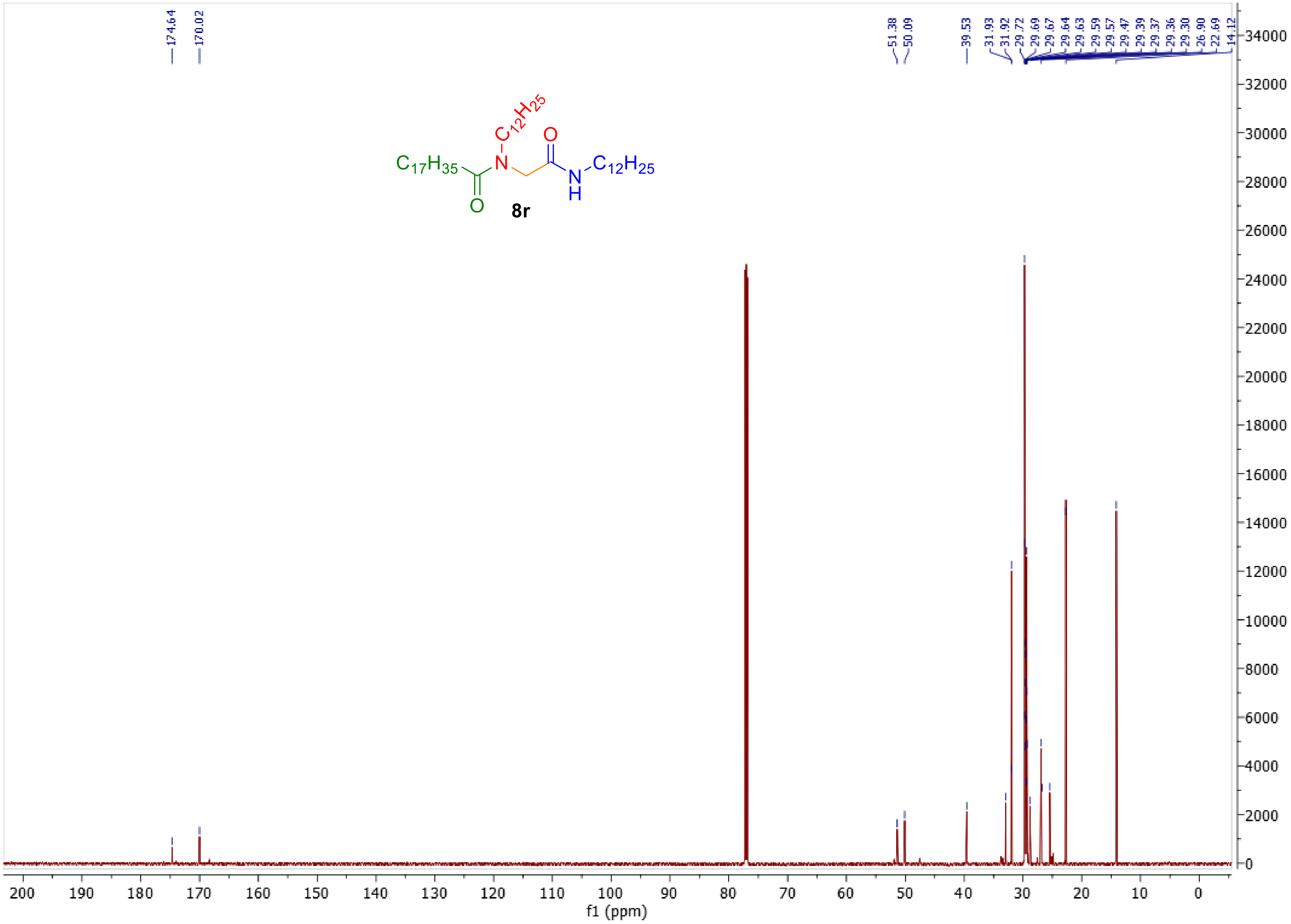

Figure S59. ^{13}C NMR (151 MHz, $CDCl_3$) Spectrum of compound **8r**.

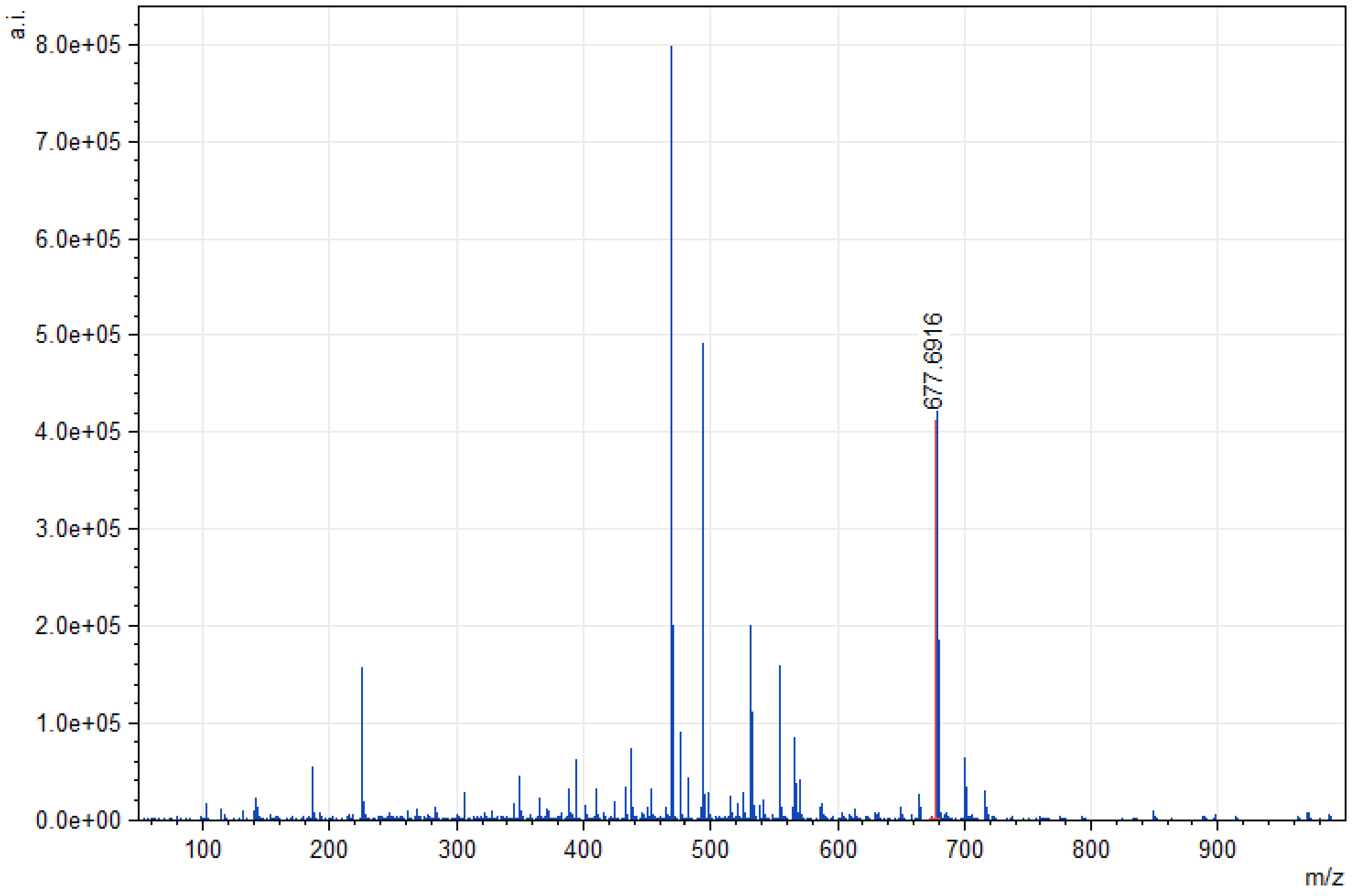

Figure S60. HRMS of compound **8r**.

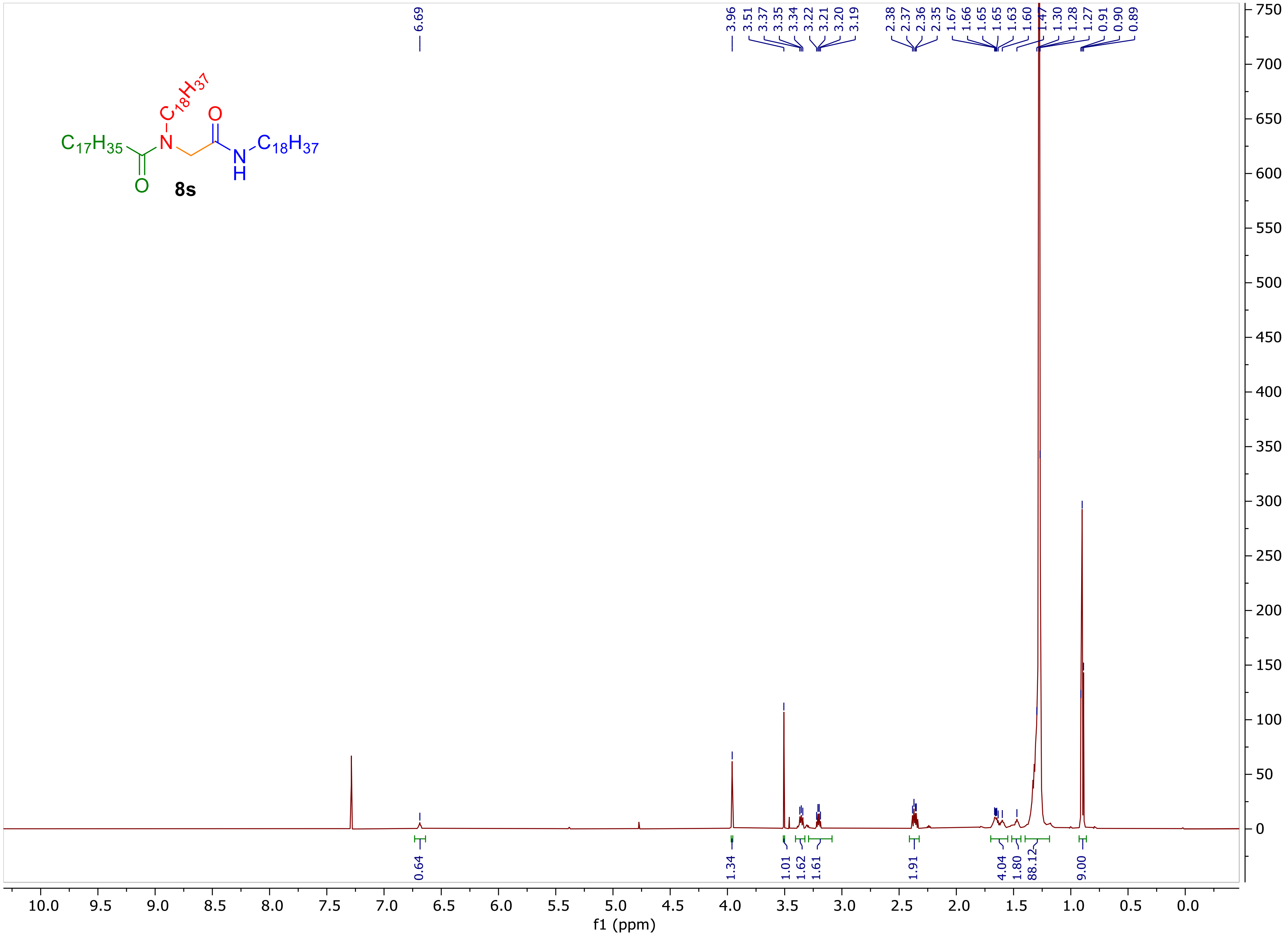

Figure S61. ^{1}H NMR (600 MHz, $CDCl_3$) Spectrum of compound **8s.**

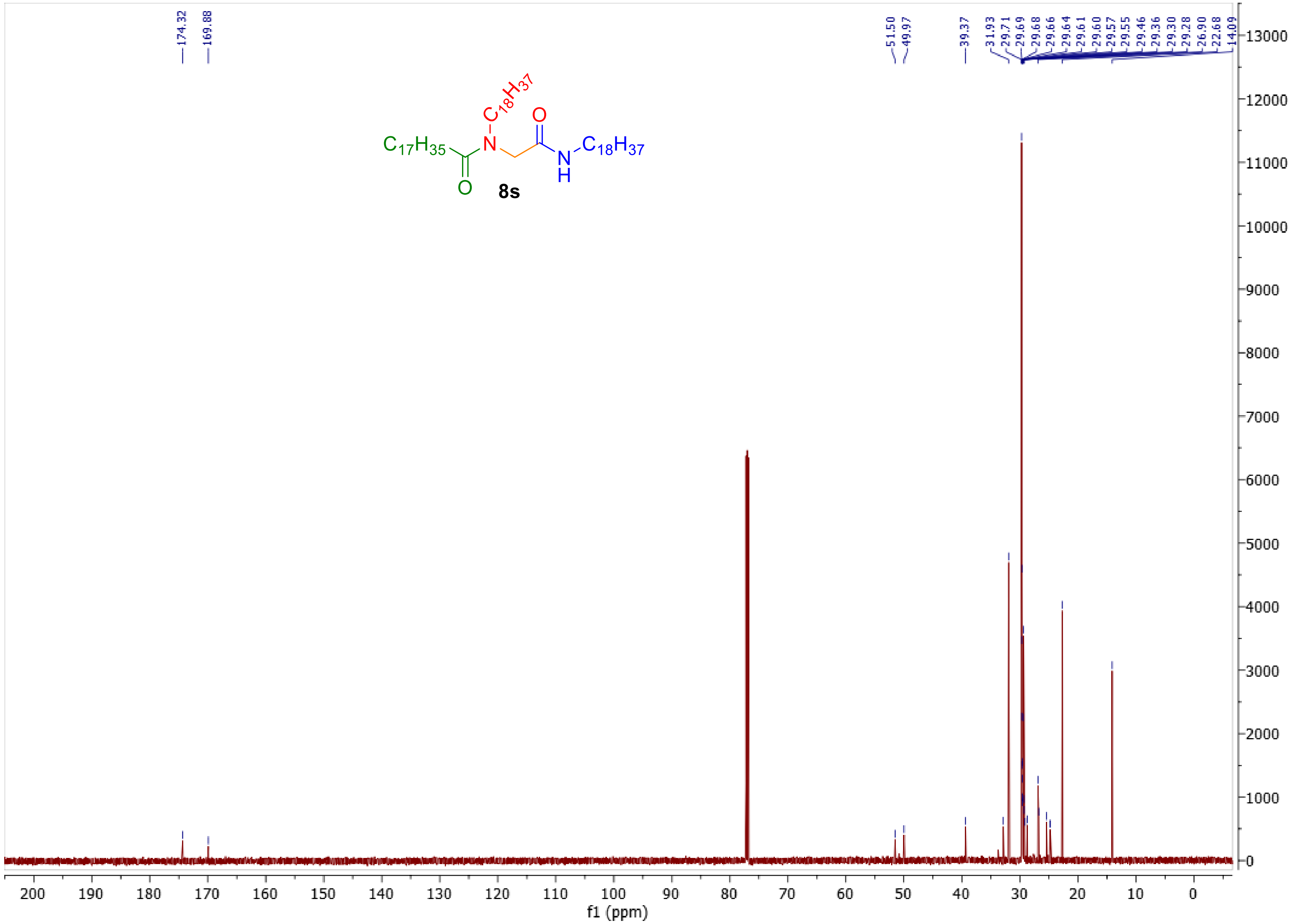

Figure S62. ^{13}C NMR (151 MHz, $CDCl_3$) Spectrum of compound **8s**.

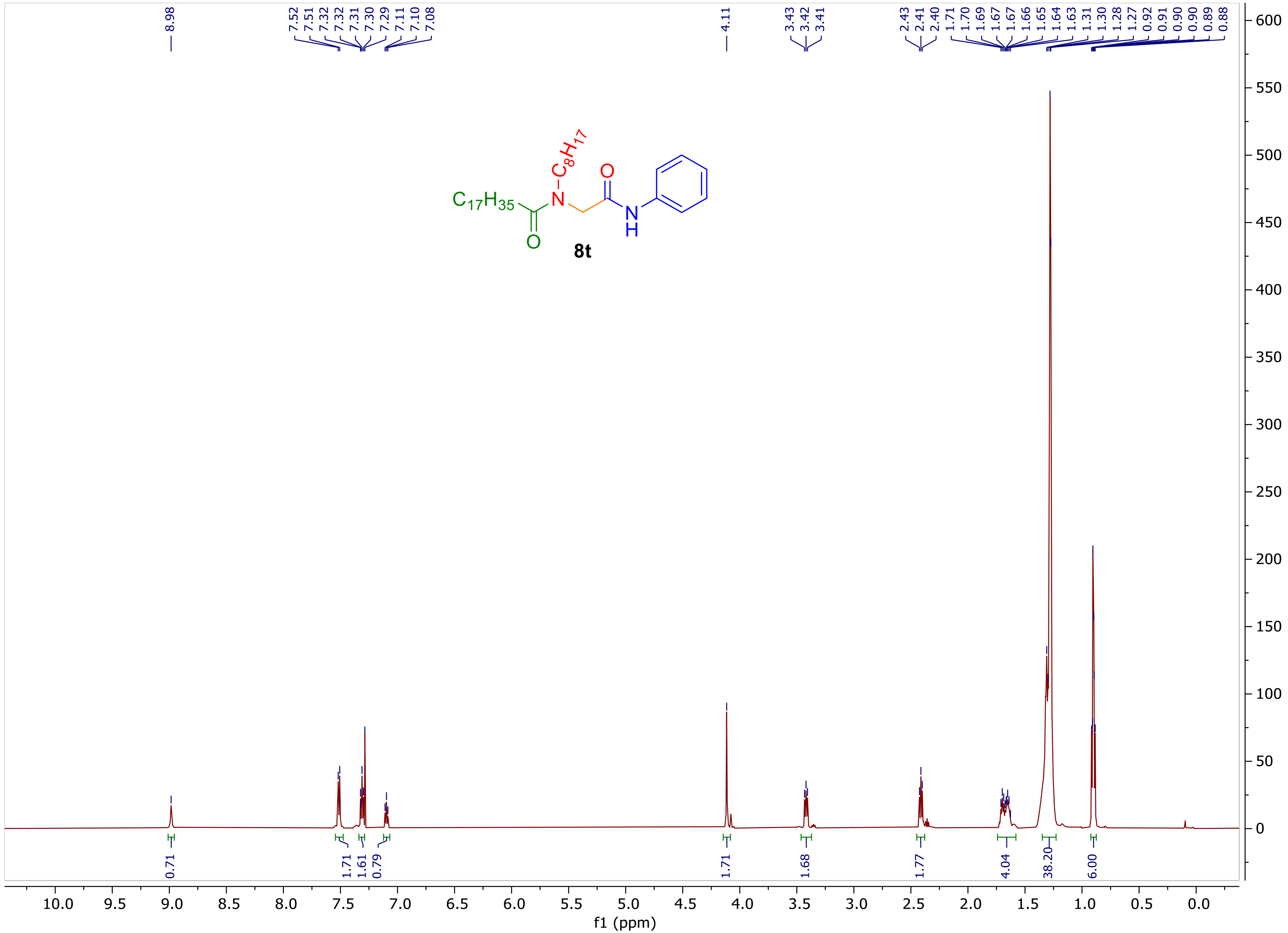

Figure S63. ¹H NMR (600 MHz, $CDCl_3$) Spectrum of compound **8t.**

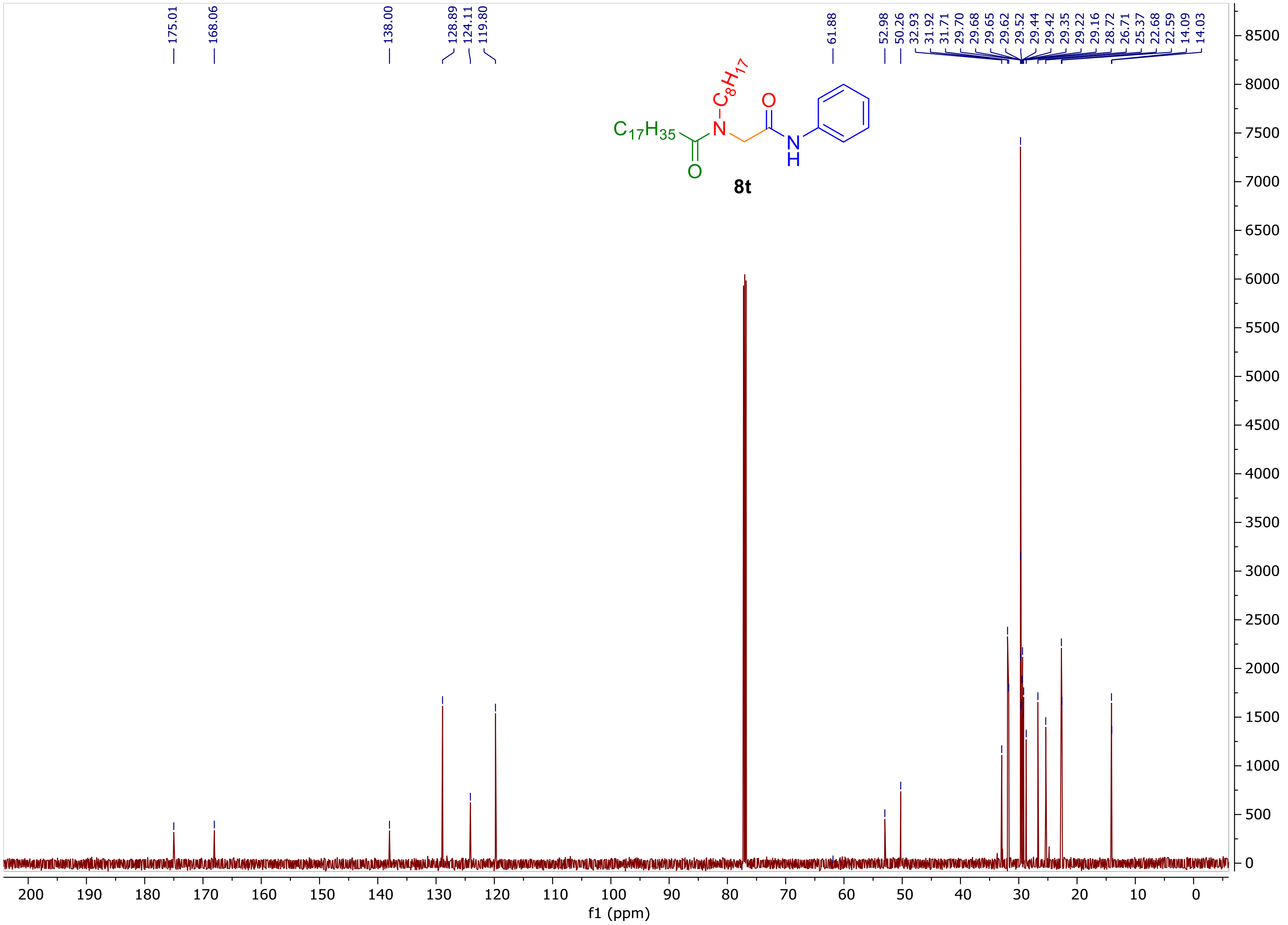

Figure S64. ^{13}C NMR (151 MHz, $CDCl_3$) Spectrum of compound **8t.**

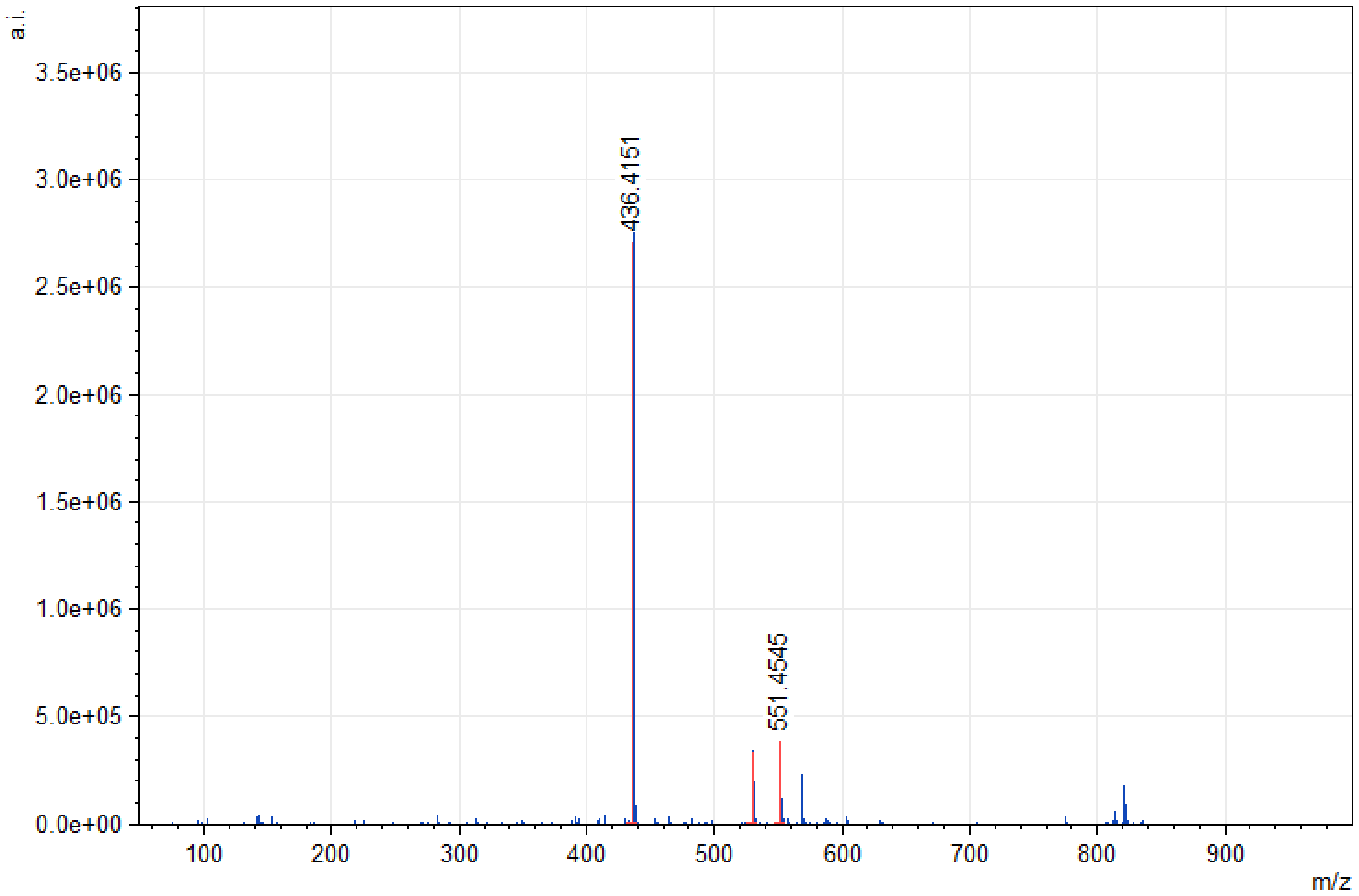

Figure S65. HRMS of compound **8t**.

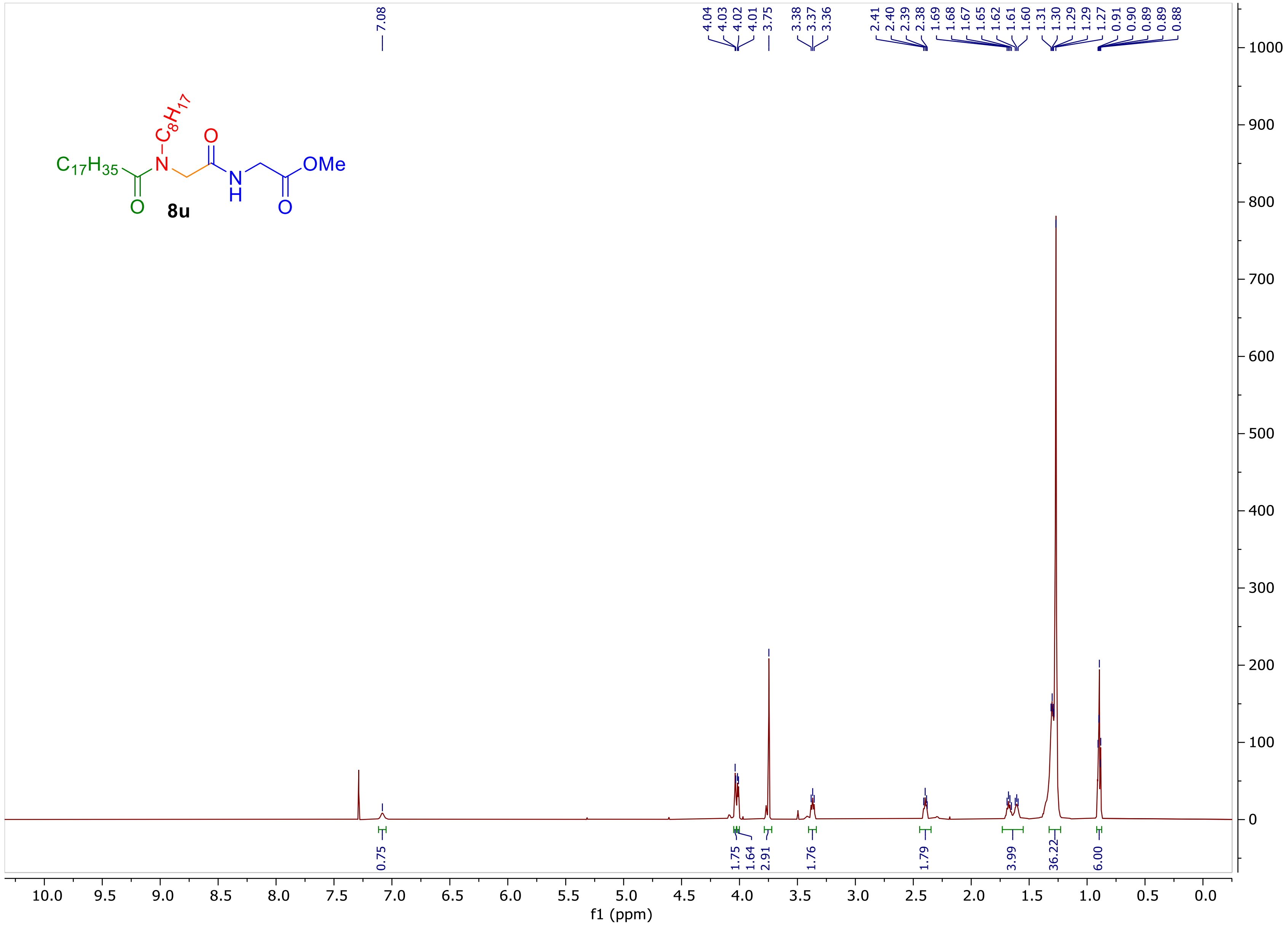

Figure S66. ^{1}H NMR (600 MHz, $CDCl_3$) Spectrum of compound **8u.**

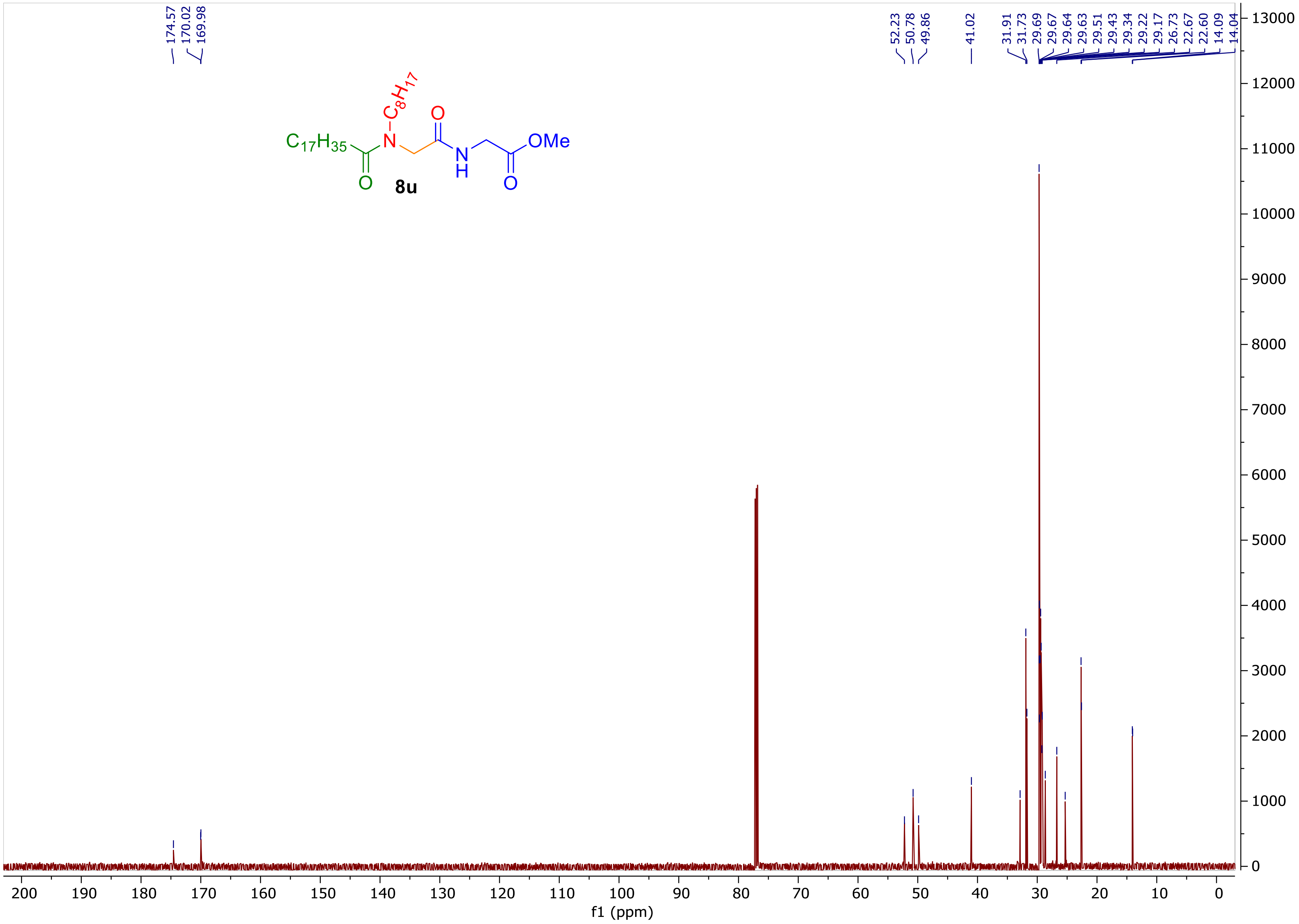

Figure S67. ^{13}C NMR (151 MHz, $CDCl_3$) Spectrum of compound **8u**.

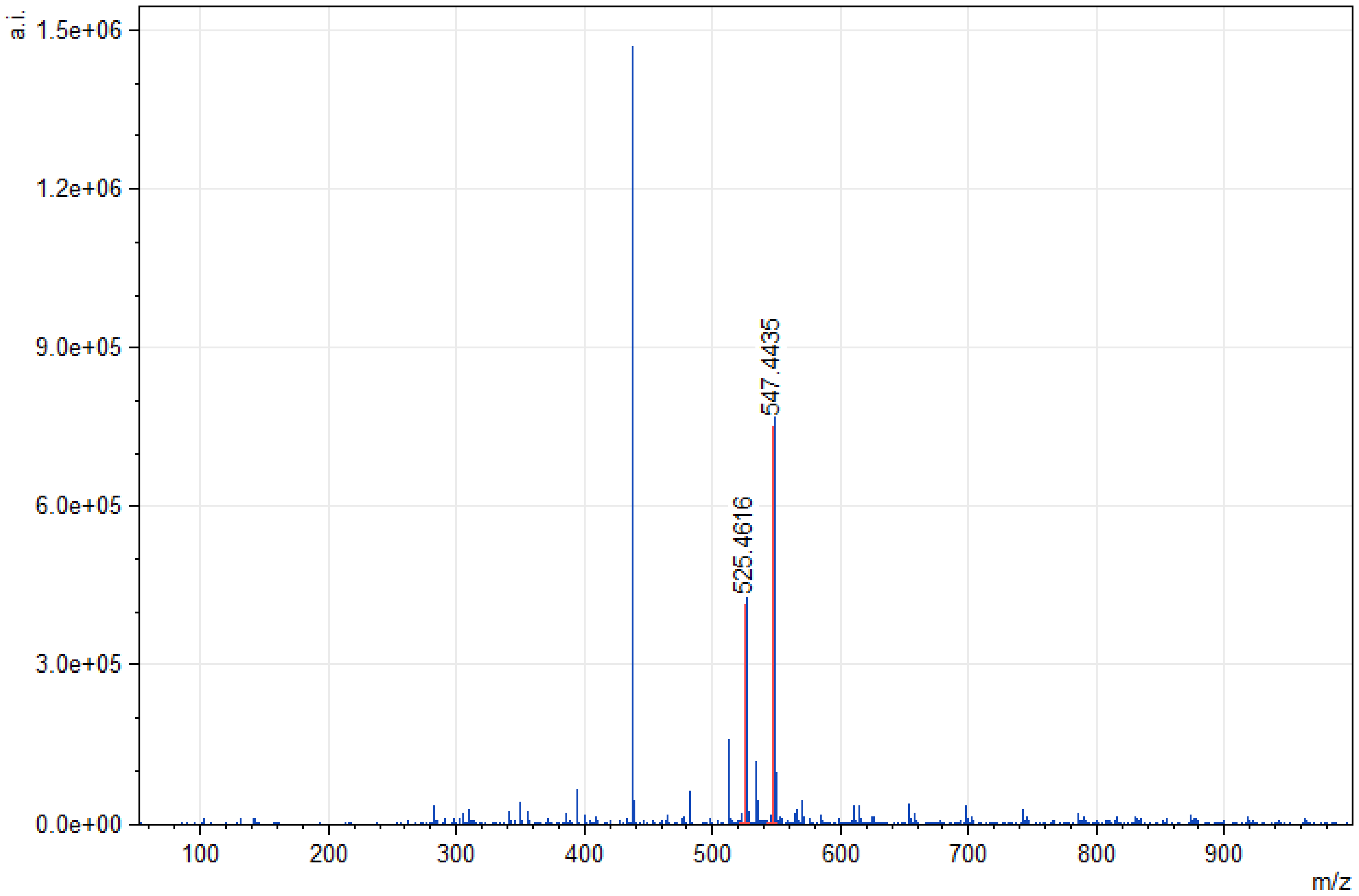

Figure S68. HRMS of compound **8u**.

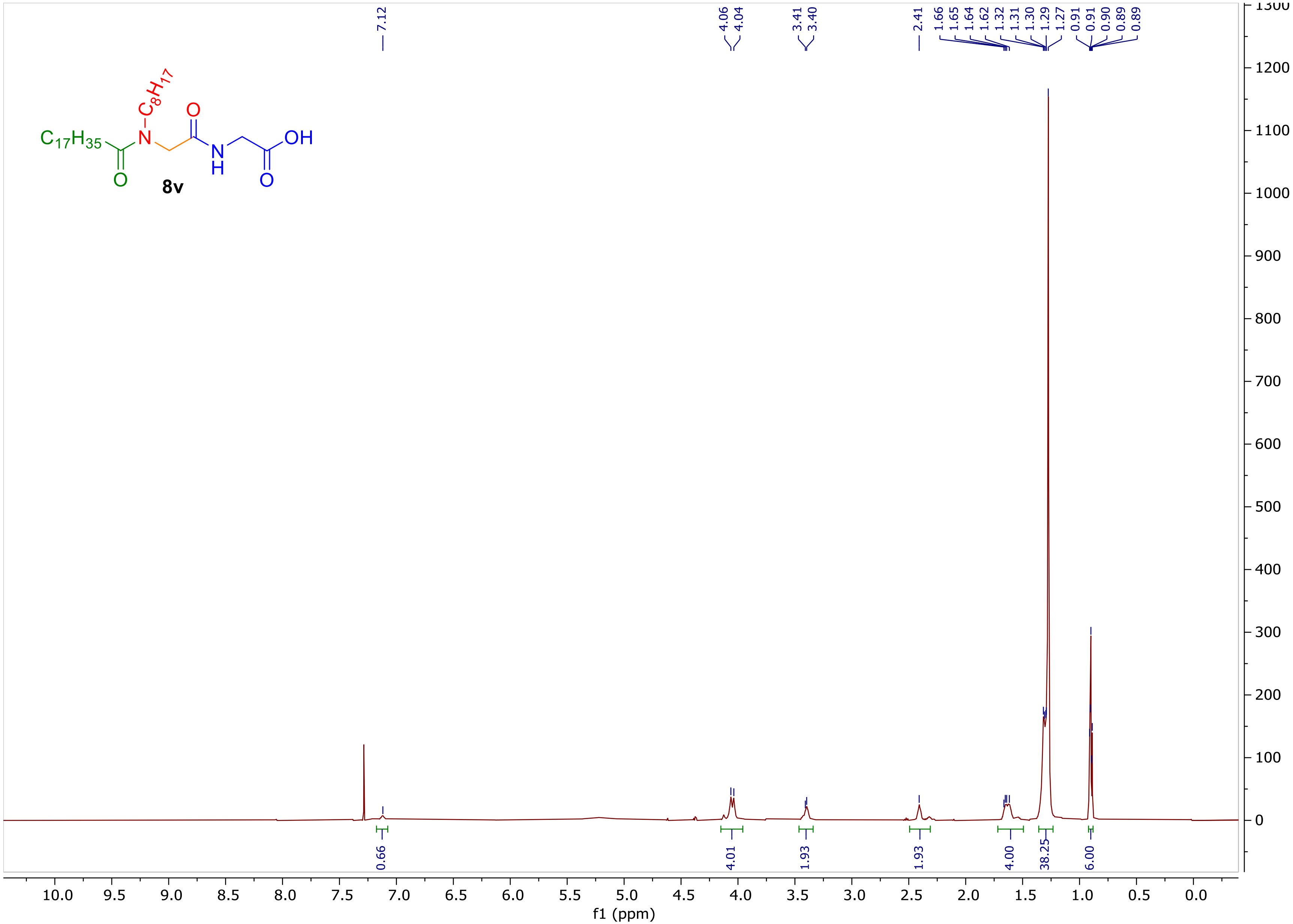

Figure S69. ^{1}H NMR (600 MHz, $CDCl_3$) Spectrum of compound **8v**.

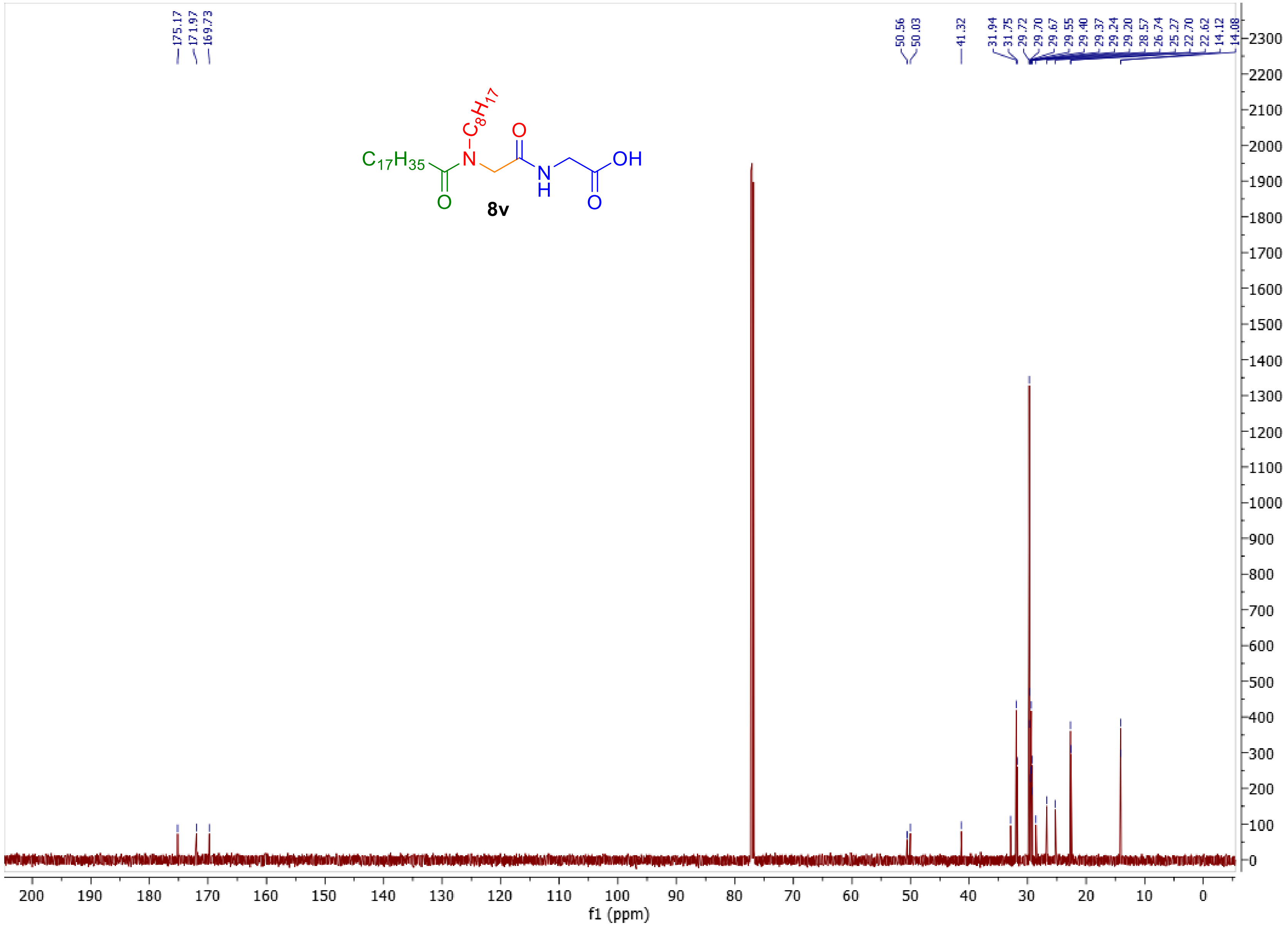

Figure S70. ^{13}C NMR (151 MHz, $CDCl_3$) Spectrum of compound **8v**.

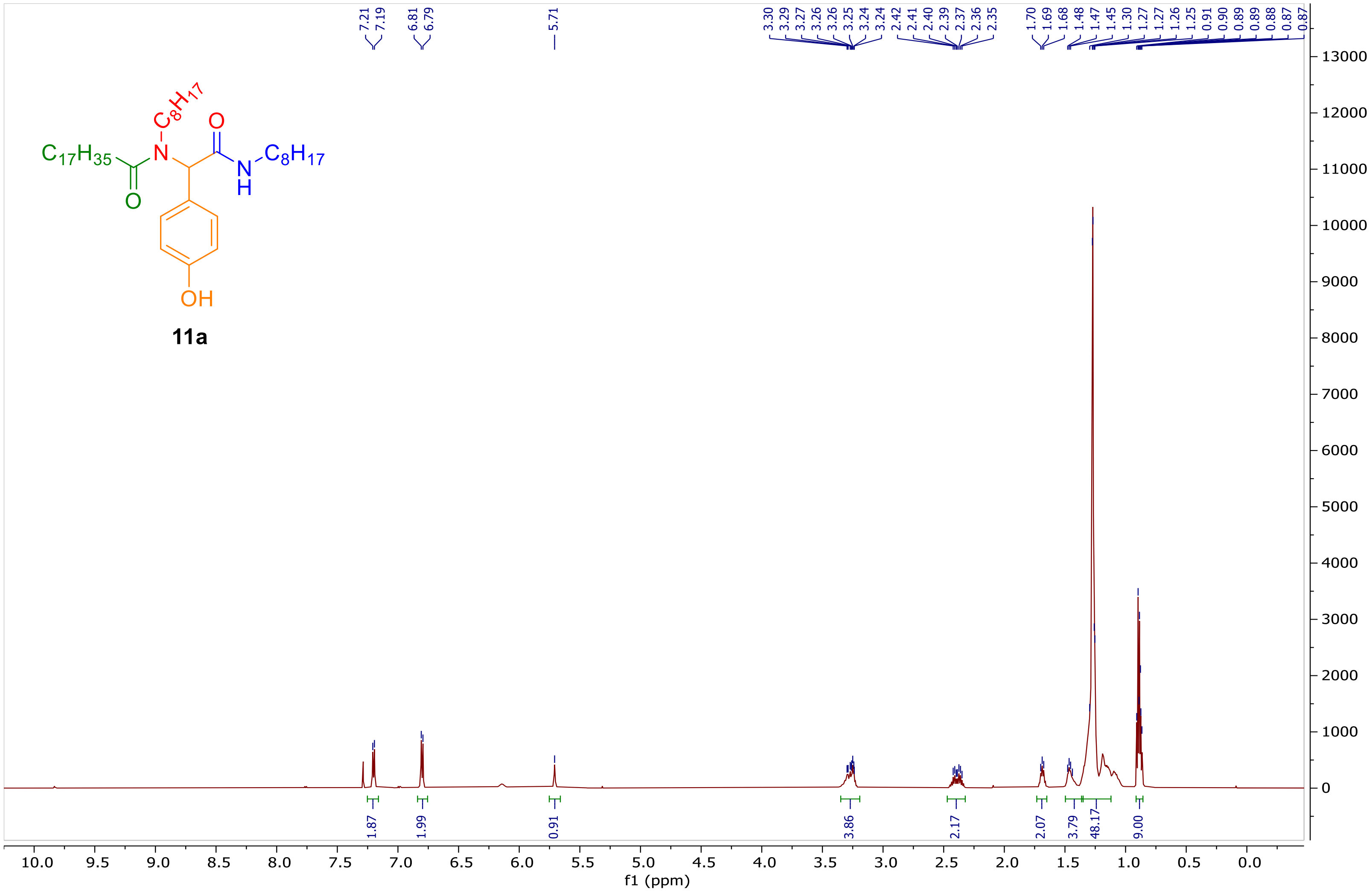

Figure S71. ^{1}H NMR (600 MHz, $CDCl_3$) Spectrum of compound **11a**.

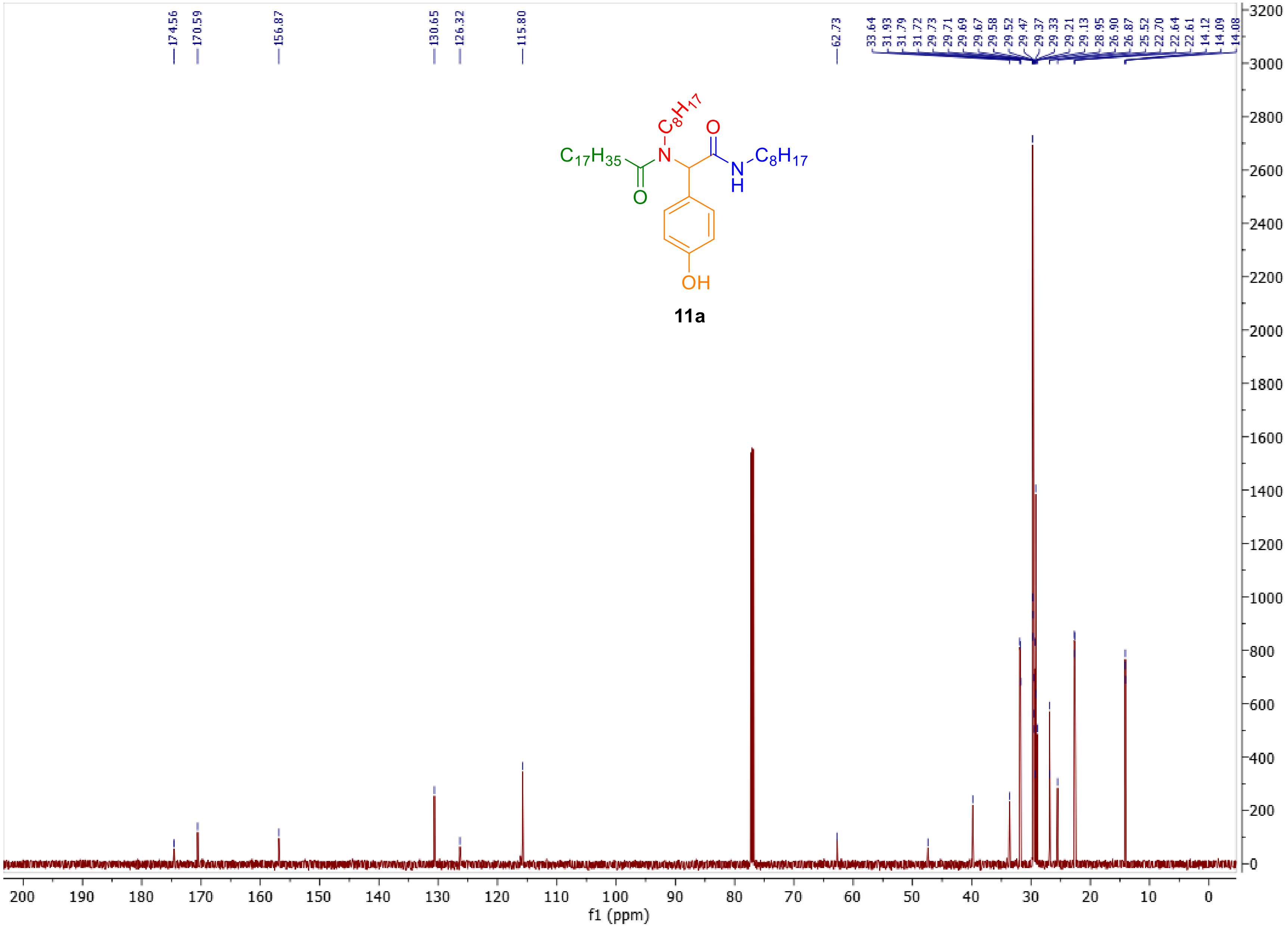

Figure S72. ^{13}C NMR (151 MHz, $CDCl_3$) Spectrum of compound **11a**.

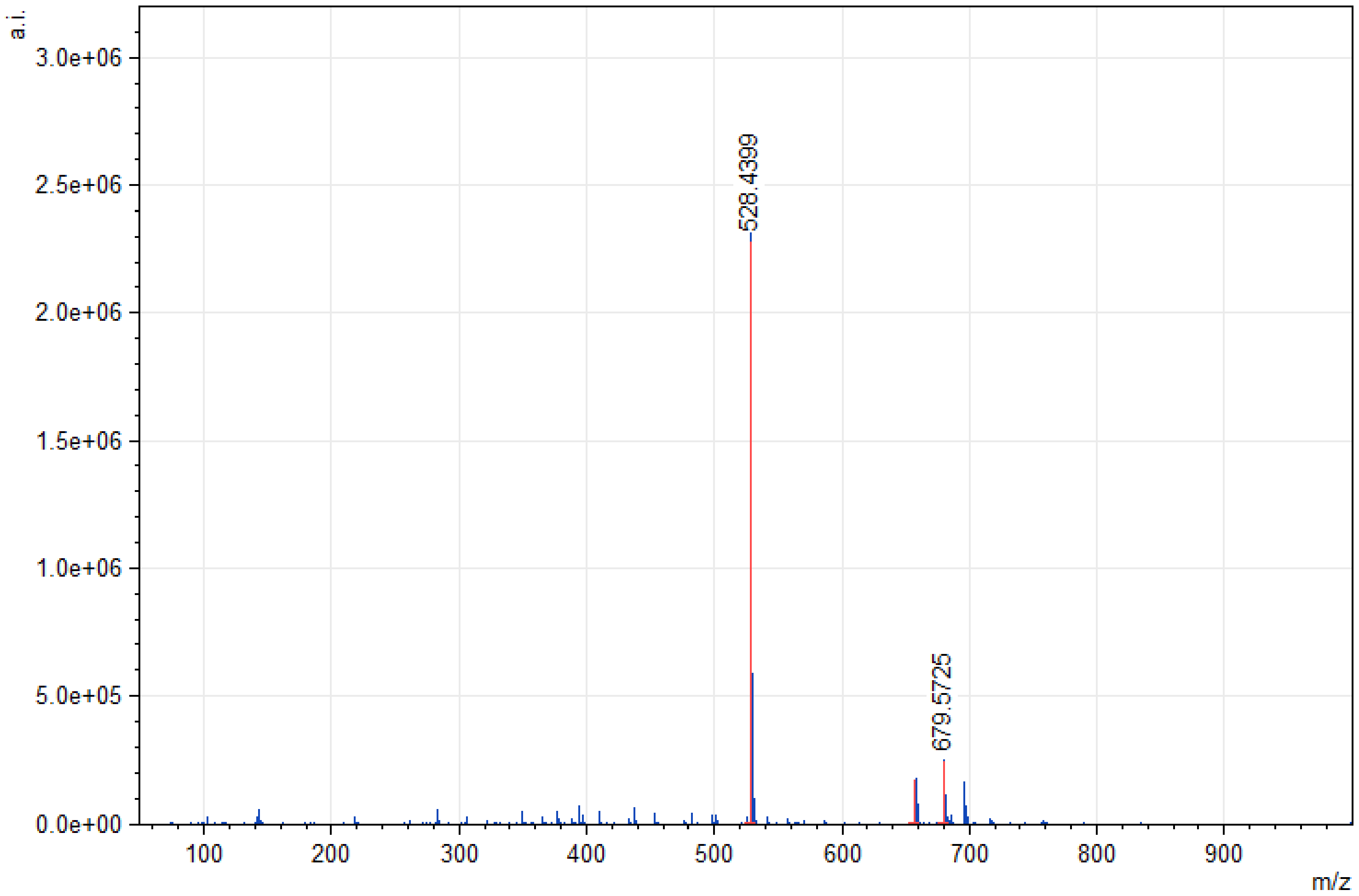

Figure S73. HRMS of compound **11a**.

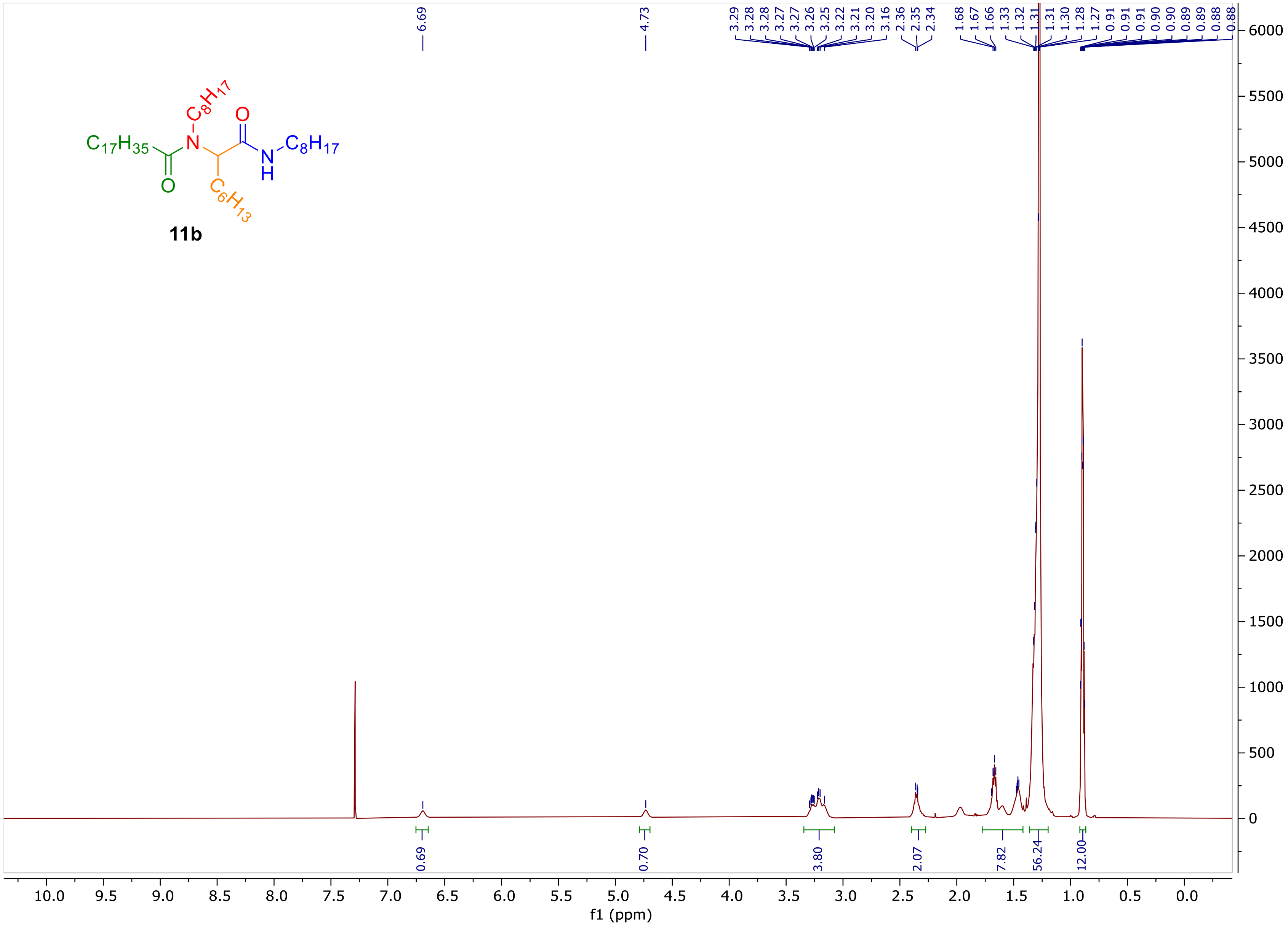

Figure S74. ^{1}H NMR (600 MHz, $CDCl_3$) Spectrum of compound **11b**.

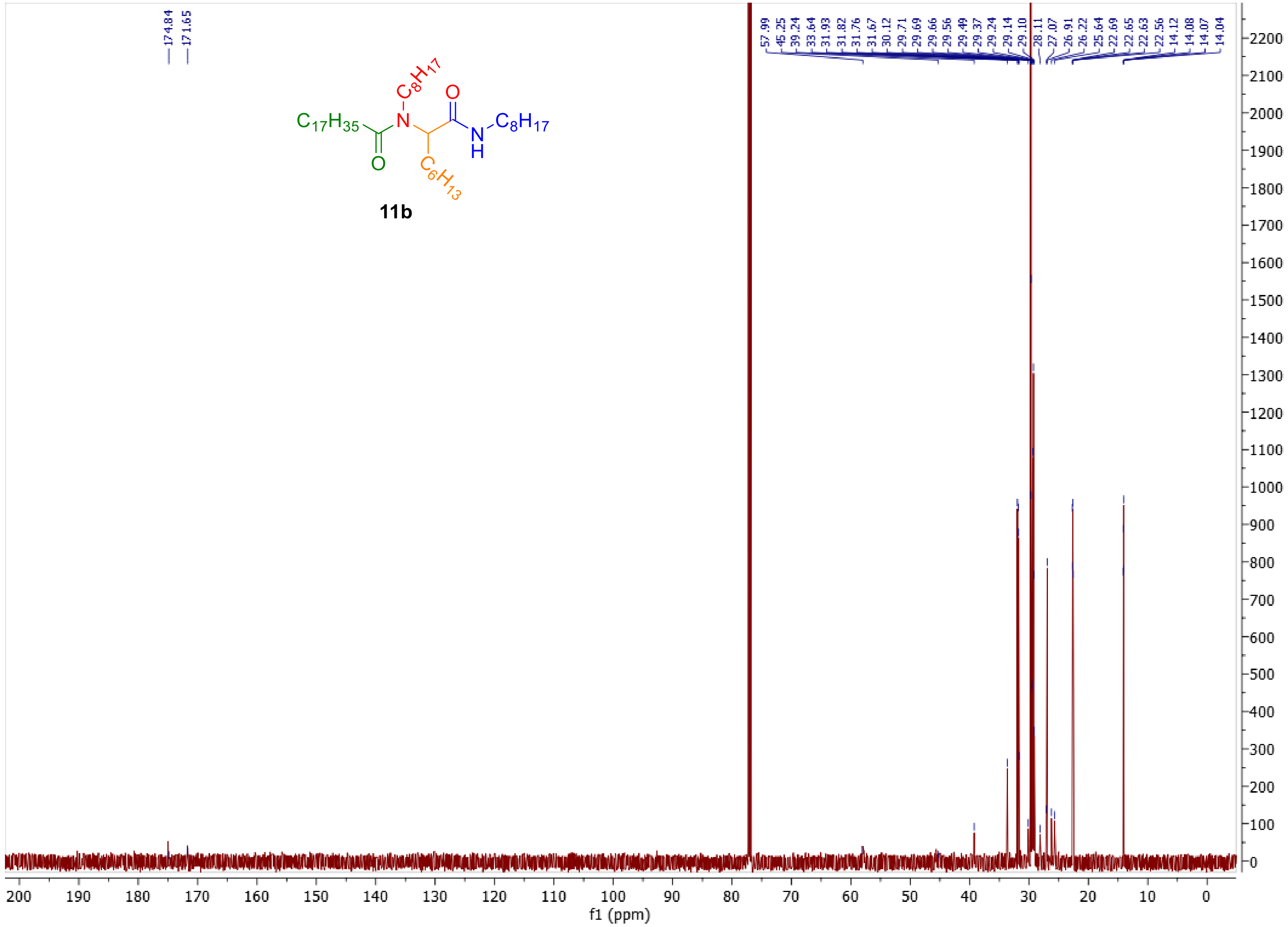

Figure S75. ^{13}C NMR (151 MHz, $CDCl_3$) Spectrum of compound **11b**.

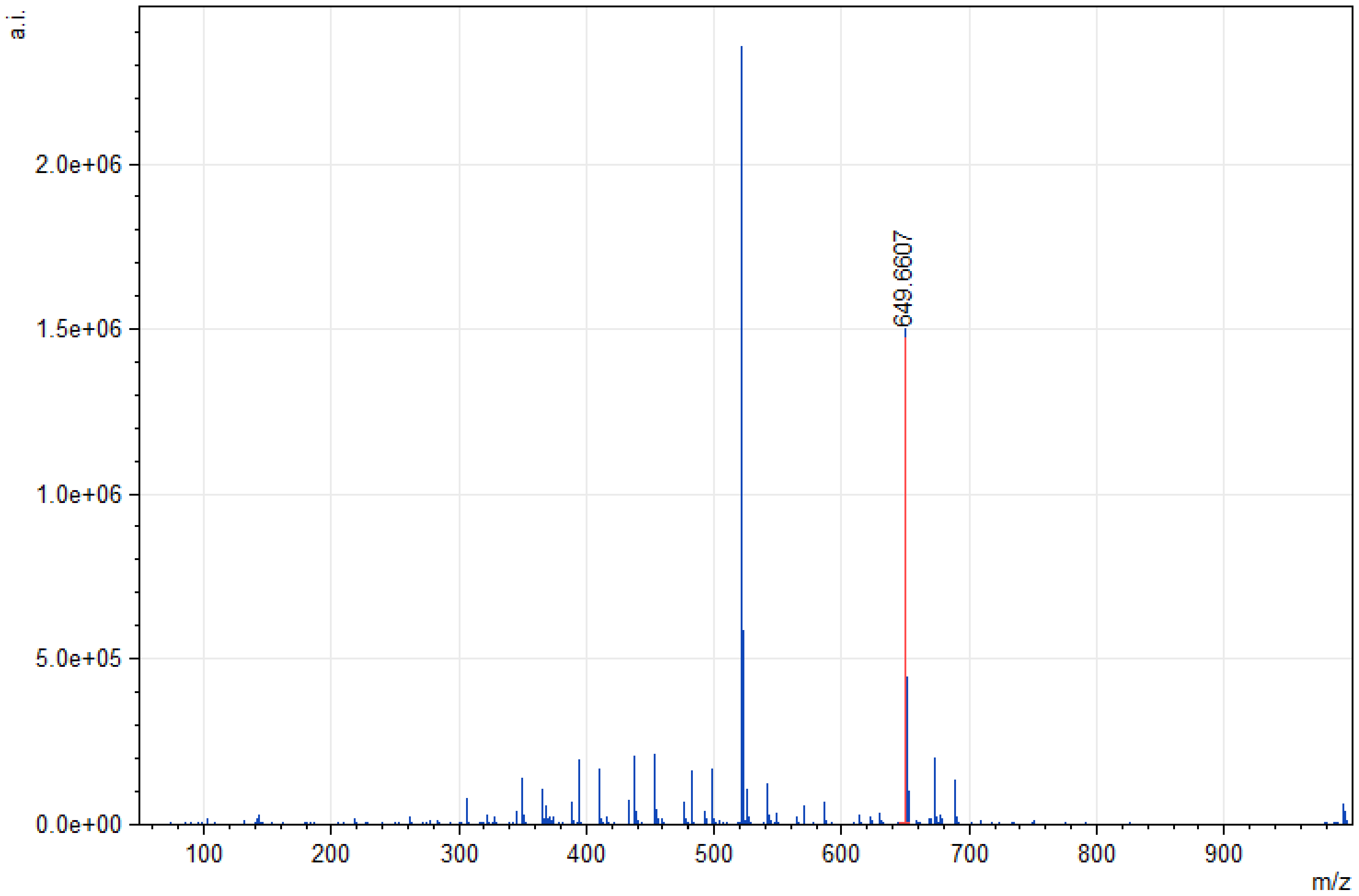

Figure S76. HRMS of compound **11b**.

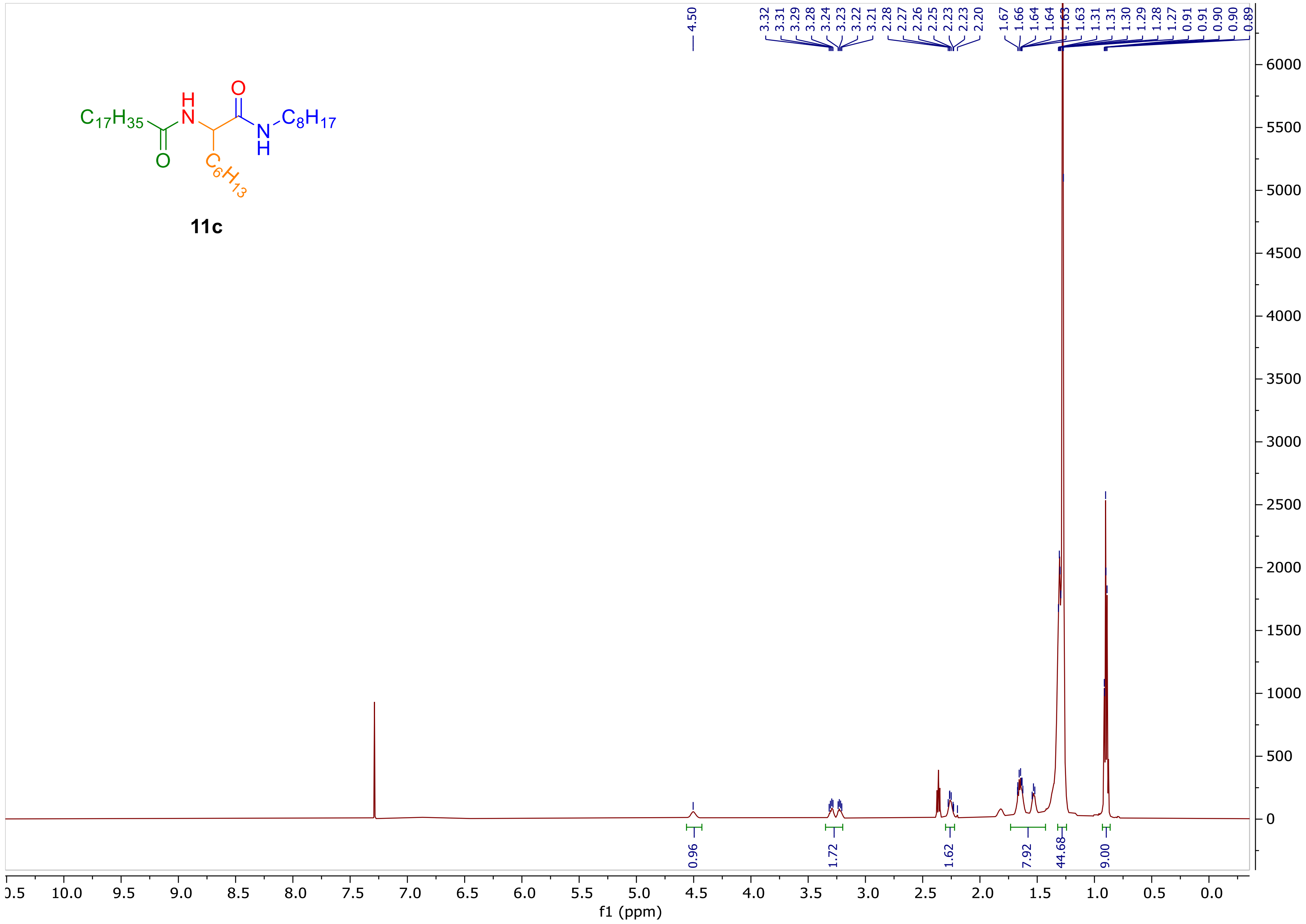

Figure S77. ^{1}H NMR (600 MHz, $CDCl_3$) Spectrum of compound **11c.**

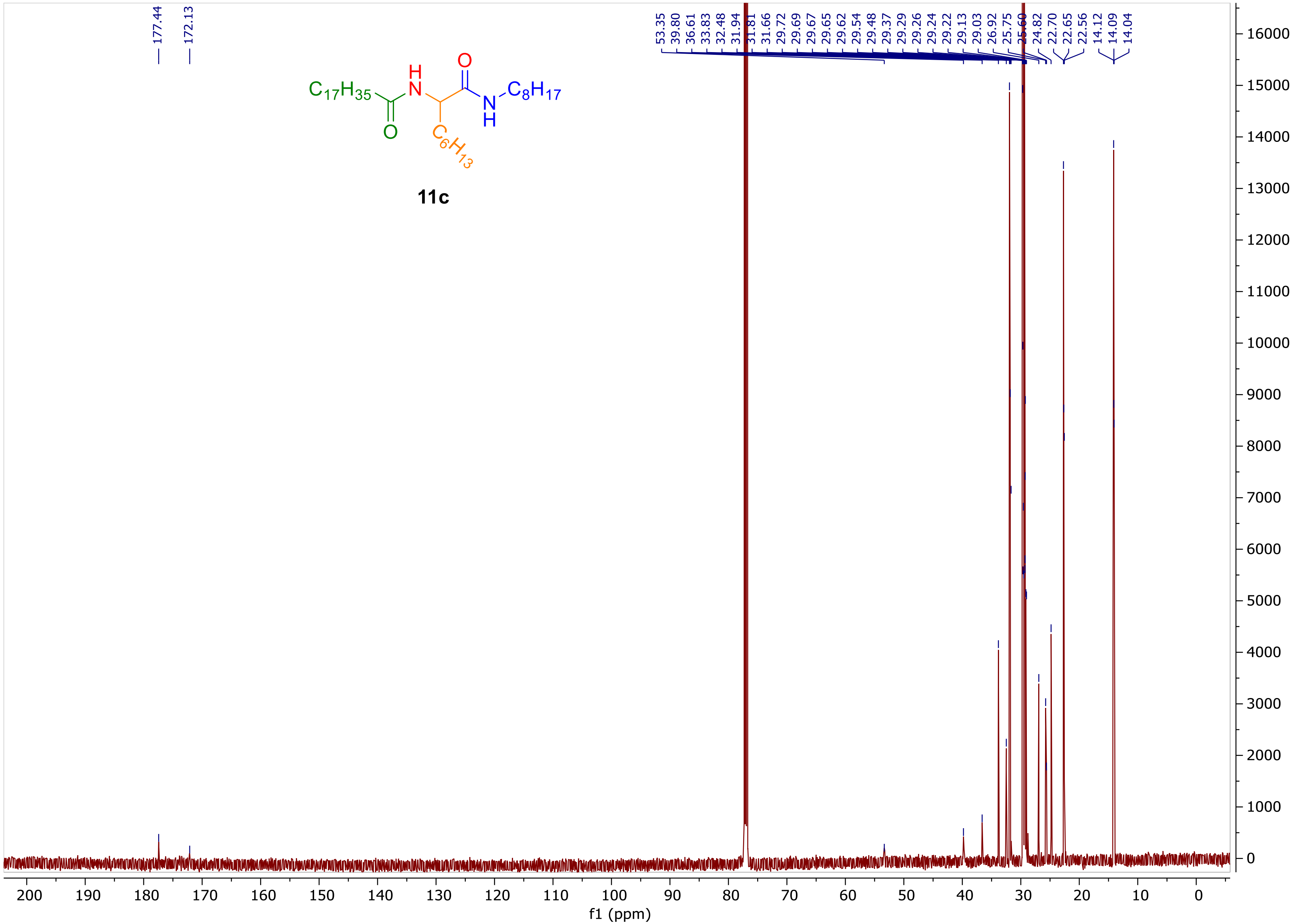

Figure S78. ^{13}C NMR (151 MHz, $CDCl_3$) Spectrum of compound **11c.**

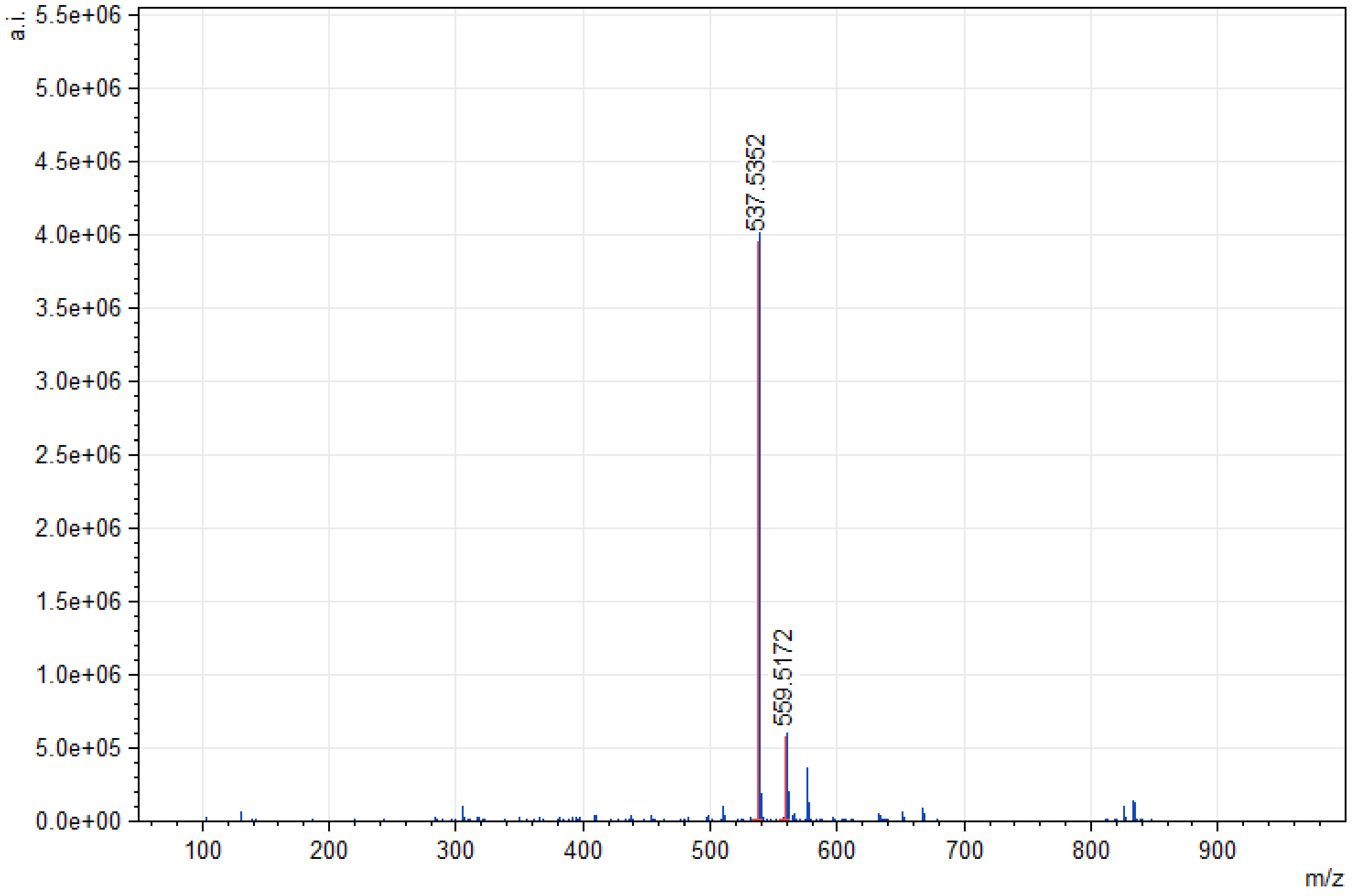

Figure S79. HRMS of compound **11c**.
